# The circulatory dynamics of human red blood cell homeostasis: Oxy-deoxy and PIEZO1-triggered changes

**DOI:** 10.1016/j.bpj.2022.12.038

**Published:** 2022-12-31

**Authors:** Virgilio L. Lew

**Affiliations:** 1 Physiological Laboratory, Department of Physiology, Development and Neuroscience, University of Cambridge, Downing Site, Cambridge, United Kingdom

## Abstract

The vital function of red blood cells (RBCs) is to mediate the transport of oxygen from lungs to tissues and of CO_2_ from tissues to lungs. The gas exchanges occur during capillary transits within fractions of a second. Each oxygenation-deoxygenation and deoxygenation-reoxygenation transition on hemoglobin triggers sharp changes in RBC pH, leading to downstream changes in ion fluxes, membrane potential, and cell volume. The dynamics of these changes during the variable periods between capillary transits in vivo remains a mystery inaccessible to study by current methodologies, a knowledge gap on a fundamental physiological process that is the focus of the present study. The use of a computational model of human RBC homeostasis of tested accreditation enabled a detailed investigation of the expected RBC changes during intercapillary transits, with results advancing novel insights and predictions. The predicted rates of relative RBC volume change on oxygenation-deoxygenation (oxy-deoxy) and deoxygenation-reoxygenation transitions were about 1.5%/min and −0.9%/min, respectively, far too slow to allow the cells to reach steady states in the intervals between capillary transits. The amplitude of the oxy-deoxy-reoxygenation volume fluctuations varied in proportion with the duration of the intercapillary transit intervals. Upon capillary entry, oxy-deoxy-induced changes occur concurrently with deformation-induced PIEZO1 channel activation, both processes affecting cell pH, membrane potential, and cell volume during intertransit periods. The model showed that the effects were strictly additive as expected from processes operating independently on the cell’s homeostatic fabric. Analysis of the mechanisms behind these predictions revealed, for the first time, the complex interactions between oxy-deoxy and ion transport processes that ensure the long-term homeostatic stability of RBCs for optimal gas transport in physiological conditions and how these may become altered in diseased states. Possible designs of microfluidic devices to test the model predictions are discussed.

## Significance

Few biological investigations have been pursued with comparable intensity and continuity over the last 130 years as the gas transport function of red blood cells. Against this background, it is surprising to find a major knowledge gap concerning the dynamics of the changes human red blood cells experience during the cyclical oxygenation-deoxygenation of hemoglobin in the circulation in vivo. A well-tested model of red cell homeostasis enabled a detailed in silico investigation of the expected changes predicting relentless cyclical variations in cell pH, membrane potential, and cell volume throughout the cell’s lifespan. Analysis of the mechanisms behind these predictions revealed the entangled web of transporter interactions at work. Suggestions for new experimental tests of the model predictions are discussed.

## Introduction

Red blood cells (RBCs) are the most abundant cells in the body. Their main function is to ferry oxygen from lungs to tissues and CO_2_ from tissues to lungs, a task each cell performs from about 1000 to 2000 times daily. The full extent of the gas exchange is accomplished during the fraction of second it takes each RBC to traverse a capillary. In the following 30 to 120 s between successive capillary transits, the hemoglobin within each cell remains in a nearly fully oxygenated or deoxygenated condition in the systemic arterial or venous circulation, respectively.

The binding and release of oxygen on capillary transits alters the proton binding properties of hemoglobin, causing sharp intracellular pH changes (1,2,3,4). The altered proton gradient across the RBC membrane triggers ion fluxes leading to secondary changes in RBC pH, membrane potential, volume, cytoplasmic free magnesium concentration, and other cell variables. The condition of RBCs kept in oxygenation (oxy) and deoxygenation (deoxy) steady states in vitro has been extensively investigated and the mechanisms accounting for the oxy-doxy differences fully elucidated (4,5,6,7,8,9,10,11,12,13,14,15,16,17,18).

The open questions concern the dynamics of the oxy-deoxy-elicited RBC changes under physiological conditions in the circulation in vivo, questions that remain inaccessible to experimental scrutiny. Specifically, can RBCs reach the oxy or deoxy steady states known from experiments in vitro within the range of time intervals between successive capillary transits in vivo? This is the main focus of the investigation reported here.

Concurrent with the oxy-deoxy effects, the squeeze RBCs experience upon each capillary entry opens mechanosensitive PIEZO1 channels in the RBC membrane for a fraction of a second, allowing a brief and transient net calcium influx to trigger a complex cascade of downstream ion fluxes and minute volume changes (5,19,20,21,22,23,24,25). How the combined effects of oxy-deoxy transitions and PIEZO1 activation during the first second of capillary ingress influence the time course of RBC changes in the time intervals between capillary transits in vivo is the additional quest in the present study.

To address these questions, use of a tried and tested model of RBC homeostasis (5,19,26,27,28,29) allowed an in-depth investigation of the expected kinetics of RBC homeostatic changes in the circulation in vivo. The results predicted a highly dynamic condition with continuously changing RBC volumes, pH, and membrane potentials throughout the lifespan of the cells, with values oscillating within margins well short of those characterized in vitro for RBCs in oxy or deoxy pump-leak steady states.

## Materials and methods

### The RBC model (RCM)

The model version used for the current investigation (5,19) is available for download with open access from a GitHub repository (https://github.com/sdrogers/redcellmodeljava) together with a comprehensive user guide and tutorial. An updated file with the governing equations of the model is offered here as supporting material. The model operates as a program within the JAVA environment. The name of the program, “RCM^∗^.jar”, contains coded information on date and update status and is best retained unchanged. The version used for the simulations reported here was “RCM_8560ca5.jar”.

Model simulations follow user-generated instructions recorded in editable protocol files (^∗^.txt). Protocols start by defining the constitutive properties of the RBC under study in an initial oxy steady-state condition, the reference state (RS). The RS entry is followed by sequences of dynamic state instructions designed to emulate stages in experiments or physiological processes. The results are reported in ^∗^.csv file format. The columns in the ^∗^.csv files display all the variables of the system. The rows report their changing values with time. Running time is always displayed in column one (in min). The RS condition of the RBC suspension used for the simulations reported here corresponds with that of an oxy-RBC with a mean cell hemoglobin concentration of 34 g/dL in a pump-leak steady state suspended in a buffered plasma-like medium.

### Simulating the oxy-deoxy-reoxygenation (reoxy) circulatory dynamics of RBC homeostasis

The physiological link between oxy-deoxy transitions and RBC homeostasis is the isoelectric point of hemoglobin (Hb), pI. The model equations translate the instant pI changes induced by oxy-deoxy transitions (1,2) into cell pH changes, as required by charge conservation (30,31). The red cell model was therefore configured to represent such transitions by using the measured pI(0°C) change from 7.2 to 7.5 (1,2). Preservation of electroneutrality required that the net charge on Hb, nHb, remained invariant as pI changed (30). The link between nHb, pI, and cell pH is given by the Dalmark equation:

nHb=α(pl−pH),

where α is the slope of the proton titration curve of Hb in intact RBCs (α = −10 Eq/(mol^∗^Δ(pH – pI)) for HbA).

The following equation preserves nHb during oxy-deoxy transitions:

$$nHb=\alpha \left({pl}_{oxy}-{pH}_{oxy}\right)=\alpha \left({pl}_{deoxy}-{pH}_{deoxy}\right)\text{.}$$

From this equation, we derive the new cell pH, pH_deoxy_, after an oxy-deoxy transition, as follows:

(1) $$pH_{deoxy}=pH_{oxy}+\left(pl_{deoxy}-pl_{oxy}\right)\text{,}$$

and the initial pH on reoxy, pH_oxy_, will be

(2) $$pH_{oxy}=pH_{deoxy}+\left(pl_{oxy}-pl_{deoxy}\right)\text{.}$$

Following the initial ΔpHi after each transition, the model computes the evolution in time of all the homeostatic variables of the system. The parameter values used for the current simulations were all experimentally determined within coefficients of variation of between 5% and 15% (1,2,13,14,15,30,31,32,33,34,35).

### Simulating the PIEZO1 triggered changes in RBC homeostasis

Concurrently with the oxy-deoxy transitions on capillary entry, the subsecond open state of the PIEZO1 channels allows a sharp inflow of CaCl_2_ and water driven by the huge inward electrochemical gradient for Ca^2+^. Calcium influx, in turn, elevates [Ca^2+^]_i_ to levels that activate Ca^2+^-sensitive K^+^ channels (Gardos channels, KCNN4, (36,37,38,39)), causing secondary loss of KCl and water. The Ca^2+^ effects are cut short by Ca^2+^ extrusion through the powerful plasma membrane calcium pump (plasma membrane calcium pump 4b, (40,41,42,43)) rapidly restoring baseline [Ca^2+^]_i_ levels. The Cl^−^ gained during the initial CaCl_2_ inflow is restored by proton-driven Cl^−^ efflux via the Jacob-Stewart mechanism with parallel osmotic-driven cell volume reduction. The net loss of KCl and water during the brief open period of the Gardos channels generates a tendency for cell volume to decrease between consecutive capillary transits, a tendency that declines with cell age because of the exponential fall in the calcium extrusion capacity of the pump (43,44). These sequential events generate an up-down biphasic volume response, with RBC volume increasing by up to 0.005% at the peak and falling below baseline levels by less than 0.0001% within a minute or two. These are infinitesimal volume displacements but have the potential to increase RBC density over myriad capillary transits as the cells age in the circulation (5,19). This response and its complex mechanism have been analyzed and reported in detail before but only for oxy conditions (5). The new question addressed here is whether PIEZO1 and oxy-deoxy processes interact and influence each other during intercapillary transits.

### Representation of the extracellular medium in the simulations

In vivo, plasma pH is essentially the same for arterial or venous blood because CO_2_ volatility and the respiratory apparatus effectively provide unlimited buffering capacity to the plasma, with minor contributions of histidine residues from plasma albumin (10). Plasma pH invariance can only be represented in a mass-conservation model structured as a closed cell-medium two-compartment system by running the simulations at a vanishing low cell volume fraction, approximating the condition of an open system with a constant medium composition.

**Figure 1:**
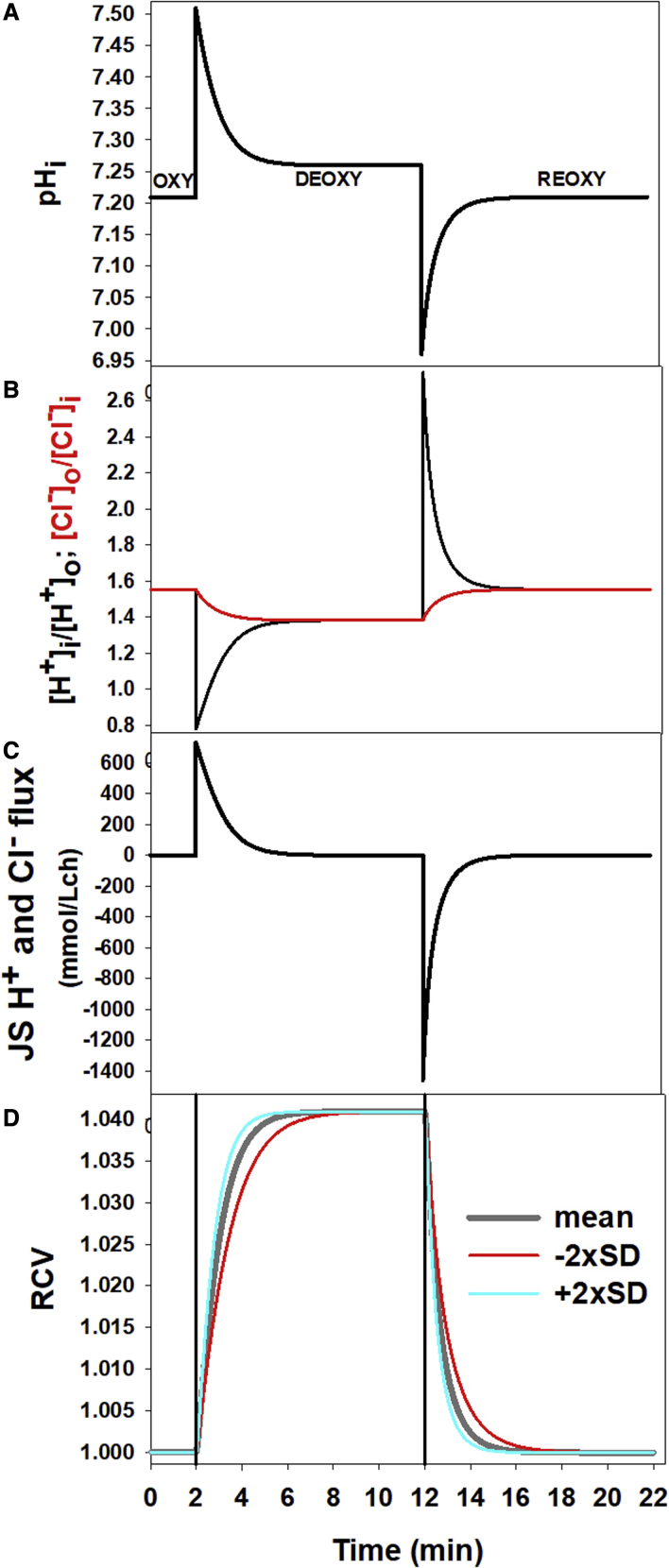
Time course of the changes in RBC homeostatic variables triggered by oxy-deoxy transitions on the isoelectric point of hemoglobin (pI). The time scale for this simulation was chosen to allow the variables to reach effective steady states. (*A*) The first effect of the instant (subsecond) oxy-to-deoxy pI transition is a sharp increase in cell pH followed by a slower recovery toward a deoxy steady state above oxy baseline levels. Upon reoxy, the peak excursion from the deoxy level is larger, but the slow return to the oxy steady state is shorter, establishing a kinetic asymmetry between oxy and deoxy transitions. (*B*) Deoxy lowers the H^+^ and Cl^−^ concentration ratios across the RBC membrane relative to their initial oxy equality. The equalities are gradually restored to the oxy level upon reoxy. (*C*) H^+^ and Cl^−^ fluxes through the Jacob-Stewart (JS) mechanism operating as an H^+^:Cl^−^ cotransporter (5,26,45). The areas between oxy-deoxy and deoxy-reoxy steady states are the same as expected for balanced fluxes returning to initial conditions. (*D*) The maximal amplitude predicted for the oxy-deoxy volume displacements amounts to about 4%. The mean curve (*black*) was computed using the measured mean rate constant for JS-mediated H^+^ and Cl^−^ fluxes. The thin lines (*red and cyan*) were computed using rate constant values set at twice the measured SD of the rate constant distribution (34). Note that the predicted time course to steady-state volume is around 4–5 min, far longer than most intercapillary transit times in the 0.5 to 2.0 min range. To see this figure in color, go online.

**Figure 2:**
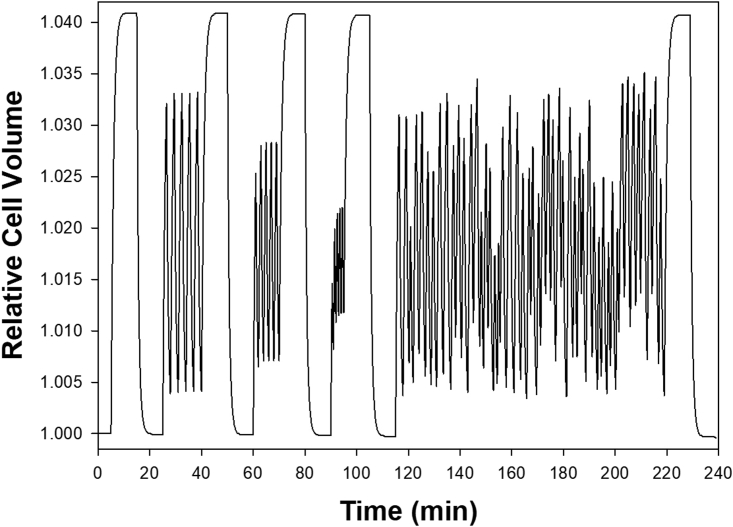
Relation between predicted amplitudes of RBC volume changes during oxy-deoxy-reoxy cycling and duration of the intercapillary transit intervals. In the protocol for this simulation, oxy-deoxy cycling trains were separated by 10 min cycling breaks to allow easy visual comparisons of cycling amplitudes with oxy-deoxy steady-state volume levels. From left to right, the duration of the first three intertransit intervals was set to 1.5, 1.0, and 0.5 min. In the fourth sequence, intertransit interval times were set to vary at random between 0.5 and 1.5 min. The random cycling train was explored for up to 10 days in additional simulations, with no detectable shifts in the interspersed steady-state volume levels recorded at regular intervals.

**Figure 3:**
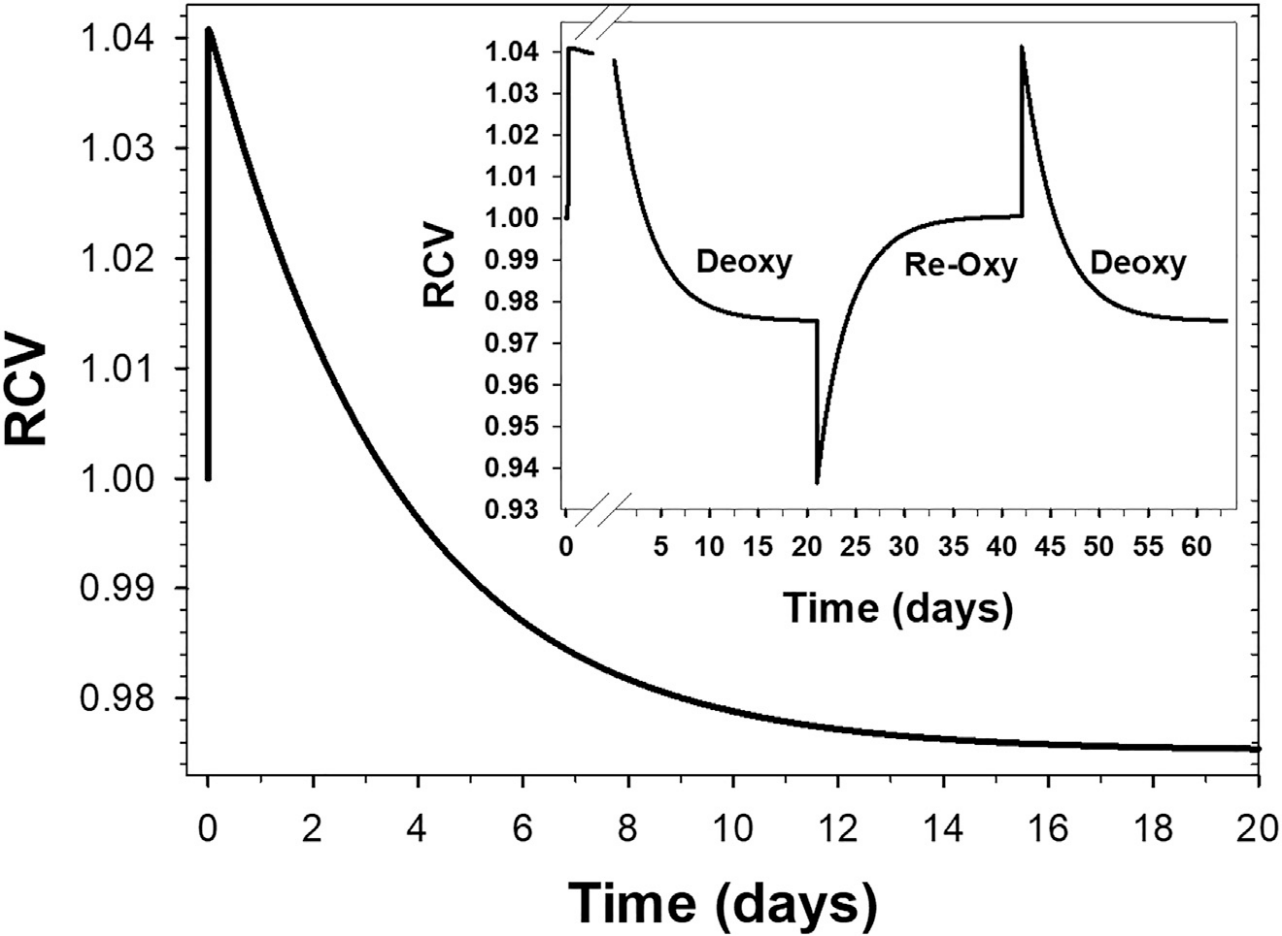
Exploring the long-term evolution of the volume changes induced by oxy-deoxy transitions in human RBCs kept under deoxygenated conditions. After the initial volume increase reported in Fig. 1*D*, the model predicted a steady volume decline at rates increasing from 1 to 2 × 10^–5^ per min. The final steady volume level at day 20 was 0.975, a 6.6% fall from the initial physiological quasi steady state of 1.041. On an expanded initial time scale (*inset*), the declining pattern is clearly noticeable within the first 2–3 days of the deoxy transition. The sequential deoxy-reoxy-deoxy transitions in the inset document the full reversibility of the homeostatic conditions of RBCs transitioning between initial quasi and final true steady states. To see this figure in color, go online.

**Figure 4:**
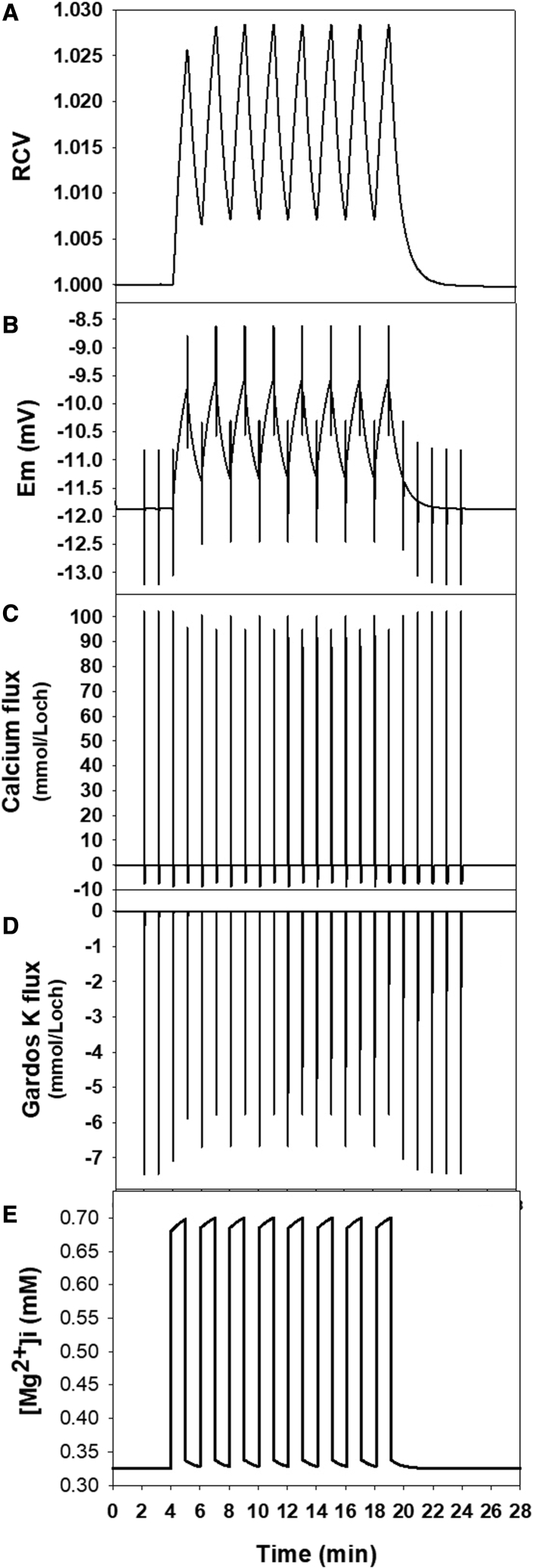
The mechanism of the PIEZO1-triggered RBC volume changes during inter-capillary transits. (*A*–*C*) The sequential steps leading to the up-down biphasic volume response (*D*) elicited by the RBC deformations that activate PIEZO1 channels on capillary ingress. The insets within each panel show detailed changes on expanded *x* or *y* scales. The open state of the PIEZO1 channels was set to 0.4 s in the simulations. The cyan curves in (*A*)–(*C*) follow the *y* axis changes on the same common time scale as (*D*). (*A*) Calcium influx and pump-mediated extrusion. The cyan curve and the inset on an expanded time scale show a brief sharp positive net Ca2^+^ influx during the open state, instantly reversing to a negative pump-mediated Ca2^+^ extrusion in two phases: a sustained efflux of about −7 mmol/Loch by a [Ca2^+^]i-saturated pump followed by a brief [Ca2^+^]i-desaturation efflux, all essentially over in 6–10 s. (*B*) K^+^ fluxes through Ca2^+^-activated Gardos channels. After the sharp initial K^+^ efflux peak, K^+^ efflux returns to the zero baseline in less than 6 s following the [Ca2^+^]i-desaturation kinetics of the channels (49). During this period, the cells experience a net loss of KCl and water. (*C*) Chloride and proton fluxes through the JS complex. The initial Cl^−^ gain, twice that of Ca2^+^, is rapidly extruded as an H^+^:Cl^−^ cotransport through the JS complex. However, the equality of the proton and chloride concentration ratios at equilibrium is approached much slower as shown in this inset and in that of (*D*) on a vastly expanded *y* axis scale. (*D*) Predicted volume change following a subsecond PIEZO1 channel activation. Note that the net loss of KCl and water during the Gardos channel activation period (Fig. 4*B*) leads to cell dehydration during the longer-lasting intertransit periods. Despite the infinitesimal scale of the predicted volume changes relative to those triggered by oxy-deoxy cycling (Fig. 2), only PIEZO1-mediated ones have the potential to cause cumulative RBC hydration changes in the circulation.

**Figure 5:**
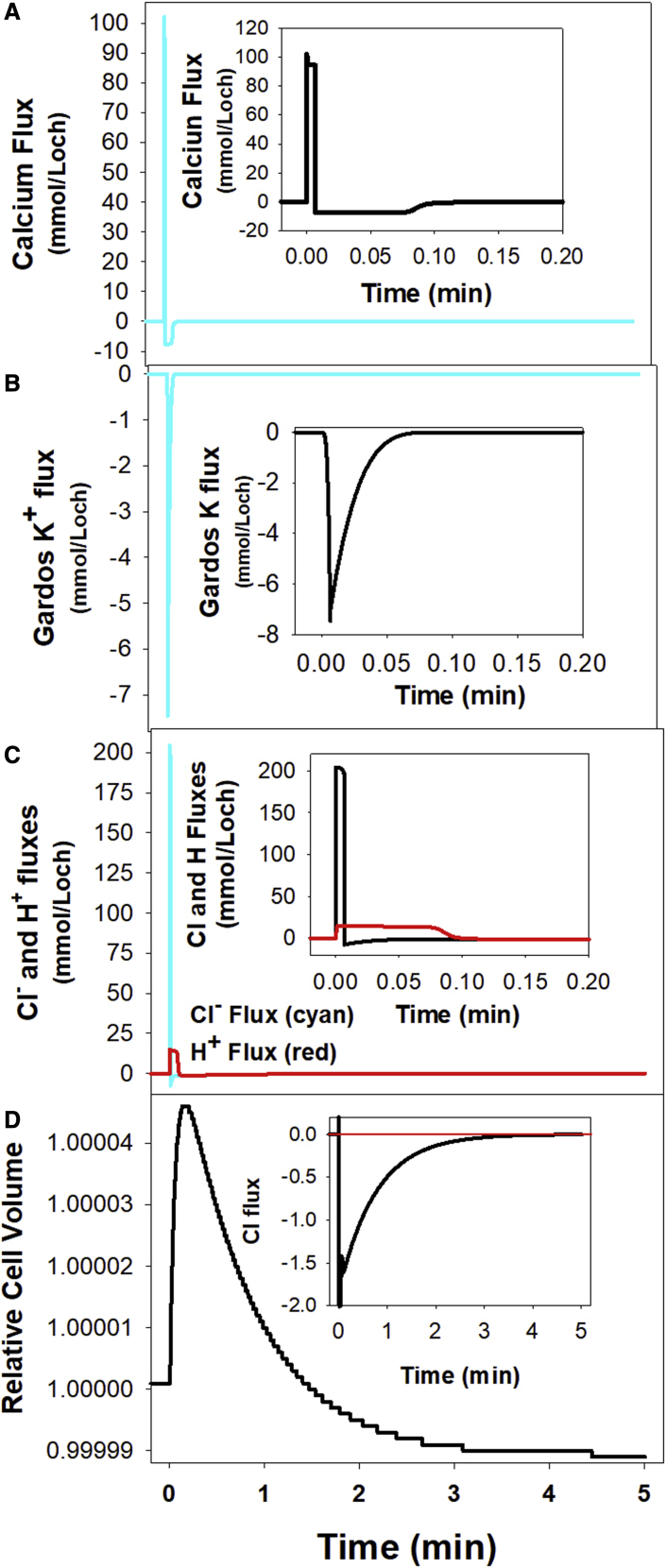
Interactions between oxy-deoxy- and PIEZO1-triggered processes during intercapillary transits. In order to explore mutual interactions, the protocol for this figure was designed to show 23 sequential 1 min intercapillary transits with PIEZO1 openings of 0.4 s on capillary ingress and 16 overlapping instant oxy-deoxy transitions in the middle of the PIEZO1 train (see supporting material S1 for protocol details). The selected variables for (*A*)–(*D*) were the only ones that showed detectable interactive influences. (*A*) The minute PIEZO1 volume changes (Fig. 3*D*) do register but cannot be discerned on the scale of the oxy-deoxy-induced changes shown here. (*B*) The amplitude and brevity of the PIEZO1-induced membrane potential changes remains unaltered when superimposed on those of the much slower oxy-deoxy membrane potential transitions. (*C*) Note that the Ca2^+^ influx peaks appear reduced on deoxy relative to reoxy and so do the plasma membrane calcium pump-mediated dips (Fig. 3*A*). (*D*) Gardos channel-mediated K^+^ fluxes are similarly reduced on deoxy, suggesting a clear link to the reduced Ca2^+^ influx noted in (*C*). (*E*) The relatively large oxy-deoxy-induced changes in [Mg2^+^]i are fully reversible. There are no detectable [Mg2^+^]i responses to PIEZO1-induced changes in the absence of oxy-deoxy transitions.

The protocols used for the simulations shown in Figure 1, Figure 2, Figure 3, Figure 4, Figure 5 are shown sequentially within a pdf file as supporting material SM1. SM2 presents a new version of the governing equations of the red blood cell model, updated to include all equations associated with oxy-deoxy transitions and cytoplasmic magnesium buffering used within RCM_8560ca5.jar in the present study. The SM1 and SM2 files are combined into a single PDF file.

## Results

The oxy-deoxy-induced changes in RBC homeostatic variables are considered first, followed by those elicited by PIEZO1 activation, in isolation and in combination with oxy-deoxy changes. The emphasis throughout the presentation of the model results is on the mechanisms behind the predicted effects.

### The kinetics of oxy-deoxy-elicited changes in RBC homeostasis

The panels of Fig. 1 illustrate, from top to bottom, the sequential changes in selected RBC variables triggered by oxy-deoxy-reoxy transitions, as predicted by the model. The sudden initial pI change elevates cell pH (Fig. 1 *A*; Eq. 1), driving all subsequent downstream changes in homeostatic variables (Fig. 1, *B* to *D*). Deoxy elevates cell pH by about 0.3 units (Fig. 1 *A*), reflecting a sharp initial reduction in cytoplasmic [H^+^]_i_ from ∼60 to ∼30 nM.

At constant extracellular pH, the suddenly increased inward proton gradient (Fig. 1 *B*, *black curve*) activates a large net H:Cl influx through the Jacob-Stewart mechanism (Fig. 1 *C*) attempting to equalize the displaced proton and chloride concentration ratios toward the zero net flux Jacob-Stewart equilibrium condition, [H^+^]_i_/[H^+^]_o_ = [Cl^−^]_o_/[Cl^−^]_i_ (Fig. 1 *B*). Net chloride influx, in turn, elevates cell osmolarity, thus increasing cell volume by up to about 4% in the deoxygenated steady state (Fig. 1 *D*). The deoxy-induced volume increase is therefore the direct result of the Jacob-Stewart mechanism attempting to restore the [Cl^−^]_o_/[Cl^−^]_i_ = [H^+^]_i_/[H^+^]_o_ equality. Reoxy reverses the direction of all these processes, starting with a reoxy-induced decrease in pH (Eq. 2; Fig. 1 *A*).

This sequence sums up the known mechanisms responsible for the effects of oxy-deoxy transitions on RBC pH and volume. The predicted steady-state pH and volume levels follow closely the experimentally measured ones (30,31). The experimental results were obtained using RBC suspensions at higher cell volume fractions than those simulated here. Whereas at low cell fractions, pH and volume changes are maximally absorbed by the cells, at higher cell fractions, the spread of changes between cells and medium reduces their amplitude in the cells, thus explaining why the experimental and in vivo volume increases in deoxy states remain close to, but below, the ∼4% predicted maxima.

The predictions in Fig. 1 show, for the first time, the dynamic responses of the main variables controlling RBC volume in response to oxy-deoxy-reoxy transitions as expected to take place in the circulation in vivo.

The rates at which RBC volumes approach steady states after oxy-deoxy-reoxy transitions were estimated from exponential fits to the red cell volume (RCV) curves in Fig. 1 *D*. The oxy-deoxy RCV transition rate was about 1.5%/min, and the deoxy-reoxy rate was about −0.9%/min. This asymmetry in magnitude can be traced to differences in the initial peak displacements of cell pH (Fig. 1 *A*), of [H^+^]_i_/[H^+^]_o_ (Fig. 1 *B*), and of Cl^−^ flux (Fig. 1 *C*), the sequential drivers of the processes leading to the volume change (Fig. 1 *D*). The important new insight here is that the rates of volume change are too slow to allow steady states to be approached between capillary passages unless blood flow becomes locally arrested for longer periods (45,46).

The simulations in Fig. 2 illustrate the extent to which the amplitude of the RBC volume changes may depart from oxy-deoxy steady-state values in circulatory conditions. The figure shows, from left to right, three sequential trains of oxy-deoxy cycles set for intertransit intervals of 1.5, 1, and 0.5 min. It can be seen how steeply the amplitude of the volume excursions becomes reduced the shorter the intertransit intervals. The trains are shown separated by 10 min segments in oxy or deoxy conditions to allow for a direct visual comparison of amplitudes relative to oxy or deoxy steady-state levels.

The fourth train shown on the right of Fig. 2, designed with a protocol of stochastic intertransit durations between 0.5 and 1.5 min, represents the more realistic in vivo condition for individual RBCs traversing variable distances between sequential capillary circuits in the systemic circulation. In days-long simulations (data not shown), the interspersed 10 min comparative steady-state volume levels remained invariant, suggesting that oxy-deoxy cycling generates no cumulative changes on the basic homeostatic configuration of the RBCs in the circulation a critical distinction from the PIEZO1-elicited effects (19).

### Is the deoxy steady state a real steady state?

An unexpected prediction of the model was that the rapid volume increase induced by deoxy was not toward a real steady state. On the time scale of Figs. 1 *D* and 2, it was very hard to discern that the deoxy plateau described as a steady state was not quite steady. Over much longer time scales (Fig. 3), the relative cell volume of RBCs kept in deoxy conditions declined slowly but steadily toward a real pump-leak steady state below that of the oxy RBCs, a relative volume decrease of about 7%. It can be seen that immediately after an oxy-deoxy transition, the apparent steady-state volume response shown in Fig. 1 *D* remains hidden within the peak volume increase when displayed on the vastly expanded time scale of Fig. 3.

Exploring this response further over extended periods of time (Fig. 3, *inset*), the results showed fully reversible long-term transitions between real steady states through transient peak-induced breaks at the oxy-deoxy and deoxy-reoxy turning points. Although the short-term deoxy quasi steady states shown in Figs. 1 *D* and 2 do not represent real steady states, they do represent the only condition of physiological relevance for determining the direction of volume changes during oxy-deoxy transitions in vivo within realistic intercapillary transit times (Fig. 2).

### The kinetics of PIEZO1-elicited changes in RBC homeostasis following capillary ingress

The panels in Fig. 4 sum up, from top to bottom, the sequence of PIEZO1-triggered changes following a brief subsecond open state (5,24,47). The use of a common time scale for the three sequential processes (Fig. 4, *A*–*C*, *cyan curves*) exposes more clearly how sharp and brief transport changes can elicit delayed volume responses (Fig. 4 *D*). The slow volume response results from the extremely low constitutive cation permeability of the RBC membrane rate limiting the restorative net salt and fluid movements. The insets in Fig. 4, *A*–*C*, show the initial displacements on a largely expanded *x* axis time scale for detail.

The mechanism shaping the volume response to PIEZO1 activation starts with a massive calcium influx driven by its huge inward electrochemical gradient (Fig. 4 *A*) during the subsecond duration of the PIEZO1 channel openings. Elevated [Ca^2+^]_i_ stimulates the plasma membrane calcium pump to rapidly extrude the calcium gained as shown by the area within the negative flux dip in Fig. 4 *A*, with extrusion accomplished in about 0.1 min (Fig. 4 *A*, *inset*). During the period of elevated [Ca^2+^]_I_, Gardos channel activity mediates a large net K^+^ efflux (Fig. 4 *B*), leading to a net loss of KCl and water from the cell. The Cl^−^ gained during the initial CaCl_2_ influx is rapidly extruded by the Jacob-Steward mechanism (Fig. 4 *C*) operating as an electroneutral Cl^−^:H^+^ cotransport (26,48). The net loss of KCl and water during the brief period of Gardos channel opening leads to a slow cell volume readjustment toward a slightly dehydrated level after each capillary transit, amounting to a relative volume decrease of less than 0.00001% (Fig. 4 *D*). This slow volume change is led by a minute residual Cl^−^ efflux shown on a vastly expanded *y* axis scale in the inset of Fig. 4 *D* but with the capacity to cause cumulative volume changes in association with age-dependent declines in plasma membrane calcium pump activity (5,19).

### Exploring interactions between oxy-deoxy- and PIEZO1-mediated processes

Within the first second of capillary entry, gas exchange is completed (49,50,51), and PIEZO1 channels open and close (19). Fig. 5 shows how these two transients affect selected homeostatic variables in the intervals between capillary transits. The simulations were designed to optimize comparisons between separated and overlapping responses on the same time scale. The results in Fig. 5 sum up the main results and conclusions on mutual influences derived from a much wider investigation.

Fig. 5, *A* and *E*, show changes elicited by oxy-deoxy cycling on variables apparently unaffected by PIEZO1 activity. Whereas PIEZO1 has no real effect on the [Mg^2+^]_i_ transitions (Fig. 5 *E*), the minute size of the PIEZO RCV signal (Fig. 4 *D*) is present but remains hidden on the scale of the oxy-deoxy RCV displacements (Fig. 5 *A*). Fig. 5, *B*–*D*, show variables where apparent interactions could be detected.

Fig. 5*B* shows the composite effects of both processes on the RBC membrane potential. PIEZO1 activation causes a brief depolarization during CaCl_2_ influx because Cl^−^ gain reduces the [Cl^−^]_o_/[Cl^−^]_I_ ratio. As soon as the Ca^2+^ gain activates the Gardos channels increasing the K^+^ permeability (Fig. 4 *B*), the membrane potential shifts toward E_K_, hyperpolarizing the membrane. As shown in the insets of Fig. 4, *B* and *C*, this biphasic response is nearly completed within 0.1 min, appearing as needle-sharp membrane potential deflections of about 2 mV amplitude on the common time scale of Fig. 5.

Deoxy-reoxy cycles, on the other hand, generate slower depolarization-repolarization transients following the slower proton ratio displacements shown in Fig. 1, *B* and *C*. The PIEZO1 E_m_ signals remain unaltered when superimposed on the oxy-deoxy E_m_ signals, indicating the noninteractive additivity of the two processes on this variable.

The calcium flux pattern generated by PIEZO1 activation (Fig. 5 *C*) becomes slightly altered when superimposed on the deoxy-oxy signals. Deoxy reduces both the calcium influx peak through PIEZO1 and the pump-extrusion dip. Reoxy reverses these changes. Model analysis of this interaction offered a straightforward explanation: the depolarization associated with cell swelling on deoxy (Fig. 5 *B*) reduces the driving force for calcium influx through PIEZO1 and consequently also the amount of restorative calcium extrusion required by the pump; repolarization on reoxy reverses these changes but only partially, as RCV remains above oxy baseline levels during deoxy-oxy cycling (Fig. 2).

The deoxy-oxy effects on calcium fluxes shown in Fig. 5 *C* cause secondary effects on the amplitude and time course of the cytoplasmic calcium concentrations controlling the periods of Gardos channel activity, as shown in the inset of Fig. 4 *B*. This explains the synchrony between the oscillations in calcium and potassium fluxes shown in Fig. 5, *C* and *D*.

The results in Fig. 5 *E* confirm that the cytoplasmic magnesium buffering properties encoded in the model represent adequately the well-measured changes in [Mg^2+^]_i_ induced by oxy-deoxy transitions (13,14,15). The binding affinity of Hb for ATP and 2,3-DPG is increased by deoxy (52). ATP and 2,3-DPG are the two main Mg^2+^-binding compounds in the RBC cytoplasm (53,54,55,56,57). Deoxy reduces their availability for buffering intracellular magnesium with a consequent increase in [Mg^2+^]_i_. Reoxy rapidly reverses these changes. The model predicts sharp up and down [Mg^2+^]_i_ transitions on deoxy-reoxy transits, with minor volume-related adjustments during the intertransit periods and no detectable influence of PIEZO1-activated processes (Fig. 5 *E*).

Analysis of the results in Fig. 5 leads to the conclusion that oxy-deoxy- and PIEZO1-triggered processes operate additively and independently in the circulation. The minor interactions among homeostatic variables are indirectly generated by membrane-potential-mediated effects.

## Discussion

The main conclusion from the current results is that the rates of volume change following oxy-deoxy transitions are too slow to allow cell volumes to reach steady states in the intervals between capillary transits, forcing continuous volume fluctuations in the circulation throughout the cell’s lifespan (Figs. 1 and 2). The amplitude of the volume displacements varied stochastically with the duration of the intercapillary transits (Fig. 2). Multivariable analysis of the combined changes induced by PIEZO1 and by oxy-deoxy transitions revealed that the two processes operate additively and independently, with minimal interactions mediated by membrane potential oscillations (Figs. 4 and 5). The mechanisms behind the predicted oxy-deoxy- and PIEZO1-induced fluctuations in RBC homeostasis in vivo, analyzed in detail in the results, highlight the extraordinary long-term circulatory balance and stability endowed to the cells by the combination of a low constitutive cation permeability of the plasma membrane and the operation of the Jacob-Stewart cycle. As with hemodynamic motion, RBC homeostasis is never at rest in the circulation. RBC pump-leak balanced steady states, whether in oxy or deoxy conditions, are ex vivo experimental constructs.

Although the dynamic changes in homeostatic variables shown in Figs. 1, 2, 3, and 5 cannot be explored in vivo, future technical developments may eventually enable direct experimental tests of the model predictions. One possible approach would be for RBCs from the same batch loaded with either pH, calcium-sensitive, or membrane potential indicators to circulate in single file through microfluidic channels (58) with intermittent constrictions somehow configured to allow alternative exposure of the cells to O_2_ or to O_2_-free CO_2_ during consecutive constriction passages while monitoring the dynamic fluctuations in the fluorescent signals. Previous results by Swietach et al., using a pH fluorophore (48), and by Danielczok et al. (59), using a calcium indicator in a microfluidic system to detect PIEZO1-triggered signals while traversing constrictions, show that, in principle, this approach is feasible. However, gas control during constriction passages and maintenance of subtoxic light exposure levels while recording dynamic changes from fluorescent signals over extended periods of time pose technical challenges not yet confronted.

The most unexpected prediction encountered during this investigation was that RBCs kept in deoxygenated conditions after an oxy-deoxy transition would slowly reverse their rapid initial volume gain toward a final volume decline far below that of the initial physiological quasi steady state (Fig. 3; Table 1). To understand the mechanism behind this prediction, it was necessary to explore how the values of the main homeostatic variables that drive RBC volume changes differ in between oxy and deoxy stable states. These values are shown in Table 1, together with nHb, the pI-modified variable from which all other downstream changes between oxy and deoxy conditions arise (Eqs. 1 and 2; Fig. 1).

**Table 1:** Predicted differences in the values of selected homeostatic variables in oxy and deoxy steady states of RBCs

Parameter or Variable	RCV	nHb mEq/mol	[Mg^2+^] mM	pH	[Na^+^] mM	[K^+^] mM	[Cl^-^] mM	Em mV
Oxy steady state	1.000	−6.01	0.325	7.22	10.0	140	95.0	−12
Deoxy “physiological” *steady* state	1.041	−3.52	0.708	7.26	9.51	133	107	−8.7
Deoxy final steady state	0.975	−3.33	0.663	7.24	9.40	130	102	−9.9

The values for physiological and final deoxy steady states were taken at 10 min and 20 days, respectively, after the oxy-deoxy transition (Fig. 3).

Table 1 shows that the differences in the concentrations of permeable ions and membrane potentials between the “physiological” deoxy stable state and final deoxy steady state are much smaller than those between oxy and deoxy states. These minute deoxy-state differences point to extremely weak driving gradients for the volume transitions between physiological and final deoxy steady states, thus explaining the exceptional slowness of the predicted transition rates (Fig. 3). The main surprise here was that these same minute differences could ultimately lead to such relatively large RCV differences.

Experimental tests of the predictions in Fig. 3 would require long incubations of deoxygenated RBCs at 37°C and at very low cell volume fractions to ensure the constancy of medium composition, conditions that have been approximated in certain malaria cultures (60,61,62,63,64). It is questionable whether the predicted long-term volume decline of deoxygenated RBCs ever occurs in physiological or pathological conditions in vivo. Therefore, this model prediction ought to be considered of primary academic interest for the time being.

The current study was centered on an RBC modeled with the values of parameters and variables corresponding to the means of measurements performed in blood samples from healthy human adults. In patients with inherited hemolytic anemias associated with RBC hydration disorders, the primary mutations elicit the formation of subpopulations of circulating RBCs with marked differences in volume, intracellular pH, and calcium distributions with patterns specific for each type of anemia (28,52,65,66). In inherited hemoglobinopathies, the abnormal hemoglobins often differ in their proton buffering properties (52,67), the α and pI parameters in the Dalmark equation (1,2). The current modeling study provides the means to investigate the potential effects expected from the abnormal buffering properties of these hemoglobins on the circulatory dynamics of RBCs in these diseases.

Mutant PIEZO1 (22,68,69) and Gardos channels (68,70,71) generate a wide spectrum of hematological abnormalities with varying degrees of RBC hydration disorders, reduced RBC lifespan, anemia, and clinical severity (72). An extension of the RCM used in the current study was recently applied to follow the cumulative effects expected from PIEZO1 activation on capillary entry throughout the nearly 200,000 capillary transits over normal RBC lifespans (19). When applied to investigate the possible effects of deoxy activation of PIEZO1 in sickle cell reticulocytes, the model predicted a rapid hyperdense collapse with a much-reduced RBC lifespan, reminiscent of that documented for irreversibly sickled cells, the cells responsible for microvascular occlusion and organ failures, the major trigger of all downstream effects in sickle cell disease (5,28,73,74).

A common PIEZO1 polymorphism was recently found associated with protection from severe malaria in humans (75), a clear target for a study on the possible mechanism of protection using the model of falciparum-infected RBCs (76). When applied to study the homeostasis of falciparum-infected RBCs in the past (29,61), this model predicted the time course of volume growth of the parasite during its reproduction cycle, a prediction recently verified (60), explained its mechanism, and also showed that digestion of host Hb far in excess of parasites’ biosynthetic needs was needed to prevent premature lysis of the infected cell by reducing the colloid osmotic pressure within the host. These examples demonstrate the potential predictive power of cellular homeostasis models to contribute new insights to the understanding of the mechanisms behind complex cell responses.

Nothing is known at present about how the dynamics of oxy-deoxy-, PIEZO1-, and Gardos channel-dependent RBC responses are affected during capillary transits in any of these pathologies and how these responses contribute to the clinical condition. The modeling approach applied in the present investigation opens the way for in-depth studies on the altered circulatory dynamics that may be expected from RBCs modeled with different constitutive properties or infected with malaria parasites, a vast new area of enquiry for future research.

## Supporting material

Document S1. Supporting material S1 and S2 and Figures S1–S5

Document S2. Article plus supporting material

## Footnotes

Supporting material can be found online at https://doi.org/10.1016/j.bpj.2022.12.038.
