# The circulatory dynamics of human red blood cell homeostasis: Oxy-deoxy and PIEZO1-triggered changes

**DOI:** 10.1016/j.bpj.2025.02.008

**Published:** 2025-02-25

**Authors:** Virgilio L. Lew

## Main text

(Biophysical Journal *122*, 484–495; February 7, 2023)

In the article as originally published, the titles and legends for Figures 4 and 5 were inverted. The titles and legends have been corrected in the online version of the article. The inversion of the titles and legends was present in the author’s accepted manuscript file, and the author apologizes for this error.

