# Supplementary material for: The circulatory dynamics of human red blood cell homeostasis: Oxy-deoxy and PIEZO1-triggered changes
Source: Biophys J. 2022 Dec 31;122(3):484–95. doi: 10.1016/j.bpj.2022.12.038 (PMC9941722; doi:10.1016/j.bpj.2022.12.038)
Supplement: Document S1. Supporting material S1 and S2 and Figures S1–S5 [file mmc1.pdf]

Biophysical Journal, Volume *122*

Supplemental information

The circulatory dynamics of human red blood cell homeostasis: Oxy-deoxy and PIEZO1-triggered changes

Virgilio L. Lew

SM1(supplemental material 1)
Figure protocols
RCM_8560ca5.jar

# Fig 1

RS

DS 1
CVF:0.00001
Accuracy:6
Cyclesperprint(epochs):111
Time:2
FrequencyFactor:0.0001

DS 2
Hb Deoxy or Re-Oxy:Deoxy
Time:10

DS 3
Hb Deoxy or Re-Oxy:Re-Oxy
Time:10

```
# Fig 2.txt
RS
DS 1
CVF:0.00001
Accuracy:4
Cyclesperprint(epochs):111
Time:5
FrequencyFactor:0.001
DS 2
Hb Deoxy or Re-Oxy:Deoxy
Time:10
DS 3
Hb Deoxy or Re-Oxy:Re-Oxy
Time:10
DS 4
Hb Deoxy or Re-Oxy:Deoxy
Time:1.5
DS 5
Hb Deoxy or Re-Oxy:Re-Oxy
Time:1.5
DS 6
Hb Deoxy or Re-Oxy:Deoxy
Time:1.5
DS 7
Hb Deoxy or Re-Oxy:Re-Oxy
Time:1.5
DS 8
Hb Deoxy or Re-Oxy:Deoxy
Time:1.5
DS 9
Hb Deoxy or Re-Oxy:Re-Oxy
Time:1.5
DS 10
Hb Deoxy or Re-Oxy:Deoxy
Time:1.5
DS 11
Hb Deoxy or Re-Oxy:Re-Oxy
Time:1.5
DS 12
Hb Deoxy or Re-Oxy:Deoxy
Time:1.5
DS 13
Hb Deoxy or Re-Oxy:Re-Oxy
Time:1.5
DS 14
Hb Deoxy or Re-Oxy:Deoxy
Time:10
DS 15
Hb Deoxy or Re-Oxy:Re-Oxy
Time:10
DS 16
Hb Deoxy or Re-Oxy:Deoxy
Time:1
DS 17
Hb Deoxy or Re-Oxy:Re-Oxy
Time:1
DS 18
Hb Deoxy or Re-Oxy:Deoxy
```

Time:1
DS 19
Hb Deoxy or Re-Oxy:Re-Oxy
Time:1
DS 20
Hb Deoxy or Re-Oxy:Deoxy
Time:1
DS 21
Hb Deoxy or Re-Oxy:Re-Oxy
Time:1
DS 22
Hb Deoxy or Re-Oxy:Deoxy
Time:1
DS 23
Hb Deoxy or Re-Oxy:Re-Oxy
Time:1
DS 24
Hb Deoxy or Re-Oxy:Deoxy
Time:1
DS 25
Hb Deoxy or Re-Oxy:Re-Oxy
Time:1
DS 26
Hb Deoxy or Re-Oxy:Deoxy
Time:10
DS 27
Hb Deoxy or Re-Oxy:Re-Oxy
Time:10
DS 28
Hb Deoxy or Re-Oxy:Deoxy
Time:0.5
DS 29
Hb Deoxy or Re-Oxy:Re-Oxy
Time:0.5
DS 30
Hb Deoxy or Re-Oxy:Deoxy
Time:0.5
DS 31
Hb Deoxy or Re-Oxy:Re-Oxy
Time:0.5
DS 32
Hb Deoxy or Re-Oxy:Deoxy
Time:0.5
DS 33
Hb Deoxy or Re-Oxy:Re-Oxy
Time:0.5
DS 34
Hb Deoxy or Re-Oxy:Deoxy
Time:0.5
DS 35
Hb Deoxy or Re-Oxy:Re-Oxy
Time:0.5
DS 36
Hb Deoxy or Re-Oxy:Deoxy
Time:0.5
DS 37
Hb Deoxy or Re-Oxy:Re-Oxy
Time:0.5

DS 38
Hb Deoxy or Re-Oxy:Deoxy
Time:10
DS 39
Hb Deoxy or Re-Oxy:Re-Oxy
Time:10
DS 40
Hb Deoxy or Re-Oxy:Deoxy
Time:1.42
DS 41
Hb Deoxy or Re-Oxy:Re-Oxy
Time:1.49
DS 42
Hb Deoxy or Re-Oxy:Deoxy
Time:1.3
DS 43
Hb Deoxy or Re-Oxy:Re-Oxy
Time:1.06
DS 44
Hb Deoxy or Re-Oxy:Deoxy
Time:0.55
DS 45
Hb Deoxy or Re-Oxy:Re-Oxy
Time:1.04
DS 46
Hb Deoxy or Re-Oxy:Deoxy
Time:1.28
DS 47
Hb Deoxy or Re-Oxy:Re-Oxy
Time:0.97
DS 48
Hb Deoxy or Re-Oxy:Deoxy
Time:1.23
DS 49
Hb Deoxy or Re-Oxy:Re-Oxy
Time:1.21
DS 50
Hb Deoxy or Re-Oxy:Deoxy
Time:0.99
DS 51
Hb Deoxy or Re-Oxy:Re-Oxy
Time:1.24
DS 52
Hb Deoxy or Re-Oxy:Deoxy
Time:0.89
DS 53
Hb Deoxy or Re-Oxy:Re-Oxy
Time:1.24
DS 54
Hb Deoxy or Re-Oxy:Deoxy
Time:1.39
DS 55
Hb Deoxy or Re-Oxy:Re-Oxy
Time:1.2
DS 56
Hb Deoxy or Re-Oxy:Deoxy
Time:1.47
DS 57

Hb Deoxy or Re-Oxy:Re-Oxy
Time:1.43
DS 58
Hb Deoxy or Re-Oxy:Deoxy
Time:1.06
DS 59
Hb Deoxy or Re-Oxy:Re-Oxy
Time:0.79
DS 60
Hb Deoxy or Re-Oxy:Deoxy
Time:1.24
DS 61
Hb Deoxy or Re-Oxy:Re-Oxy
Time:1.03
DS 62
Hb Deoxy or Re-Oxy:Deoxy
Time:1.03
DS 63
Hb Deoxy or Re-Oxy:Re-Oxy
Time:1.40
DS 64
Hb Deoxy or Re-Oxy:Deoxy
Time:1.40
DS 65
Hb Deoxy or Re-Oxy:Re-Oxy
Time:0.77
DS 66
Hb Deoxy or Re-Oxy:Deoxy
Time:1.49
DS 67
Hb Deoxy or Re-Oxy:Re-Oxy
Time:0.97
DS 68
Hb Deoxy or Re-Oxy:Deoxy
Time:0.57
DS 69
Hb Deoxy or Re-Oxy:Re-Oxy
Time:0.91
DS 70
Hb Deoxy or Re-Oxy:Deoxy
Time:1.02
DS 71
Hb Deoxy or Re-Oxy:Re-Oxy
Time:0.73
DS 72
Hb Deoxy or Re-Oxy:Deoxy
Time:0.75
DS 73
Hb Deoxy or Re-Oxy:Re-Oxy
Time:1.31
DS 74
Hb Deoxy or Re-Oxy:Deoxy
Time:0.5
DS 75
Hb Deoxy or Re-Oxy:Re-Oxy
Time:0.97
DS 76
Hb Deoxy or Re-Oxy:Deoxy

Time:0.53
DS 77
Hb Deoxy or Re-Oxy:Re-Oxy
Time:0.59
DS 78
Hb Deoxy or Re-Oxy:Deoxy
Time:1.07
DS 79
Hb Deoxy or Re-Oxy:Re-Oxy
Time:1.35
DS 80
Hb Deoxy or Re-Oxy:Deoxy
Time:1.48
DS 81
Hb Deoxy or Re-Oxy:Re-Oxy
Time:1.49
DS 82
Hb Deoxy or Re-Oxy:Deoxy
Time:1.32
DS 83
Hb Deoxy or Re-Oxy:Re-Oxy
Time:1.25
DS 84
Hb Deoxy or Re-Oxy:Deoxy
Time:0.86
DS 85
Hb Deoxy or Re-Oxy:Re-Oxy
Time:1.46
DS 86
Hb Deoxy or Re-Oxy:Deoxy
Time:0.94
DS 87
Hb Deoxy or Re-Oxy:Re-Oxy
Time:0.60
DS 88
Hb Deoxy or Re-Oxy:Deoxy
Time:0.85
DS 89
Hb Deoxy or Re-Oxy:Re-Oxy
Time:1.43
DS 90
Hb Deoxy or Re-Oxy:Deoxy
Time:0.79
DS 91
Hb Deoxy or Re-Oxy:Re-Oxy
Time:0.75
DS 92
Hb Deoxy or Re-Oxy:Deoxy
Time:1.32
DS 93
Hb Deoxy or Re-Oxy:Re-Oxy
Time:0.62
DS 94
Hb Deoxy or Re-Oxy:Deoxy
Time:1.22
DS 95
Hb Deoxy or Re-Oxy:Re-Oxy
Time:0.66

DS 96
Hb Deoxy or Re-Oxy:Deoxy
Time:1
DS 97
Hb Deoxy or Re-Oxy:Re-Oxy
Time:1.04
DS 98
Hb Deoxy or Re-Oxy:Deoxy
Time:1.48
DS 99
Hb Deoxy or Re-Oxy:Re-Oxy
Time:0.60
DS 100
Hb Deoxy or Re-Oxy:Deoxy
Time:0.67
DS 101
Hb Deoxy or Re-Oxy:Re-Oxy
Time:1.44
DS 102
Hb Deoxy or Re-Oxy:Deoxy
Time:1.38
DS 103
Hb Deoxy or Re-Oxy:Re-Oxy
Time:1.02
DS 104
Hb Deoxy or Re-Oxy:Deoxy
Time:0.78
DS 105
Hb Deoxy or Re-Oxy:Re-Oxy
Time:0.89
DS 106
Hb Deoxy or Re-Oxy:Deoxy
Time:1.06
DS 107
Hb Deoxy or Re-Oxy:Re-Oxy
Time:0.56
DS 108
Hb Deoxy or Re-Oxy:Deoxy
Time:0.64
DS 109
Hb Deoxy or Re-Oxy:Re-Oxy
Time:1.17
DS 110
Hb Deoxy or Re-Oxy:Deoxy
Time:1.40
DS 111
Hb Deoxy or Re-Oxy:Re-Oxy
Time:1.03
DS 112
Hb Deoxy or Re-Oxy:Deoxy
Time:0.75
DS 113
Hb Deoxy or Re-Oxy:Re-Oxy
Time:1.26
DS 114
Hb Deoxy or Re-Oxy:Deoxy
Time:0.57
DS 115

Hb Deoxy or Re-Oxy:Re-Oxy
Time:0.93
DS 116
Hb Deoxy or Re-Oxy:Deoxy
Time:0.83
DS 117
Hb Deoxy or Re-Oxy:Re-Oxy
Time:1.35
DS 118
Hb Deoxy or Re-Oxy:Deoxy
Time:0.56
DS 119
Hb Deoxy or Re-Oxy:Re-Oxy
Time:0.80
DS 120
Hb Deoxy or Re-Oxy:Deoxy
Time:0.78
DS 121
Hb Deoxy or Re-Oxy:Re-Oxy
Time:1.28
DS 122
Hb Deoxy or Re-Oxy:Deoxy
Time:0.61
DS 123
Hb Deoxy or Re-Oxy:Re-Oxy
Time:0.56
DS 124
Hb Deoxy or Re-Oxy:Deoxy
Time:1.45
DS 125
Hb Deoxy or Re-Oxy:Re-Oxy
Time:0.66
DS 126
Hb Deoxy or Re-Oxy:Deoxy
Time:1.44
DS 127
Hb Deoxy or Re-Oxy:Re-Oxy
Time:0.84
DS 128
Hb Deoxy or Re-Oxy:Deoxy
Time:1.43
DS 129
Hb Deoxy or Re-Oxy:Re-Oxy
Time:0.70
DS 130
Hb Deoxy or Re-Oxy:Deoxy
Time:1.25
DS 131
Hb Deoxy or Re-Oxy:Re-Oxy
Time:0.62
DS 132
Hb Deoxy or Re-Oxy:Deoxy
Time:1.48
DS 133
Hb Deoxy or Re-Oxy:Re-Oxy
Time:1.1
DS 134
Hb Deoxy or Re-Oxy:Deoxy

Time:1.26
DS 135
Hb Deoxy or Re-Oxy:Re-Oxy
Time:0.6
DS 136
Hb Deoxy or Re-Oxy:Deoxy
Time:1.43
DS 137
Hb Deoxy or Re-Oxy:Re-Oxy
Time:1.14
DS 138
Hb Deoxy or Re-Oxy:Deoxy
Time:0.79
DS 139
Hb Deoxy or Re-Oxy:Re-Oxy
Time:1.38
DS 140
Hb Deoxy or Re-Oxy:Deoxy
Time:10
DS 141
Hb Deoxy or Re-Oxy:Re-Oxy
Time:10

# Fig 3

RS

DS 1
CVF:0.00001
Cyclesperprint(epochs):11
Time:1440

DS 2
Accuracy:6
Cyclesperprint(epochs):777
Hb Deoxy or Re-Oxy:Deoxy
Time:30240

DS 3
Hb Deoxy or Re-Oxy:Re-Oxy
Time:30240

DS 4
Hb Deoxy or Re-Oxy:Deoxy
Time:30240

Fig 4

RS

DS 1
CVF:0.00001
Cyclesperprint(epochs):11
Time:2
FrequencyFactor:0.0001

DS 2
Restore Medium (no/yes):no
Pz stage no or yes:yes
Accuracy:6
Pz transit CVF:0.00001
PzA:50
Pzcyclesperprint:11
Time:0.007
PzFrequencyFactor:0.00001
PzCa:70

DS 3
Cyclesperprint(epochs):77
Time:5
FrequencyFactor:0.00001

```
# Fig 5

RS

DS 1
CVF:0.00001
Cyclesperprint(epochs):111
Time:2
FrequencyFactor:0.0001

DS 2
Restore Medium (no/yes):no
Pz stage no or yes:yes
Pz transit CVF:0.00001
PzA:50
Time:0.007
PzFrequencyFactor:0.001
PzCa:70

DS 3
Time:1

DS 4
Restore Medium (no/yes):no
Pz stage no or yes:yes
Pz transit CVF:0.00001
PzA:50
Time:0.007
PzFrequencyFactor:0.001
PzCa:70

DS 5
Time:1

DS 6
Restore Medium (no/yes):no
Pz stage no or yes:yes
Pz transit CVF:0.00001
PzA:50
Hb Deoxy or Re-Oxy:Deoxy
Time:0.007
PzFrequencyFactor:0.001
PzCa:70

DS 7
Time:1

DS 8
Restore Medium (no/yes):no
Pz stage no or yes:yes
Pz transit CVF:0.00001
PzA:50
Hb Deoxy or Re-Oxy:Re-Oxy
Time:0.007
PzFrequencyFactor:0.001
PzCa:70

DS 9
```

Time:1

DS 10
Restore Medium (no/yes):no
Pz stage no or yes:yes
Pz transit CVF:0.00001
PzA:50
Hb Deoxy or Re-Oxy:Deoxy
Time:0.007
PzFrequencyFactor:0.001
PzCa:70

DS 11
Time:1

DS 12
Restore Medium (no/yes):no
Pz stage no or yes:yes
Pz transit CVF:0.00001
PzA:50
Hb Deoxy or Re-Oxy:Re-Oxy
Time:0.007
PzFrequencyFactor:0.001
PzCa:70

DS 13
Time:1

DS 14
Restore Medium (no/yes):no
Pz stage no or yes:yes
Pz transit CVF:0.00001
PzA:50
Hb Deoxy or Re-Oxy:Deoxy
Time:0.007
PzFrequencyFactor:0.001
PzCa:70

DS 15
Time:1

DS 16
Restore Medium (no/yes):no
Pz stage no or yes:yes
Pz transit CVF:0.00001
PzA:50
Hb Deoxy or Re-Oxy:Re-Oxy
Time:0.007
PzFrequencyFactor:0.001
PzCa:70

DS 17
Time:1

DS 18
Restore Medium (no/yes):no
Pz stage no or yes:yes
Pz transit CVF:0.00001

```
PzA:50
Hb Deoxy or Re-Oxy:Deoxy
Time:0.007
PzFrequencyFactor:0.001
PzCa:70

DS 19
Time:1

DS 20
Restore Medium (no/yes):no
Pz stage no or yes:yes
Pz transit CVF:0.00001
PzA:50
Hb Deoxy or Re-Oxy:Re-Oxy
Time:0.007
PzFrequencyFactor:0.001
PzCa:70

DS 21
Time:1

DS 22
Restore Medium (no/yes):no
Pz stage no or yes:yes
Pz transit CVF:0.00001
PzA:50
Hb Deoxy or Re-Oxy:Deoxy
Time:0.007
PzFrequencyFactor:0.001
PzCa:70

DS 23
Time:1

DS 24
Restore Medium (no/yes):no
Pz stage no or yes:yes
Pz transit CVF:0.00001
PzA:50
Hb Deoxy or Re-Oxy:Re-Oxy
Time:0.007
PzFrequencyFactor:0.001
PzCa:70

DS 25
Time:1

DS 26
Restore Medium (no/yes):no
Pz stage no or yes:yes
Pz transit CVF:0.00001
PzA:50
Hb Deoxy or Re-Oxy:Deoxy
Time:0.007
PzFrequencyFactor:0.001
PzCa:70
```

DS 27
Time:1

DS 28
Restore Medium (no/yes):no
Pz stage no or yes:yes
Pz transit CVF:0.00001
PzA:50
Hb Deoxy or Re-Oxy:Re-Oxy
Time:0.007
PzFrequencyFactor:0.001
PzCa:70

DS 29
Time:1

DS 30
Restore Medium (no/yes):no
Pz stage no or yes:yes
Pz transit CVF:0.00001
PzA:50
Hb Deoxy or Re-Oxy:Deoxy
Time:0.007
PzFrequencyFactor:0.001
PzCa:70

DS 31
Time:1

DS 32
Restore Medium (no/yes):no
Pz stage no or yes:yes
Pz transit CVF:0.00001
PzA:50
Hb Deoxy or Re-Oxy:Re-Oxy
Time:0.007
PzFrequencyFactor:0.001
PzCa:70

DS 33
Time:1

DS 34
Restore Medium (no/yes):no
Pz stage no or yes:yes
Pz transit CVF:0.00001
PzA:50
Hb Deoxy or Re-Oxy:Deoxy
Time:0.007
PzFrequencyFactor:0.001
PzCa:70

DS 35
Time:1

DS 36
Restore Medium (no/yes):no
Pz stage no or yes:yes

Pz transit CVF:0.00001
PzA:50
Hb Deoxy or Re-Oxy:Re-Oxy
Time:0.007
PzFrequencyFactor:0.001
PzCa:70

DS 37
Time:1

DS 38
Restore Medium (no/yes):no
Pz stage no or yes:yes
Pz transit CVF:0.00001
PzA:50
Time:0.007
PzFrequencyFactor:0.001
PzCa:70

DS 39
Time:1

DS 40
Restore Medium (no/yes):no
Pz stage no or yes:yes
Pz transit CVF:0.00001
PzA:50
Time:0.007
PzFrequencyFactor:0.001
PzCa:70

DS 41
Time:1

DS 42
Restore Medium (no/yes):no
Pz stage no or yes:yes
Pz transit CVF:0.00001
PzA:50
Time:0.007
PzFrequencyFactor:0.001
PzCa:70

DS 43
Time:1

DS 44
Restore Medium (no/yes):no
Pz stage no or yes:yes
Pz transit CVF:0.00001
PzA:50
Time:0.007
PzFrequencyFactor:0.001
PzCa:70

DS 45
Time:1

DS 46
Restore Medium (no/yes):no
Pz stage no or yes:yes
Pz transit CVF:0.00001
PzA:50
Time:0.007
PzFrequencyFactor:0.001
PzCa:70

DS 47
Time:5

SM2 (supplemental material 2)

The governing equations of the red blood cell model (RCM)

Virgilio L. Lew

Cambridge, March 2022 update

1 Introduction

Red blood cell homeostasis addresses the subset of mechanisms that control the dynamic changes in cell volume, membrane potential, ionic composition, membrane transport and osmotic gradients in response to perturbations. The modelled system consists of a suspension of identical RBCs whose dynamic behaviour is constrained only by charge and mass conservation. The equations implement these laws following a strict computational sequence representative of the multiple interconnected processes involved. The default values set for all the model parameters have been experimentally determined, allowing model outputs to predict the homeostatic behaviour of human RBCs in physiological, pathological and experimental conditions within accuracy margins of about 5-10%.

The current text of the Governing Equations of the RCM is an updated version of the original published in 2021 [1, 2]. The complete model code, together with a User Guide and a detailed tutorial, are available with open access in the repository (https://github.com/sdrogers/redcellmodeljava)

1.1 The initial Reference State (RS)

The RBC reference state describes the initial condition of the system in a pump-leak balanced steady state. For compliance with initial electroneutrality and osmotic equilibrium we use a phenomenology in which the charge, nX or n_X, and cell content of the global, non-haemoglobin, impermeant cell anion, QX^-, are treated as wildcard parameters in equations 3 and 8. The n_x and CX^- values emerging from such treatment correspond closely with the known organic and inorganic phosphate pools of metabolically normal RBCs [3]. When modifying the initial default values in the RS the wildcard parameters may change. The model automatically recalculates their value potentially changing slightly the constitutive make up of the impermeant cell anion ($n_X QX^-$) in the new cell.

1.1.1 Medium electroneutrality

$$MA + (MB - MBH) + M_{gluconate} - (MNa + MK + 2(MCa^{2+} + MMg^{2+}) + M_{glucamine}) = 0 \tag{1}$$

Medium concentration of proton-bound buffer, MBH (HEPES, by default):

$$MBH = MB\left(\frac{MH}{K_B + MH}\right) \tag{2}$$

1.1.2 Intracellular electroneutrality:

$$CNa + CK + CH + 2CMg^{2+} + 2CCa^{2+} - (CA + n_{Hb}CHb + n_X CX^-) = 0 \tag{3}$$

n_{Hb}, the net charge on the haemoglobin molecule, is represented by the Cass-Dalmark equation [4],

$$n_{Hb} = \alpha(pH_i - pI) \tag{4}$$

where α corresponds to the linear segment of the proton titration curve of Hb in intact RBCs, and pI is the pH_i at the isoelectric point of haemoglobin.

In the Reference steady state the net fluxes of each of the i-transported solutes is zero, and pump-leak balance is represented by $\sum I_j = F\sum z_j F_j = 0$, where I_j is the current carried by transporter j, F is the Faraday constant, z_j is the net charge on each of the j-transporters, and F_j is the net flux through the j-transporter; z $\neq$ 0 only for electrogenic transporters.

1.1.3 Medium and cell osmolarities, MOs and COs

$$MOs = MA + MB + M_{gluconate} + MNa + MK + MCat + MMgt + M_{glucamine} \tag{5}$$
$$COs = CNa + CK + CA + CH + CMg^{2+} + CCa^{2+} + f_{Hb}CHb + CX^{-} \tag{6}$$

f_{Hb} is the osmotic coefficient of haemoglobin, represented with only two virial coefficients, b and c:

$$f_{Hb} = 1 + b \times CHb + c \times CHb^2 \tag{7}$$

1.1.4 Osmotic equilibrium in the reference steady state:

$$MOs = COs \tag{8}$$

1.1.5 Cytoplasmic buffering of protons, calcium and magnesium.

Heamoglobin is the major cytoplasmic buffer for protons (eq 4) and for calcium (α-buffer in eq 9c). The main magnesium buffers are ATP and 2,3-DPG, compounds integrated within the X- phenomenology. Because the bound forms of Ca and Mg are contained within CX^{-}, they are not included as separate osmolarity contributors in eq 6, leaving only the free forms of Ca^{2+} and Mg^{2+} as osmotic contributors.

Cytoplamic Ca^{2+} and Mg^{2+} buffering have been measured with precision in intact RBCs [5, 6, 7, 8] enabling accurate representations in the model. The total Ca and Mg content of the cells, QCa and QMg, is reported in units of mmol/(340g Hb) (or mmol/Loc) whereas concentrations of the free forms, CCa^{2+} and CMg^{2+}, are expressed in units of mmol/Lcw, a conversion requiring translation for operational reasons in the model. Equation 9a translates QCa in units of mmol/Loc to CCa in units of mmol/Lcw using:

$$CCa = QCa\left(\frac{RCV}{V\omega}\right) \tag{9a}$$

The total calcium concentration is the sum of free and bound forms:

$$CCa = CCa^{2+} + CCaB \tag{9b}$$

There are two buffer systems for binding calcium in the RBC cytoplasm, α (mostly haemoglobin), and the BCa/KBCa buffer [6]. The concentration of bound calcium, CCaB, at each total calcium concentration, CCa, is represented by:

$$CCaB = \alpha CCa + CBCa\left(\frac{CCa^{2+}}{CCa^{2+} + K_{BC_a}}\right) \tag{9c}$$

CCa^{2+} is solved from the implicit equation:

$$CCa - CCa^{2+} - CCaB = 0 \tag{9d}$$

by the Newton-Raphson routine in the RS and at the end of the computations in each iteration cycle. The measured values of the calcium binding parameters are α =0.30, C_{BC_a} =0.026 mmol/Loc, and K_{BC_a} =0.014 mM [6].

The corresponding equations for cytoplasmic magnesium buffering and CMg^{2+} are:

$$CMg = QMg\left(\frac{RCV}{V\omega}\right) \tag{9e}$$
$$CMg = CMg^{2+} + CMgB \tag{9f}$$
$$CMgB = CB_{Mg}1\left(\frac{CMg^{2+}}{CMg^{2+} + KB_{Mg}1}\right) + CB_{Mg}2\left(\frac{CMg^{2+}}{CMg^{2+} + KB_{Mg}2}\right) + CB_{Mg}3 \tag{9g}$$

CMg^{2+} is solved from the implicit equation:

$$CMg - CMg^{2+} - CMgB = 0 \tag{9h}$$

The measured values of the Mg bufferes [8] are: $CB_{Mg}1$ =1.2 mmol/Loc, $KB_{Mg}1$ =0.08 mM; $CB_{Mg}2$ =7.5 mmol/Loc (15 mEq/Loc), $KB_{Mg}2$ =3.6 mM; $CB_{Mg}3$ =0.05 mmol/Loc. $B_{Mg}1$ represents ATP, $B_{Mg}2$ represents 2,3-DPG and miscellaneous phosphate groups, and $B_{Mg}3$ is an unidentified high affinity magnesium buffer.

1.1.6 Effects of deoxygenation on cytoplasmic Mg^{2+} buffering and pHi.

Deoxygenation increases haemoglobin binding of ATP and 2,3-DPG thus reducing their availability for buffering intracellular magnesium. $CB_{Mg}1$ is reduced by half and $CB_{Mg}2$ by 1.7 [8]. This is particularly relevant for simulating accurately the effects of changing the oxygenation condition of RBCs, a process in which changes in CMg^{2+} become enmeshed with effects arising from changes in the isoelectric point of haemoglobin, pI (eq 4).

1.1.7 Charge balance during Mg^{2+} buffering changes.

By treating the Mg buffers as part of the global impermeant cell ion the osmotic balance on deoxygenation remains undisturbed. On the other hand, the change in Mg^{2+} charge, QMg^{2+}, needs balancing from within the global charge on X, $nXQX$ by a corresponding change in nX. Because X and Mg are treated as impermeant cytoplasmic solutes in the model, their amounts per litre original cells, QX and QMg, are set in the Reference State as constants in the system, their concentrations varying only with changes in cell volume. Therefore, from $\Delta nX = nX_{deoxy} - nX_{oxy}$ and from $QX^-_{deoxy} - QX^-_{oxy} = 2(QMg^{2+}_{deoxy} - QMg^{2+}_{oxy})$, we derive $nX_{deoxy} = nX_{oxy} - 2(QMg^{2+}_{deoxy} - QMg^{2+}_{oxy})/QX$. Thus, both nX_{oxy} and nX_{deoxy} become defined as constants whose values are set by the values of QMg^{2+} and of the wildcard parameters nX and QX^- as derived from equations 3 and 8 in the Reference State. With the current default settings in the Reference State, nX_{oxy} =-0.4352, and nX_{deoxy} =-0.4511, values instantly changed and reversed during deoxygenation-reoxygenation cycles.

1.1.8 Effects of change in the isoelectric point of haemoglobin on cell pH, pHi.

Hb is assumed to be in a oxy-state by default, the most frequent experimental condition. Deoxygenation of Hb (Deoxy) changes its pI(0degC) from 7.2 to 7.5. The model automatically adjusts the actual pI change for the temperature of the experiment. The pI shifts during oxy-deoxy transitions cause sudden changes in the protonization condition of Hb with secondary changes in pHi, changes which the model predicts with verified accuracy [9, 10, 11]. Electroneutrality preservation during oxy-deoxy transitions requires constancy of nHb values (eq 4) when pI changes, from which the compensatory changes in pHi can be derived according to [10]:

On deoxygenation:

$$pHi_{deoxy} = pHi_{oxy} + pI_{deoxy} - pI_{oxy}$$

On reoxygenation:

$$pHi_{oxy} = pHi_{deoxy} + pI_{oxy} - pI_{deoxy}$$

1.2 The Dynamic State.

A first requirement at the start of simulations is to define the relative volume occupied by cells in the cell suspension system, the cell volume fraction, CVF. Perturbations alter the flux of transported solutes and water across the plasma membrane of the cell thus initiating a cascade of downstream changes in the compositions of cell and suspending medium. It is therefore important to start by listing the membrane transport component of the cell and of the equations describing their basic kinetic properties.

1.2.1 Flux equations of the model, Fi and Fj

The substrates of the RBC membrane transporters are Na, K, A, H, Ca, Mg and water, the "i" in Fi. The sign-convention applied in the equations is for positive net fluxes into the cell (influx) and for negative net fluxes into the medium (efflux). The name convention adopted here for the transport of substrate X by the different membrane transporters is as follows: FPX=pump-mediated flux of X, with P=NaP for the Na/K pump or CaP for the calcium pump (PMCA); FGX=X-flux through electrodiffusional channel defined with constant field kinetics; FXA=electroneutral carrier-mediated cotransport of cation X and anion A defined with low-saturation kinetics; FzX=electrodiffusional flux

of X through PIEZO1 channel; FCoX=electroneutral cotransport of X mediated by the Na:K:2Cl symport, of minimal expression and activity in human RBCs; FA23X=electroneutral $M^{2+}:2H^{+}$ exchange flux through the divalent cation ionophore A23187, the only exogenous membrane transporter included in the model; Fw=water flux mediated mainly by aquaporins and partly by partition diffusion through the plasma membrane.

1.2.2 Flux pathways for each transported substrate, Fi:

$$FNa = F_{NaP}Na + FGNa + FNaA + F_{Co}Na + FzNa \tag{10a}$$
$$FK = F_{NaP}K + F_{G}K + FKA + FK_{Gardos} + F_{Co}K + FzK \tag{10b}$$
$$FA = F_{G}A + FHA + FNaA + FKA + FzA + 2FCoA \tag{10c}$$
$$FH = F_{G}H + FHA + F_{CaP}H + F_{A23}H \tag{10d}$$
$$FCa = F_{CaP}Ca + F_{G}Ca + FzCa + F_{A23}Ca \tag{10e}$$
$$FMg = F_{A23}Mg \tag{10f}$$
$$Fw = Pw(COs - MOs) \tag{10g}$$

There are no data on PIEZO1-mediated Mg^{2+} fluxes in RBCs. Although PzMg most certainly has a small finite value, FzMg is likely to be very small under the usually low electrochemical Mg^{2+} gradients across the RBC membrane. With this level of uncertainty, FzMg was not included in the current model version.

1.2.3 Kinetic descriptions of individual transporters

Certain transporter kinetics are reported in the equations with the default numerical values used for dissociation and rate constants in the model, based on well established values in the literature and on the good semi-quantitative fits to experimental data provided in the past [12, 13, 14].

1.2.4 Na/K pump mediated fluxes of Na and K (f=forward; r=reverse) [15, 16]

$$F_{NaP}Na^{f} = -F_{NaP}max^{f}\left(\frac{CNa}{CNa + 0.2(1 + CK/8.3)}\right)^{3}\left(\frac{MK}{MK + 0.1(1 + MNa/18)}\right)^{2} \tag{11a}$$
$$F_{NaP}Na^{r} = F_{NaP}max^{r}\left(\frac{CK}{CK + 8.3(1 + CNa/0.2)}\right)^{2}\left(\frac{MNa}{MNa + 18(1 + MK/0.1)}\right)^{3} \tag{11b}$$
$$F_{NaP}Na = F_{NaP}Naf + F_{NaP}Nar \tag{11c}$$
$$F_{NaP}K = -F_{NaP}Na/1.5 \tag{11d}$$

1.2.5 PMCA. Calcium and proton fluxes through the calcium pump operating as an electroneutral Ca:2H exchanger [17, 18]

$$F_{CaP}Ca = -kCaP\left(\frac{(CCa^{2+})^{4}}{(0.0002)^{4} + (CCa^{2+})^{4}}\right) \tag{12a}$$
$$F_{CaP}H = -2 \times F_{CaP}Ca \tag{12b}$$

1.2.6 Electrodiffusional fluxes of i (Na, K, Ca, H and A) through endogenous channels, FGi , Gardos channels, FGGardos, and PIEZO1 channels, Fzi, are represented with constant field kinetics [19]:

$$F_{G}i = -P_{G}i\left(\frac{ziFEm}{RT}\right)\left(\frac{Ci - Mi\exp^{-ziFEm/RT}}{1 - exp(-ziFEm/RT)}\right) \tag{13}$$

with PGi representing the Goldmanian i-permeability in h^{-1} units

1.2.7 PGKGardos is a function of CCa^{2+} [20, 21] as follows:

$$P_{G}K_{Gardos} = PK_{GardosMax}\left(\frac{(CCa^{2+})^{4}}{(K_{Ca})^{4} + (CCa^{2+})^{4}}\right) \tag{14}$$

1.2.8 PGCa is a function of CCa^{2+} and MCa^{2+} [21, 22] as follows:

$$P_GCa = \left(\frac{CCa^{2+}}{0.0002 + CCa^{2+}}\right)\left(\frac{MCa^{2+}}{0.8 + MCa^{2+}}\right) \tag{15}$$

1.2.9 Low-saturation, carrier mediated flux phenomenology for electroneutral cotransporters FNaA, FKA and FHA.

$$F_{Na}A = -k_{NaA}(CNa \times CA - MNa \times MA) \tag{16a}$$
$$F_KA = -k_{KA}(CK \times CA - MK \times MA) \tag{16b}$$
$$F_HA = -k_{HA}(CH \times CA - MH \times MA) \tag{16c}$$

Note that k_{HA}, the rate constant of the H:A cotransport phenomenology representing the operation of the Jacob-Stewart mechanism (JS) is between five and six orders of magnitude faster than that of any of the other ion transporters in the membrane (see User Guide for details and references).

1.2.10 Electroneutral Na:K:2A cotransport

$$F_{Co} = -k_{Co}((CNa \times CK \times CA^2) - d(MNa \times MK \times MA^2)) \tag{17a}$$
$$d = \frac{CNa \times CK \times CA^2}{MNa \times MK \times MA^2} \tag{17b}$$

The CX and MX values in eq 17b are those set for the RS

$$FNa_{Co} = FK_{Co} = F_{Co} \tag{17c}$$
$$FA_{Co} = 2F_{Co} \tag{17d}$$

d is a wildcard factor introduced to set $F_{Co} = 0$ only in the RS. Its value is set by the initial Na, K and A concentrations in the RS. d remains as a fixed-value parameter during dynamic state computations.

1.2.11 Electroneutral $M^{2+} : 2^{H+}$ exchange fluxes of Ca^{2+} and Mg^{2+} mediated by the divalent cation ionophore A23187

The divalent cation ionophore A23187 mediates an electroneutral $M^{2+} : 2^{H+}$ exchange when incorporated into cell membranes [23]. Divalent cation ionophores became essential and extensively used tools in research on calcium and magnesium function and dysfunction in RBCs [5, 7, 24, 25] and in many other cell types. To emulate experimental protocols with the use of divalent cation ionophores it became necessary to represent their transport properties in the model as an optional exogenous transporter of the RBC membrane.

In albumin-free RBC suspensions, the RBC/medium partition ratio of the lipophilic ionophore A23187 is 60/1, 20 to 50% of it confined to the cell membrane [26]. The transport kinetics of the ionophore was modeled with symmetric binding (Km) and inhibitory (KI) dissociation constants for Ca^{2+} and Mg^{2+} on each membrane side, as follows:

$$A1 = \frac{MCa^{2+}}{Km_{Ca}(1 + MMg^{2+}/(KIMg + MCa^{2+}))}$$
$$A2 = \frac{CCa^{2+}}{Km_{Ca}(1 + CMg^{2+}/(KIMg + CCa^{2+}))}$$
$$A3 = \frac{MMg^{2+}}{Km_{Mg}(1 + MCa^{2+}/(KICa + MMg^{2+}))}$$
$$A4 = \frac{CMg^{2+}}{Km_{Mg}(1 + CCa^{2+}/(KICa + CMg^{2+}))}$$

Following extensive preliminary tests [27], default values of 10 mM for the four Km and KI parameter set were found to deliver excellent agreement between predicted and measured ionophore-mediated fluxes, and to ensure adequate compliance with the measured equilibrium distribution of the transported ions when ionophore-mediated net fluxes approach zero [23]:

$$CCa^{2+}/MCa^{2+} \approx CMg^{2+}/MMg^{2+} \approx (CH+/MH+)^2.$$

Combining the Ca^{2+}, Mg^{2+} and H^+ driving gradients we obtain:

$$B1 = A1(CH)^2 - A2(MH)^2$$
$$B2 = A3(CH)^2 - A4(MH)^2$$

The ionophore-mediated fluxes of Ca^{2+}, Mg^{2+} and H^+, $F_{A23}Ca$, $F_{A23}Mg$ and $F_{A23}H$, respectively, can now be computed from:

$$F_{A23}Ca = P_{A23}B1 \quad \text{(A23-1)}$$
$$F_{A23}Mg = P_{A23}B2 \quad \text{(A23-2)}$$
$$F_{A23}H = -2(F_{A23}Ca + F_{A23}Mg) \quad \text{(A23-3)}$$

Where P_{A23} is the ionophore-mediated permeability. P_{A23} is a power function of the RBC ionophore concentration, $P_{A23} = 0.22[I]^{1.45}$, when P_{A23} is expressed in units of 10^{-6} cm/s, and [I] in μmol/Loc [26, 28, 29]. Within the units-set in the model, numerical values of P_{A23} in the range 1017 to 21018 offered a perfectly adequate minimalist emulation of the effects of different ionophore concentrations on the fluxes and distributions of Ca^{2+}, Mg^{2+} and H^+ ions in RBCs in a large variety of experimental conditions [5, 27, 30, 31, 32, 33].

1.2.12 Equation sequence for the computations of dynamic states.

Following perturbations, sustained charge conservation and electroneutrality is implemented by:

$$\sum I_j = 0 \quad \text{(18a)}$$

where I_j represents the current carried by each of the j-membrane transporters. $\sum I_j$ is therefore the fist equation that has to be solved at the start of each iteration in the computational sequence of dynamic states. Capacitative currents ($Ic = C(dV/dt)$ are ignored because their magnitude and time-course are orders of magnitude smaller than those of the homeostatic relevant currents. The relation between currents and fluxes, F_j, for each transporter is given by

$$I_j = F z_j F_j \quad \text{(18b)}$$

With the electrogenic flux components in the model ($z_j \neq 0$), $\sum I_j = 0$ renders:

$$\sum I_j = F_{NaP}Na + F_{NaP}K + F_GNa + F_GK + F_GK_{Gardos} + F_GA + F_GCa + F_GH + F_zNa + F_zK + F_zA + F_zCa = 0 \quad \text{(18c)}$$

$\sum I_j$ is a complex function of temperature, membrane potential, Em, and of the concentration of all transported and modulating substrates. With all parameters, kinetics and substrate concentrations known $\sum I_j = 0$ becomes an implicit equation in Em, the single unknown left, solved in each iteration with the Newton-Raphson cord approximation routine.

With Em, the new z_jF_j values for each of the electrodiffusional terms in eq 18c can be computed. We can now add up the absolute values of the new computed fluxes to the values of the electroneutral fluxes in the previous iteration, $\sum |F_j|$, to assign a new Δt duration to each current iteration interval, as follows:

$$\Delta t = \frac{a}{b + \sum |F_j|} \quad \text{(19)}$$

The value of a, under user control, optimises Δt scales for different simulations (“frequencyfactor” in the RCM); b is a small zero-avoidance parameter in the denominator. The advantage of this strategy over using regular iteration intervals is that by setting a constant value for the cycles per outcome (“cyclesperprint(epochs)” in the RCM) the density of data output points automatically adjusts to the overall rate of change in the system, emulating the way good experimental practice seeks to sample for data at the bench, thus optimizing comparisons between predicted and experimental results.

With the new F_i^t and Δt the new Q_i^t may be computed using the values of F_{Na}^t, F_K^t, F_A^t, F_H^t, F_{Ca}^t and $F_{A23}Mg^t$ from equations (10a-f) as follows:

$$\Delta QNa = FNa \times \Delta t \tag{20a}$$
$$\Delta QK = FK \times \Delta t \tag{20b}$$
$$\Delta QA = FA \times \Delta t \tag{20c}$$
$$\Delta H = FH \times \Delta t \tag{20d}$$
$$\Delta QCa = FCa \times \Delta t \tag{20e}$$
$$\Delta QMg = F_{A23}Mg \times \Delta t \tag{20f}$$
$$QNa^t = QNa^{(t-\Delta t)} + \Delta QNa \tag{20g}$$
$$QK^t = QK^{(t-\Delta t)} + \Delta QK \tag{20h}$$
$$QA^t = QA^{(t-\Delta t)} + \Delta QA \tag{20j}$$
$$QCa^t = QCa^{(t-\Delta t)} + \Delta QCa \tag{20k}$$
$$QMg^t = QMg^{(t-\Delta t)} + \Delta QMg \tag{20l}$$

ΔH is a special case because ΔH adds to the only titratable proton buffer $n_{Hb} \times QHb$, so that:

$$n_{Hb}^t QHb = n_{Hb}^{(t-\Delta t)} \times QHb + \Delta H \tag{21a}$$
$$n_{Hb}^t = n_{Hb}^{(t-\Delta t)} + \frac{\Delta H}{QHb} \tag{21b}$$

From which we can now compute the new cell pHi from eq 4 by solving for pH^t:

$$pH^t = \frac{n_{Hb}^t}{\alpha} + pI \tag{21c}$$

The new intracellular H^+ concentration in molar units is:

$$CH^t = 10^{-pH^t} \tag{21d}$$

With the new Qi^t, we need the new cell water volume, $V\omega^t$ in order to compute the new cell concentrations, $Ci^t = Qi^t/V\omega^t$. The water flux across the RBC membrane, $F\omega$, is driven by the osmotic gradient across the RBC membrane (eqs 5 and 6):

$$F\omega^t = P\omega(COs^t - MOs^{(t-\Delta t)}) \tag{22a}$$

COs^t can be computed from the altered osmotic load resulting from the ΔQ_i changes during Δt operating on the cell volume at the start of the each iteration interval:

$$COs^t = \frac{QNa^t + QK^t + QA^t + QCa^t + QMg^t}{V\omega^{(t-\Delta t)}} + (f_{Hb} \times CHb + CX)^{(t-\Delta t)} \tag{22b}$$

The new cell water volume, $V\omega^t$, and volume-associated variables, RCV^t, $MCHC^t$, $Density^t$ and Hct^t, can now be computed from:

$$\Delta V\omega^t = F\omega^t \Delta t \tag{23a}$$
$$V\omega^t = V\omega^{(t-\Delta t)} + \Delta V\omega \tag{23b}$$
$$RCV^t = 1 - V\omega^{(t=0)} + V\omega^t \tag{23c}$$
$$MCHC^t = MCHC^{(t=0)}/RCV \tag{23d}$$
$$Density^t = ((MCHC^{(t=0)}/100) + V\omega^t)/RCV \tag{23e}$$
$$Hct^t = Htc^{(t=0)} RCV \tag{23f}$$

With $V\omega^t$ we proceed to compute next the new ^{t} intracellular concentrations of Na, K, A, H, Ca, Hb, and X^-:

$$CNa^t = QNa^t/V\omega^t \quad (24a)$$
$$CK^t = QK^t/V\omega^t \quad (24b)$$
$$CA^t = QA^t/V\omega^t \quad (24c)$$
$$CCa^t = QCa^t/V\omega^t \quad (24d)$$
$$CMg^t = QMg^t/V\omega^t \quad (24e)$$
$$CHb^t = QHb/V\omega^t \quad (24f)$$
$$(CX^-)^t = QX^-/V\omega^t \quad (24g)$$

The new osmotic coefficient of Hb, f^t_{Hb}, can now be calculated from eq 7 and the new CHb^t:

$$f^t_{Hb} = 1 + b \times CHb^t + c \times (CHb^t)^2 \quad (25)$$

1.2.13 Computation of the medium concentrations at time=t.

Medium concentration changes arise from independent solute and water transfers between cells and medium under mass conservation. At constant suspension volume, water transfers between cells and medium generate self-compensating changes in cell and medium volume fractions, CVF and (1-CVF), respectively, according to:

$$\Delta CVF + \Delta(1 - CVF) = 0 \quad (26a)$$

By mass conservation, the Qi changes during Δt, ΔQi, are transferred to the medium, ΔQim, so that:

$$\Delta Qim + \Delta Qi = 0 \quad (26b)$$

ΔQim can be expressed in terms of Mi changes during Δt as follows:

$$\Delta Qim = Mi^t(1 - CVF^t) - Mi^{(t-\Delta t)}(1 - CVF^{(t-\Delta t)}) \quad (26c)$$

Replacing ΔQim by $-\Delta Qi$ (eq 26b) in equation 26c and solving for Mi^t, we obtain:

$$Mi^t = \frac{Mi^{(t-\Delta t)}(1 - CVF^{(t-\Delta t)}) - \Delta Qi}{1 - CVF^t} \quad (26d)$$

With eq 26d we can now compute the new medium concentrations at time = t for transported solutes, eqs 27a-f, and

for impermeant solutes ($\Delta Qi = 0$) whose concentration changes only because of water shifts, eqs 27g-k:

$$MNa^t = \frac{MNa^{(t-\Delta t)}(1 - CVF^{(t-\Delta t)}) - \Delta QNa}{1 - CVF^t} \tag{27a}$$

$$MK^t = \frac{MK^{(t-\Delta t)}(1 - CVF^{(t-\Delta t)}) - \Delta QK}{1 - CVF^t} \tag{27b}$$

$$MA^t = \frac{MA^{(t-\Delta t)}(1 - CVF^{(t-\Delta t)}) - \Delta QA}{1 - CVF^t} \tag{27c}$$

$$MCa^t = \frac{MCa^{(t-\Delta t)}(1 - CVF^{(t-\Delta t)}) - \Delta QCa}{1 - CVF^t} \tag{27d}$$

$$MMg^t = \frac{MMg^{(t-\Delta t)}(1 - CVF^{(t-\Delta t)}) - \Delta QMg}{1 - CVF^t} \tag{27e}$$

$$MBH^t = \frac{MBH^{(t-\Delta t)}(1 - CVF^{(t-\Delta t)}) - \Delta QH}{1 - CVF^t} \tag{27f}$$

$$MB^t = \frac{MB^{(t-\Delta t)}(1 - CVF^{(t-\Delta t)})}{1 - CVF^t} \tag{27g}$$

$$M^t_{gluconate} = \frac{M^{(t-\Delta t)}_{gluconate}(1 - CVF^{(t-\Delta t)})}{1 - CVF^t} \tag{27h}$$

$$M^t_{glucamine} = \frac{M^{(t-\Delta t)}_{glucamine}(1 - CVF^{(t-\Delta t)})}{1 - CVF^t} \tag{27j}$$

$$M^t_{sucrose} = \frac{M^{(t-\Delta t)}_{sucrose}(1 - CVF^{(t-\Delta t)})}{1 - CVF^t} \tag{27k}$$

With MBH^t and MB^t from eqs 27e-f we can now compute the new medium proton concentration MH^t by solving eq 2 for MH, so that:

$$MH^t = KB\frac{MBH^t}{MB^t - MBH^t} \tag{28a}$$

With MH^t, we can now compute pHm^t, and also the proton and anion concentration ratios across the membrane, rH^t and rA^t, respectively, critical parameters for driving the proton transport dynamics in the model ([34]; User Guide).

$$pHm^t = -\log MH^t \tag{28b}$$

$$rH^t = \frac{MH^t}{CH^t} \tag{28c}$$

$$rA^t = \frac{CA^t}{MA^t} \tag{28d}$$

This completes the list of sequential computations within each iteration cycle of the core red cell model.

There is a substantial body of additional information implemented in the model, marginal to the core of the governing equations, information revised, filtered, and, when experimentally tested and confirmed, incorporated in the model as part of the continuous process of improving the quantitative accuracy of model predictions. The effects of temperature on transport, in addition to those incorporated within the kinetic description (constant field equation, ziFEm/RT, for instance) are represented by factors using the traditional Q10 phenomenology, with the option to alter the default values of 4 and 2 for active and passive transport, respectively. Most ion effects render themselves to be represented by factors modifying rate constants or dissociation constants in flux equations (e.g Na/K pump, eq 11), without altering the actual overall kinetics of the transport pathways. Because of their particular relevance to the circulatory behaviour of RBCs we only listed here explicitly the modulating effects of Ca^{2+} on $P_{GGardos}$ and on P_GCa, equations 14 and 15, respectively. The complete model code is available with open access in the repository (https://github.com/sdrogers/redcellmodeljava).

References

[1] S Rogers and VL Lew. "PIEZO1 and the mechanism of the long circulatory longevity of human red blood cells". *PLoS Comput Biol.* 17.3 (2021), e1008496. DOI: 10.1371/journal.pcbi.1008496. PubMed PMID: 33690597.

[2] S Rogers and VL Lew. "Up-down biphasic volume response of human red blood cells to PIEZO1 activation during capillary transits". *PLoS Comput Biol.* 17.3 (2021), e1008706. DOI: 10.1371/journal.pcbi.1008706. PubMed PMID: 33657092.

[3] R Whittam. *Transport and diffusion in red blood cells.* London: Edward Arnold, 1964.

[4] A Cass and Dalmark M. "Equilibrium dialysis of ions in nystatin-treated cells". *Nature New Biol.* 244 (1973), pp. 47–9.

[5] HG Ferreira and VL Lew. "Use of ionophore A23187 to measure cytoplasmic Ca buffering and activation of the Ca pump by internal Ca". *Nature* 259 (1976), pp. 47–9.

[6] T Tiffert and VL Lew. "Cytoplasmic calcium buffers in intact human red cells". *J Physiol.* 500 (1997). PubMed PMID: 9097939; PubMed Central PMCID: PMCPMC1159365.

[7] P Flatman and VL Lew. "Use of ionophore A23187 to measure and to control free and bound cytoplasmic Mg in intact red cells". *Nature* 267 (1977), pp. 360–2. DOI: 10.1038/267360a0. PubMed PMID: 325421.

[8] JE Raftos, VL Lew, and PW Flatman. "Refinement and evaluation of a model of Mg2+ buffering in human red cells". *Eur J Biochem.* 263.3 (1999). DOI: 635-45.

[9] Z Etzion et al. "Effects of deoxygenation on active and passive Ca^{2+} transport and on the cytoplasmic Ca^{2+} levels of sickle cell anemia red cells". *Journal of Clinical Investigation* 92 (1993), pp. 2489–98.

[10] T Tiffert et al. "Effects of deoxygenation on active and passive Ca^{2+} transport and cytoplasmic Ca^{2+} buffering in normal human red cells". *J Physiol.* 464 (1993), pp. 529–44.

[11] OE Ortiz, VL Lew, and RM Bookchin. "Deoxygenation permeabilizes sickle cell anaemia red cells to magnesium and reverses its gradient in the dense cells". *J Physiol.* 427 (1990), pp. 211–26.

[12] CJ Freeman et al. "K-permeabilized human red cells lose an alkaline, hypertonic fluid containing excess K over diffusible anions". *J Membrane Biol.* 96 (1987), pp. 235–41.

[13] P Swietach et al. "Hydrogen ion dynamics in human red blood cells". *J Physiol.* 588.24 (2010), pp. 4995–5014.

[14] M Waldecker et al. "Differential time-dependent volumetric and surface area changes and delayed induction of new permeation pathways in P. falciparum-infected hemoglobinopathic erythrocytes." *Cell Microbiol.* 19.2 (2017). DOI: 10.1111/cmi.12650.

[15] RP Garay and PJ Garrahan. "The interaction of sodium and potassium with the sodium pump in red cells." *J Physiol (Lond).* 231.2 (1973), pp. 297–325.

[16] PJ Garrahan and RP. Garay. "A kinetic study of the Na pump in red cells: its relevance to the mechanism of active transport." *Ann N Y Acad Sci.* 242 (1974), pp. 445–58.

[17] V Niggli, E Sigel, and E. Carafoli. "The purified Ca^{2+} pump of human erythrocyte membranes catalyzes an electroneutral Ca^{2+}-H^{+} exchange in reconstituted liposomal systems." *J Biol Chem.* 257.5 (1982), pp. 2350–6.

[18] RC Thomas. "The plasma membrane calcium ATPase (PMCA) of neurones is electroneutral and exchanges 2 H^{+} for each Ca^{2+} or Ba2+ ion extruded". *J Physiol.* 587.2 (2009), pp. 315–27. DOI: 10.1113/jphysiol.2008.162453. PubMed PMID: 19064619; PubMed Central PMCID: PMCPMC2670047.

[19] DE Goldman. "Impedance, and Rectification in Membranes". *J Gen Physiol.* 27.1 (1943), pp. 37–60. DOI: doi:10.1085/jgp.27.1.37. PubMed PMID: 19873371; PubMed Central PMCID: PMCPMC2142582.

[20] TJB Simons. "Calcium-dependent potassium exchange in human red cell ghosts". *J Physiol.* 256 (1976), pp. 227–44.

[21] VL Lew and HG Ferreira. *Calcium transport and the properties of a calcium-activated potassium channel in red cell membranes.* Vol. 10. Academic Press, NY, 1978, pp. 217–77.

[22] T Tiffert, J Garcia-Sancho, and VL Lew. "Irreversible ATP depletion caused by low concentrations of formaldehyde and of calcium-chelator esters in intact human red cells". *Biochim Biophys Acta.* 773.1 (1984), pp. 143–56. PubMed PMID: 6428450.

[23] BC Pressman. "Biological applications of ionophores". *Annual Reviews of Biochemistry* 45 (1976), pp. 501–30.

[24] VL Lew et al. "Compartmentalization of sickle-cell calcium in endocytic inside-out vesicles". *Nature* 315.6020 (1985), pp. 586–9. PubMed PMID: 4010773.

[25] VL Lew, S Muallem, and CA Seymour. "Properties of the Ca^{2+}-activated K+ channel in one-step inside-out vesicles from human red cell membranes". *Nature* 296 (1982), pp. 742–4.

[26] LO Simonsen and VL Lew. "The correlation between ionophore A23187 content and calcium permeability of ATP-depleted human red blood cells". *Membrane Transport in Erythrocytes* (1980), pp. 208–12.

[27] JE Raftos and VL Lew. "Effect of intracellular magnesium on calcium extrusion by the plasma membrane calcium pump of intact human red cells". *J Physiol.* 489 (1995), pp. 63–72.

[28] VL Lew and LO Simonsen. "Ionophore A23187-induced calcium permeability of intact human red blood cells". *J Physiol.* 308 (1980), p. 60.

[29] LO Simonsen, J Gomme, and VL Lew. "Uniform ionophore A23187 distribution and cytoplasmic calcium buffering in intact human red cells". *Biochim Biophys Acta.* 692 (1982), pp. 431–40.

[30] J García-Sancho and VL Lew. "Heterogeneous calcium and adenosine triphosphate distribution in calcium-permeabilized human red cells". *J Physiol.* 407 (1988), pp. 523–39.

[31] J García-Sancho and VL Lew. "Detection and separation of human red cells with different calcium contents following uniform calcium permeabilization". *J Physiol.* 407 (1988), pp. 505–22.

[32] T Tiffert, JL Spivak, and VL Lew. "Magnitude of calcium influx required to induce dehydration of normal human red cells". *Biochim Biophys Acta.* 943 (1988), pp. 157–65.

[33] PW Flatman and VL Lew. "The magnesium-dependence of sodium:potassium and sodium:sodium exchange mediated by the sodium pump in intact human red cells [proceedings]." *J Physiol.* 287 (1979), pp. 33–34. PubMed PMID: 430415.

[34] VL Lew and RM. Bookchin. "Volume, pH, and ion-content regulation in human red cells: analysis of transient behavior with an integrated model". *J Membr Biol.* 92.1 (1986), pp. 57–74. PubMed PMID: 3746891.
