# Supplementary material for: The circulatory dynamics of human red blood cell homeostasis: Oxy-deoxy and PIEZO1-triggered changes
Source: Biophys J. 2022 Dec 31;122(3):484–95. doi: 10.1016/j.bpj.2022.12.038 (PMC9941722; doi:10.1016/j.bpj.2022.12.038)
Supplement: Document S2. Article plus supporting material [file mmc2.pdf]

Biophysical Journal
Article

The circulatory dynamics of human red blood cell homeostasis: Oxy-deoxy and PIEZO1-triggered changes

Virgilio L. Lew[1,*]
[1]Physiological Laboratory, Department of Physiology, Development and Neuroscience, University of Cambridge, Downing Site, Cambridge, United Kingdom

ABSTRACT The vital function of red blood cells (RBCs) is to mediate the transport of oxygen from lungs to tissues and of CO_2 from tissues to lungs. The gas exchanges occur during capillary transits within fractions of a second. Each oxygenation-deoxygenation and deoxygenation-reoxygenation transition on hemoglobin triggers sharp changes in RBC pH, leading to downstream changes in ion fluxes, membrane potential, and cell volume. The dynamics of these changes during the variable periods between capillary transits in vivo remains a mystery inaccessible to study by current methodologies, a knowledge gap on a fundamental physiological process that is the focus of the present study. The use of a computational model of human RBC homeostasis of tested accreditation enabled a detailed investigation of the expected RBC changes during intercapillary transits, with results advancing novel insights and predictions. The predicted rates of relative RBC volume change on oxygenation-deoxygenation (oxy-deoxy) and deoxygenation-reoxygenation transitions were about 1.5%/min and −0.9%/min, respectively, far too slow to allow the cells to reach steady states in the intervals between capillary transits. The amplitude of the oxy-deoxy-reoxygenation volume fluctuations varied in proportion with the duration of the intercapillary transit intervals. Upon capillary entry, oxy-deoxy-induced changes occur concurrently with deformation-induced PIEZO1 channel activation, both processes affecting cell pH, membrane potential, and cell volume during intertransit periods. The model showed that the effects were strictly additive as expected from processes operating independently on the cell's homeostatic fabric. Analysis of the mechanisms behind these predictions revealed, for the first time, the complex interactions between oxy-deoxy and ion transport processes that ensure the long-term homeostatic stability of RBCs for optimal gas transport in physiological conditions and how these may become altered in diseased states. Possible designs of microfluidic devices to test the model predictions are discussed.

SIGNIFICANCE Few biological investigations have been pursued with comparable intensity and continuity over the last 130 years as the gas transport function of red blood cells. Against this background, it is surprising to find a major knowledge gap concerning the dynamics of the changes human red blood cells experience during the cyclical oxygenation-deoxygenation of hemoglobin in the circulation in vivo. A well-tested model of red cell homeostasis enabled a detailed in silico investigation of the expected changes predicting relentless cyclical variations in cell pH, membrane potential, and cell volume throughout the cell's lifespan. Analysis of the mechanisms behind these predictions revealed the entangled web of transporter interactions at work. Suggestions for new experimental tests of the model predictions are discussed.

INTRODUCTION

Red blood cells (RBCs) are the most abundant cells in the body. Their main function is to ferry oxygen from lungs to tissues and CO_2 from tissues to lungs, a task each cell performs from about 1000 to 2000 times daily. The full extent of the gas exchange is accomplished during the fraction of second it takes each RBC to traverse a capillary. In the following 30 to 120 s between successive capillary transits, the hemoglobin within each cell remains in a nearly fully oxygenated or deoxygenated condition in the systemic arterial or venous circulation, respectively.

The binding and release of oxygen on capillary transits alters the proton binding properties of hemoglobin, causing sharp intracellular pH changes (1–4). The altered proton

Submitted August 16, 2022, and accepted for publication December 30, 2022.

*Correspondence: vll1@cam.ac.uk

Editor: Valeria Vasquez.
https://doi.org/10.1016/j.bpj.2022.12.038

© 2022 Biophysical Society.
This is an open access article under the CC BY license (http://creativecommons.org/licenses/by/4.0/).

gradient across the RBC membrane triggers ion fluxes leading to secondary changes in RBC pH, membrane potential, volume, cytoplasmic free magnesium concentration, and other cell variables. The condition of RBCs kept in oxygenation (oxy) and deoxygenation (deoxy) steady states in vitro has been extensively investigated and the mechanisms accounting for the oxy-doxy differences fully elucidated (4–18).

The open questions concern the dynamics of the oxy-deoxy-elicited RBC changes under physiological conditions in the circulation in vivo, questions that remain inaccessible to experimental scrutiny. Specifically, can RBCs reach the oxy or deoxy steady states known from experiments in vitro within the range of time intervals between successive capillary transits in vivo? This is the main focus of the investigation reported here.

Concurrent with the oxy-deoxy effects, the squeeze RBCs experience upon each capillary entry opens mechanosensitive PIEZO1 channels in the RBC membrane for a fraction of a second, allowing a brief and transient net calcium influx to trigger a complex cascade of downstream ion fluxes and minute volume changes (5,19–25). How the combined effects of oxy-deoxy transitions and PIEZO1 activation during the first second of capillary ingress influence the time course of RBC changes in the time intervals between capillary transits in vivo is the additional quest in the present study.

To address these questions, use of a tried and tested model of RBC homeostasis (5,19,26–29) allowed an in-depth investigation of the expected kinetics of RBC homeostatic changes in the circulation in vivo. The results predicted a highly dynamic condition with continuously changing RBC volumes, pH, and membrane potentials throughout the lifespan of the cells, with values oscillating within margins well short of those characterized in vitro for RBCs in oxy or deoxy pump-leak steady states.

MATERIALS AND METHODS

The RBC model (RCM)

The model version used for the current investigation (5,19) is available for download with open access from a GitHub repository (https://github.com/sdrogers/redcellmodeljava) together with a comprehensive user guide and tutorial. An updated file with the governing equations of the model is offered here as supporting material. The model operates as a program within the JAVA environment. The name of the program, "RCM*.jar", contains coded information on date and update status and is best retained unchanged. The version used for the simulations reported here was "RCM_8560ca5.jar".

Model simulations follow user-generated instructions recorded in editable protocol files (*.txt). Protocols start by defining the constitutive properties of the RBC under study in an initial oxy steady-state condition, the reference state (RS). The RS entry is followed by sequences of dynamic state instructions designed to emulate stages in experiments or physiological processes. The results are reported in *.csv file format. The columns in the *.csv files display all the variables of the system. The rows report their changing values with time. Running time is always displayed in column one (in min). The RS condition of the RBC suspension used for the simulations reported here corresponds with that of an oxy-RBC with a mean cell hemoglobin concentration of 34 g/dL in a pump-leak steady state suspended in a buffered plasma-like medium.

Simulating the oxy-deoxy-reoxygenation (reoxy) circulatory dynamics of RBC homeostasis

The physiological link between oxy-deoxy transitions and RBC homeostasis is the isoelectric point of hemoglobin (Hb), pI. The model equations translate the instant pI changes induced by oxy-deoxy transitions (1,2) into cell pH changes, as required by charge conservation (30,31). The red cell model was therefore configured to represent such transitions by using the measured pI(0°C) change from 7.2 to 7.5 (1,2). Preservation of electroneutrality required that the net charge on Hb, nHb, remained invariant as pI changed (30). The link between nHb, pI, and cell pH is given by the Dalmark equation:

$$nHb = \alpha(pI - pH),$$

where α is the slope of the proton titration curve of Hb in intact RBCs ($\alpha = -10$ Eq/(mol*Δ(pH – pI)) for HbA).

The following equation preserves nHb during oxy-deoxy transitions:

$$nHb = \alpha\left(pI_{oxy} - pH_{oxy}\right) = \alpha\left(pI_{deoxy} - pH_{deoxy}\right).$$

From this equation, we derive the new cell pH, pH_{deoxy}, after an oxy-deoxy transition, as follows:

$$pH_{deoxy} = pH_{oxy} + \left(pI_{deoxy} - pI_{oxy}\right), \quad (1)$$

and the initial pH on reoxy, pH_{oxy}, will be

$$pH_{oxy} = pH_{deoxy} + \left(pI_{oxy} - pI_{deoxy}\right). \quad (2)$$

Following the initial ΔpHi after each transition, the model computes the evolution in time of all the homeostatic variables of the system. The parameter values used for the current simulations were all experimentally determined within coefficients of variation of between 5% and 15% (1,2,13–15,30–35).

Simulating the PIEZO1 triggered changes in RBC homeostasis

Concurrently with the oxy-deoxy transitions on capillary entry, the sub-second open state of the PIEZO1 channels allows a sharp inflow of $CaCl_2$ and water driven by the huge inward electrochemical gradient for Ca^{2+}. Calcium influx, in turn, elevates $[Ca^{2+}]_i$ to levels that activate Ca^{2+}-sensitive K^+ channels (Gardos channels, KCNN4, (36–39)), causing secondary loss of KCl and water. The Ca^{2+} effects are cut short by Ca^{2+} extrusion through the powerful plasma membrane calcium pump (plasma membrane calcium pump 4b, (40–43)) rapidly restoring baseline $[Ca^{2+}]_i$ levels. The Cl^- gained during the initial $CaCl_2$ inflow is restored by proton-driven Cl^- efflux via the Jacob-Stewart mechanism with parallel osmotic-driven cell volume reduction. The net loss of KCl and water during the brief open period of the Gardos channels generates a tendency for cell volume to decrease between consecutive capillary transits, a tendency that declines with cell age because of the exponential fall in the calcium extrusion capacity of the pump (43,44). These sequential events generate an up-down biphasic volume response, with RBC volume increasing by up to 0.005% at the peak and falling below baseline levels by less than 0.0001% within a minute or two. These are infinitesimal volume displacements but have the potential to increase RBC density over myriad capillary transits as the cells age in the circulation (5,19). This response and its complex mechanism have been analyzed and reported in detail before but only for oxy conditions (5). The new question

addressed here is whether PIEZO1 and oxy-deoxy processes interact and influence each other during intercapillary transits.

Representation of the extracellular medium in the simulations

In vivo, plasma pH is essentially the same for arterial or venous blood because CO_2 volatility and the respiratory apparatus effectively provide unlimited buffering capacity to the plasma, with minor contributions of histidine residues from plasma albumin (10). Plasma pH invariance can only be represented in a mass-conservation model structured as a closed cell-medium two-compartment system by running the simulations at a vanishing low cell volume fraction, approximating the condition of an open system with a constant medium composition.

The protocols used for the simulations shown in Figs. 1–5 are shown sequentially within a pdf file as supporting material SM1. SM2 presents a new version of the governing equations of the red blood cell model, updated to include all equations associated with oxy-deoxy transitions and cytoplasmic magnesium buffering used within RCM_8560ca5.jar in the present study. The SM1 and SM2 files are combined into a single PDF file.

RESULTS

The oxy-deoxy-induced changes in RBC homeostatic variables are considered first, followed by those elicited by PIEZO1 activation, in isolation and in combination with oxy-deoxy changes. The emphasis throughout the presentation of the model results is on the mechanisms behind the predicted effects.

The kinetics of oxy-deoxy-elicited changes in RBC homeostasis

The panels of Fig. 1 illustrate, from top to bottom, the sequential changes in selected RBC variables triggered by oxy-deoxy-reoxy transitions, as predicted by the model. The sudden initial pI change elevates cell pH (Fig. 1 *A*; Eq. 1), driving all subsequent downstream changes in homeostatic variables (Fig. 1, *B* to *D*). Deoxy elevates cell pH by about 0.3 units (Fig. 1 *A*), reflecting a sharp initial reduction in cytoplasmic $[H^+]_i$ from ~60 to ~30 nM.

At constant extracellular pH, the suddenly increased inward proton gradient (Fig. 1 *B*, *black curve*) activates a large net H:Cl influx through the Jacob-Stewart mechanism (Fig. 1 *C*) attempting to equalize the displaced proton and chloride concentration ratios toward the zero net flux Jacob-Stewart equilibrium condition, $[H^+]_i/[H^+]_o = [Cl^-]_o/[Cl^-]_i$ (Fig. 1 *B*). Net chloride influx, in turn, elevates cell osmolarity, thus increasing cell volume by up to about 4% in the deoxygenated steady state (Fig. 1 *D*). The deoxy-induced volume increase is therefore the direct result of the Jacob-Stewart mechanism attempting to restore the $[Cl^-]_o/[Cl^-]_i = [H^+]_i/[H^+]_o$ equality. Reoxy reverses the direction of all these processes, starting with a reoxy-induced decrease in pH (Eq. 2; Fig. 1 *A*).

This sequence sums up the known mechanisms responsible for the effects of oxy-deoxy transitions on RBC pH and volume. The predicted steady-state pH and volume levels follow closely the experimentally measured ones (30,31). The experimental results were obtained using RBC suspensions at higher cell volume fractions than those simulated here. Whereas at low cell fractions, pH and volume changes are maximally absorbed by the cells, at higher cell fractions, the spread of changes between cells and medium reduces their amplitude in the cells, thus explaining why the experimental and in vivo volume increases in deoxy states remain close to, but below, the ~4% predicted maxima.

The predictions in Fig. 1 show, for the first time, the dynamic responses of the main variables controlling RBC volume in response to oxy-deoxy-reoxy transitions as expected to take place in the circulation in vivo.

The rates at which RBC volumes approach steady states after oxy-deoxy-reoxy transitions were estimated from exponential fits to the red cell volume (RCV) curves in Fig. 1 *D*. The oxy-deoxy RCV transition rate was about 1.5%/min, and the deoxy-reoxy rate was about −0.9%/min. This asymmetry in magnitude can be traced to differences in the initial peak displacements of cell pH (Fig. 1 *A*), of $[H^+]_i/[H^+]_o$ (Fig. 1 *B*), and of Cl^- flux (Fig. 1 *C*), the sequential drivers of the processes leading to the volume change (Fig. 1 *D*). The important new insight here is that the rates of volume change are too slow to allow steady states to be approached between capillary passages unless blood flow becomes locally arrested for longer periods (45,46).

The simulations in Fig. 2 illustrate the extent to which the amplitude of the RBC volume changes may depart from oxy-deoxy steady-state values in circulatory conditions. The figure shows, from left to right, three sequential trains of oxy-deoxy cycles set for intertransit intervals of 1.5, 1, and 0.5 min. It can be seen how steeply the amplitude of the volume excursions becomes reduced the shorter the intertransit intervals. The trains are shown separated by 10 min segments in oxy or deoxy conditions to allow for a direct visual comparison of amplitudes relative to oxy or deoxy steady-state levels.

The fourth train shown on the right of Fig. 2, designed with a protocol of stochastic intertransit durations between 0.5 and 1.5 min, represents the more realistic in vivo condition for individual RBCs traversing variable distances between sequential capillary circuits in the systemic circulation. In days-long simulations (data not shown), the interspersed 10 min comparative steady-state volume levels remained invariant, suggesting that oxy-deoxy cycling generates no cumulative changes on the basic homeostatic configuration of the RBCs in the circulation a critical distinction from the PIEZO1-elicited effects (19).

Is the deoxy steady state a real steady state?

An unexpected prediction of the model was that the rapid volume increase induced by deoxy was not toward a real steady state. On the time scale of Figs. 1 *D* and 2, it was

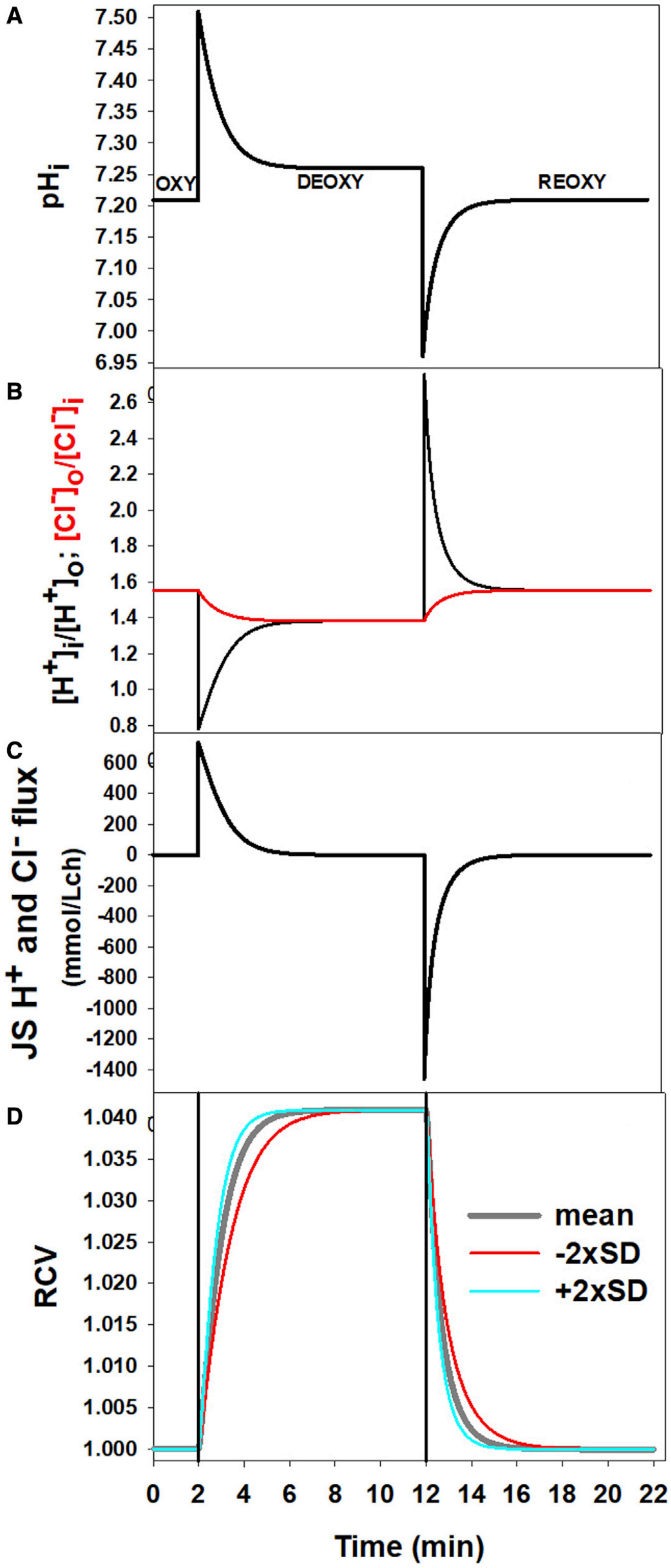

FIGURE 1 Time course of the changes in RBC homeostatic variables triggered by oxy-deoxy transitions on the isoelectric point of hemoglobin (pI). The time scale for this simulation was chosen to allow the variables to reach effective steady states. (*A*) The first effect of the instant (subsecond) oxy-to-deoxy pI transition is a sharp increase in cell pH followed by a slower recovery toward a deoxy steady state above oxy baseline levels. Upon reoxy, the peak excursion from the deoxy level is larger, but the slow return to the oxy steady state is shorter, establishing a kinetic asymmetry between oxy and deoxy transitions. (*B*) Deoxy lowers the H^+ and Cl^- concentration ratios across the RBC membrane relative to their initial oxy equality. The equalities are gradually restored to the oxy level upon reoxy. (*C*) H^+ and Cl^- fluxes through the Jacob-Stewart (JS) mechanism operating as an H^+:Cl^- cotransporter (5,26,45). The areas between oxy-deoxy and deoxy-reoxy steady states are the same as expected for balanced fluxes returning to initial conditions. (*D*) The maximal amplitude predicted for the oxy-deoxy volume displacements amounts to about 4%. The mean curve (*black*) was computed using the measured mean rate constant for JS-mediated H^+ and Cl^- fluxes. The thin lines (*red and cyan*) were computed using rate constant values set at twice the measured SD of the rate constant distribution (34). Note that the predicted time course to steady-state volume is around 4–5 min, far longer than most intercapillary transit times in the 0.5 to 2.0 min range. To see this figure in color, go online.

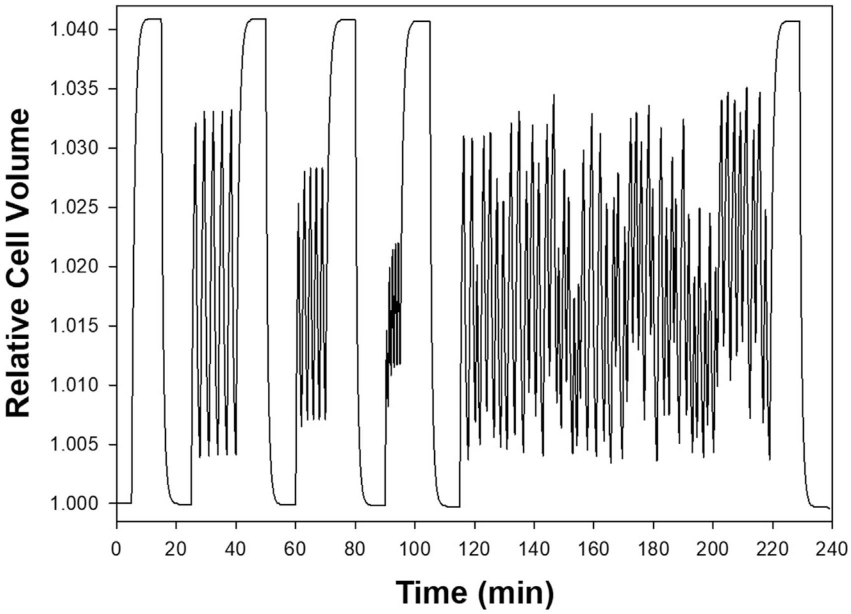

FIGURE 2 Relation between predicted amplitudes of RBC volume changes during oxy-deoxy-reoxy cycling and duration of the intercapillary transit intervals. In the protocol for this simulation, oxy-deoxy cycling trains were separated by 10 min cycling breaks to allow easy visual comparisons of cycling amplitudes with oxy-deoxy steady-state volume levels. From left to right, the duration of the first three intertransit intervals was set to 1.5, 1.0, and 0.5 min. In the fourth sequence, intertransit interval times were set to vary at random between 0.5 and 1.5 min. The random cycling train was explored for up to 10 days in additional simulations, with no detectable shifts in the interspersed steady-state volume levels recorded at regular intervals.

very hard to discern that the deoxy plateau described as a steady state was not quite steady. Over much longer time scales (Fig. 3), the relative cell volume of RBCs kept in deoxy conditions declined slowly but steadily toward a real pump-leak steady state below that of the oxy RBCs, a relative volume decrease of about 7%. It can be seen that immediately after an oxy-deoxy transition, the apparent steady-state volume response shown in Fig. 1 *D* remains hidden within the peak volume increase when displayed on the vastly expanded time scale of Fig. 3.

Exploring this response further over extended periods of time (Fig. 3, *inset*), the results showed fully reversible long-term transitions between real steady states through transient peak-induced breaks at the oxy-deoxy and deoxy-reoxy turning points. Although the short-term deoxy quasi steady states shown in Figs. 1 *D* and 2 do not represent real steady states, they do represent the only condition of physiological relevance for determining the direction of volume changes during oxy-deoxy transitions in vivo within realistic intercapillary transit times (Fig. 2).

The kinetics of PIEZO1-elicited changes in RBC homeostasis following capillary ingress

The panels in Fig. 4 sum up, from top to bottom, the sequence of PIEZO1-triggered changes following a brief subsecond open state (5,24,47). The use of a common time scale for the three sequential processes (Fig. 4, *A–C*, *cyan curves*) exposes more clearly how sharp and brief transport changes can elicit delayed volume responses (Fig. 4 *D*). The slow volume response results from the extremely low constitutive cation permeability of the RBC membrane rate limiting the restorative net salt and fluid movements. The insets in Fig. 4, *A–C*, show the initial displacements on a largely expanded *x* axis time scale for detail.

The mechanism shaping the volume response to PIEZO1 activation starts with a massive calcium influx driven by its huge inward electrochemical gradient (Fig. 4 *A*) during the subsecond duration of the PIEZO1 channel openings. Elevated $[Ca^{2+}]_i$ stimulates the plasma membrane calcium pump to rapidly extrude the calcium gained as shown by the area within the negative flux dip in Fig. 4 *A*, with extrusion accomplished in about 0.1 min (Fig. 4 *A*, *inset*). During the period of elevated $[Ca^{2+}]_I$, Gardos channel activity mediates a large net K^+ efflux (Fig. 4 *B*), leading to a net loss of KCl and water from the cell. The Cl^- gained during the initial $CaCl_2$ influx is rapidly extruded by the Jacob-Steward mechanism (Fig. 4 *C*) operating as an electroneutral Cl^-:H^+ cotransport (26,48). The net loss of KCl and water during the brief period of Gardos channel opening leads to a slow cell volume readjustment toward a slightly dehydrated level after each capillary transit, amounting to a relative volume decrease of less than 0.00001% (Fig. 4 *D*). This slow volume change is led by a minute residual Cl^- efflux shown on a vastly expanded *y* axis scale in the inset of Fig. 4 *D* but with the capacity to cause cumulative volume changes in association with age-dependent declines in plasma membrane calcium pump activity (5,19).

Exploring interactions between oxy-deoxy- and PIEZO1-mediated processes

Within the first second of capillary entry, gas exchange is completed (49–51), and PIEZO1 channels open and close (19). Fig. 5 shows how these two transients affect selected homeostatic variables in the intervals between capillary

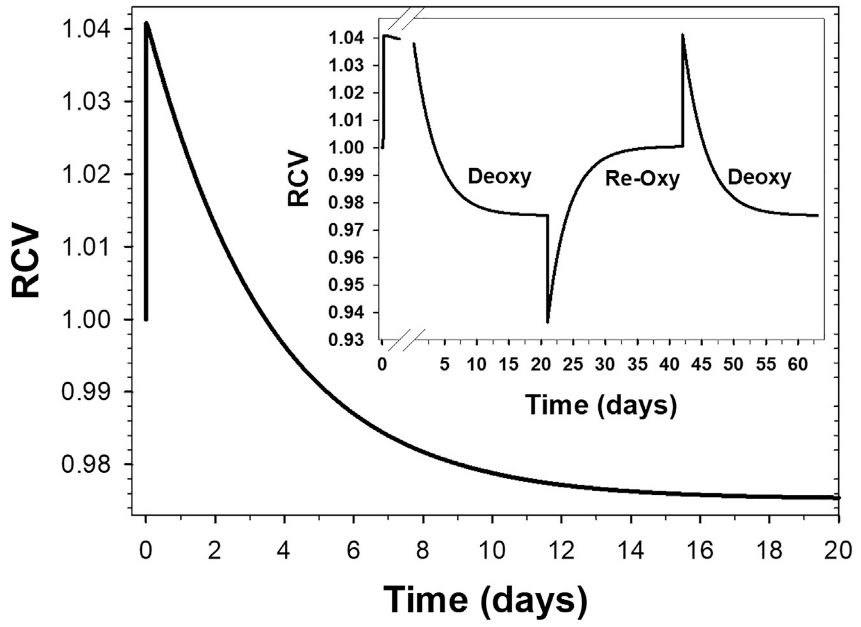

FIGURE 3 Exploring the long-term evolution of the volume changes induced by oxy-deoxy transitions in human RBCs kept under deoxygenated conditions. After the initial volume increase reported in Fig. 1 *D*, the model predicted a steady volume decline at rates increasing from 1 to 2 $\times$ 10^{-5} per min. The final steady volume level at day 20 was 0.975, a 6.6% fall from the initial physiological quasi steady state of 1.041. On an expanded initial time scale (*inset*), the declining pattern is clearly noticeable within the first 2–3 days of the deoxy transition. The sequential deoxy-reoxy-deoxy transitions in the inset document the full reversibility of the homeostatic conditions of RBCs transitioning between initial quasi and final true steady states. To see this figure in color, go online.

transits. The simulations were designed to optimize comparisons between separated and overlapping responses on the same time scale. The results in Fig. 5 sum up the main results and conclusions on mutual influences derived from a much wider investigation.

Fig. 5, *A* and *E*, show changes elicited by oxy-deoxy cycling on variables apparently unaffected by PIEZO1 activity. Whereas PIEZO1 has no real effect on the $[Mg^{2+}]_i$ transitions (Fig. 5 *E*), the minute size of the PIEZO RCV signal (Fig. 4 *D*) is present but remains hidden on the scale of the oxy-deoxy RCV displacements (Fig. 5 *A*). Fig. 5, *B–D*, show variables where apparent interactions could be detected.

Fig. 5 *B* shows the composite effects of both processes on the RBC membrane potential. PIEZO1 activation causes a brief depolarization during $CaCl_2$ influx because Cl^- gain reduces the $[Cl^-]_o/[Cl^-]_I$ ratio. As soon as the Ca^{2+} gain activates the Gardos channels increasing the K^+ permeability (Fig. 4 *B*), the membrane potential shifts toward E_K, hyperpolarizing the membrane. As shown in the insets of Fig. 4, *B* and *C*, this biphasic response is nearly completed within 0.1 min, appearing as needle-sharp membrane potential deflections of about 2 mV amplitude on the common time scale of Fig. 5.

Deoxy-reoxy cycles, on the other hand, generate slower depolarization-repolarization transients following the slower proton ratio displacements shown in Fig. 1, *B* and *C*. The PIEZO1 E_m signals remain unaltered when superimposed on the oxy-deoxy E_m signals, indicating the noninteractive additivity of the two processes on this variable.

The calcium flux pattern generated by PIEZO1 activation (Fig. 5 *C*) becomes slightly altered when superimposed on the deoxy-oxy signals. Deoxy reduces both the calcium influx peak through PIEZO1 and the pump-extrusion dip. Reoxy reverses these changes. Model analysis of this interaction offered a straightforward explanation: the depolarization associated with cell swelling on deoxy (Fig. 5 *B*) reduces the driving force for calcium influx through PIEZO1 and consequently also the amount of restorative calcium extrusion required by the pump; repolarization on reoxy reverses these changes but only partially, as RCV remains above oxy baseline levels during deoxy-oxy cycling (Fig. 2).

The deoxy-oxy effects on calcium fluxes shown in Fig. 5 *C* cause secondary effects on the amplitude and time course of the cytoplasmic calcium concentrations controlling the periods of Gardos channel activity, as shown in the inset of Fig. 4 *B*. This explains the synchrony between the oscillations in calcium and potassium fluxes shown in Fig. 5, *C* and *D*.

The results in Fig. 5 *E* confirm that the cytoplasmic magnesium buffering properties encoded in the model represent adequately the well-measured changes in $[Mg^{2+}]_i$ induced by oxy-deoxy transitions (13–15). The binding affinity of Hb for ATP and 2,3-DPG is increased by deoxy (52). ATP and 2,3-DPG are the two main Mg^{2+}-binding compounds in the RBC cytoplasm (53–57). Deoxy reduces their availability for buffering intracellular magnesium with a consequent increase in $[Mg^{2+}]_i$. Reoxy rapidly reverses these changes. The model predicts sharp up and down $[Mg^{2+}]_i$ transitions on deoxy-reoxy transits, with minor volume-related adjustments during the intertransit periods and no detectable influence of PIEZO1-activated processes (Fig. 5 *E*).

Analysis of the results in Fig. 5 leads to the conclusion that oxy-deoxy- and PIEZO1-triggered processes operate additively and independently in the circulation. The minor interactions among homeostatic variables are indirectly generated by membrane-potential-mediated effects.

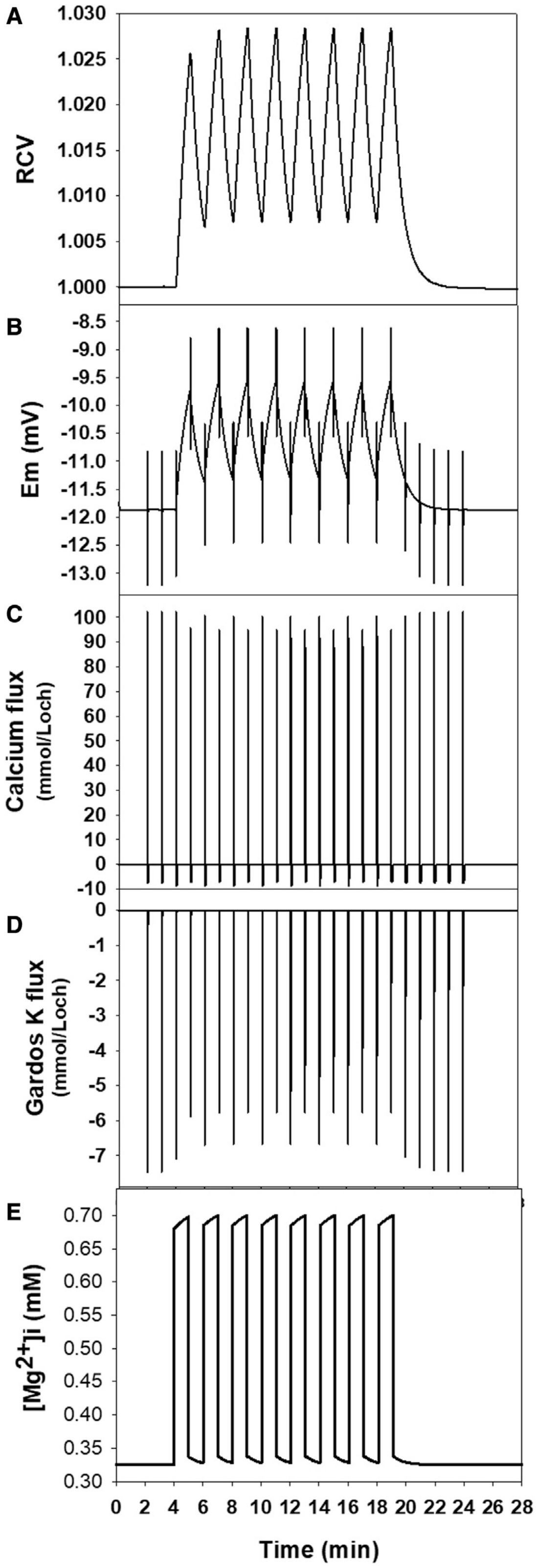

FIGURE 4 The mechanism of the PIEZO1-triggered RBC volume changes during inter-capillary transits. (*A–C*) The sequential steps leading to the up-down biphasic volume response (*D*) elicited by the RBC deformations that activate PIEZO1 channels on capillary ingress. The insets within each panel show detailed changes on expanded *x* or *y* scales. The open state of the PIEZO1 channels was set to 0.4 s in the simulations. The cyan curves in (*A*)–(*C*) follow the *y* axis changes on the same common time scale as (*D*). (*A*) Calcium influx and pump-mediated extrusion. The cyan curve and the inset on an expanded time scale show a brief sharp positive net $Ca2^+$ influx during the open state, instantly reversing to a negative pump-mediated $Ca2^+$ extrusion in two phases: a sustained efflux of about −7 mmol/Loch by a $[Ca2^+]$i-saturated pump followed by a brief $[Ca2^+]$i-desaturation efflux, all essentially over in 6–10 s. (*B*) K^+ fluxes through $Ca2^+$-activated Gardos channels. After the sharp initial K^+ efflux peak, K^+ efflux returns to the zero baseline in less than 6 s following the $[Ca2^+]$i-desaturation kinetics of the channels (49). During this period, the cells experience a net loss of KCl and water. (*C*) Chloride and proton fluxes through the JS complex. The initial Cl^- gain, twice that of $Ca2^+$, is rapidly extruded as an H^+:Cl^- cotransport through the JS complex. However, the equality of the proton and chloride concentration ratios at equilibrium is approached much slower as shown in this inset and in that of (*D*) on a vastly expanded *y* axis scale. (*D*) Predicted volume change following a subsecond PIEZO1 channel activation. Note that the net loss of KCl and water during the Gardos channel activation period (Fig. 4 *B*) leads to cell dehydration during the longer-lasting intertransit periods. Despite the infinitesimal scale of the predicted volume changes relative to those triggered by oxy-deoxy cycling (Fig. 2), only PIEZO1-mediated ones have the potential to cause cumulative RBC hydration changes in the circulation.

DISCUSSION

The main conclusion from the current results is that the rates of volume change following oxy-deoxy transitions are too slow to allow cell volumes to reach steady states in the intervals between capillary transits, forcing continuous volume fluctuations in the circulation throughout the cell's lifespan (Figs. 1 and 2). The amplitude of the volume displacements varied stochastically with the duration of the intercapillary transits (Fig. 2). Multivariable analysis of the combined changes induced by PIEZO1 and by oxy-deoxy transitions revealed that the two processes operate additively and independently, with minimal interactions mediated by membrane potential oscillations (Figs. 4 and 5). The mechanisms behind the predicted oxy-deoxy- and PIEZO1-induced fluctuations in RBC homeostasis in vivo, analyzed in detail in the results, highlight the extraordinary long-term circulatory balance and stability endowed to the cells by the combination of a low constitutive cation permeability of the plasma membrane and the operation of the Jacob-Stewart cycle. As with hemodynamic motion, RBC homeostasis is never at rest in the circulation. RBC pump-leak balanced steady states, whether in oxy or deoxy conditions, are ex vivo experimental constructs.

Although the dynamic changes in homeostatic variables shown in Figs. 1, 2, 3, and 5 cannot be explored in vivo, future technical developments may eventually enable direct experimental tests of the model predictions. One possible approach would be for RBCs from the same batch loaded with either pH, calcium-sensitive, or membrane potential indicators to circulate in single file through microfluidic channels (58) with intermittent constrictions somehow configured to allow alternative exposure of the cells to O_2 or to O_2-free CO_2 during consecutive constriction passages

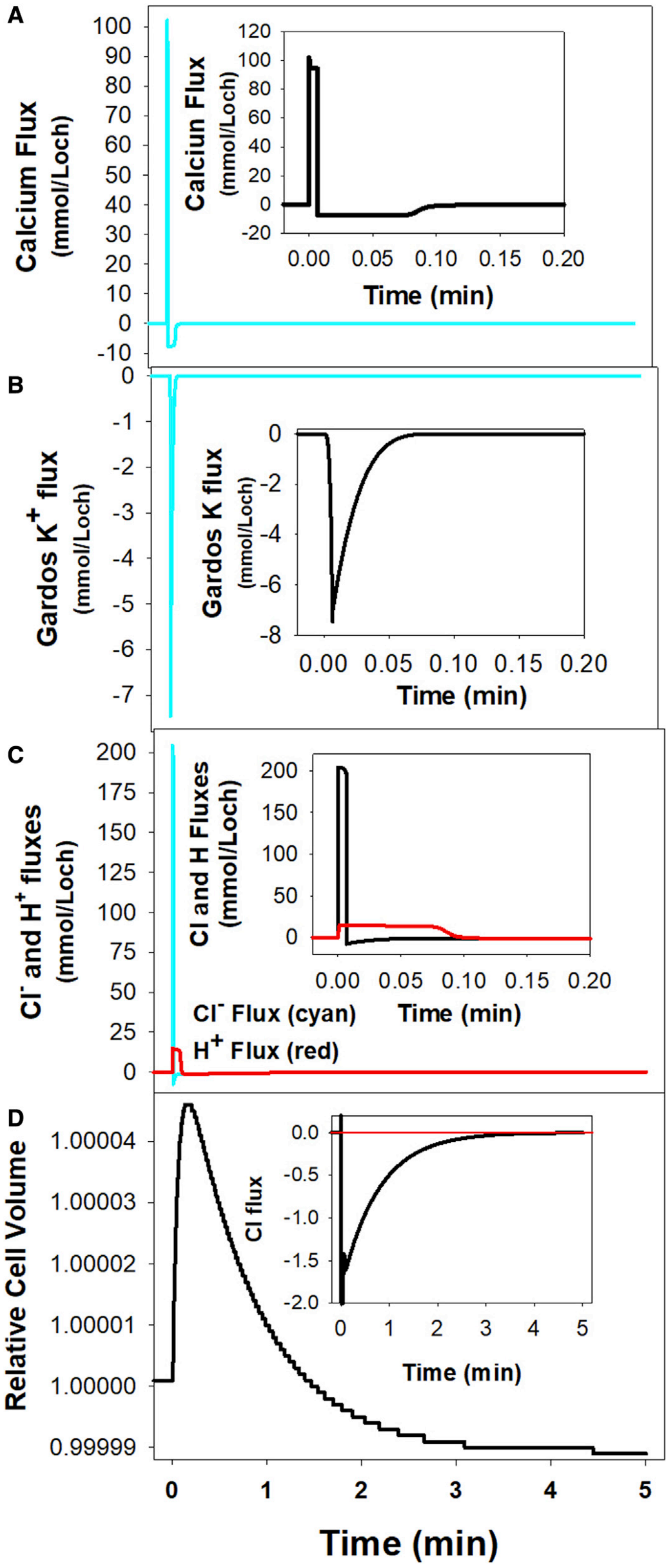

FIGURE 5 Interactions between oxy-deoxy- and PIEZO1-triggered processes during intercapillary transits. In order to explore mutual interactions, the protocol for this figure was designed to show 23 sequential 1 min intercapillary transits with PIEZO1 openings of 0.4 s on capillary ingress and 16 overlapping instant oxy-deoxy transitions in the middle of the PIEZO1 train (see supporting material S1 for protocol details). The selected variables for (*A*)–(*D*) were the only ones that showed detectable interactive influences. (*A*) The minute PIEZO1 volume changes (Fig. 3 *D*) do register but cannot be discerned on the scale of the oxy-deoxy-induced changes shown here. (*B*) The amplitude and brevity of the PIEZO1-induced membrane potential changes remains unaltered when superimposed on those of the much slower oxy-deoxy membrane potential transitions. (*C*) Note that the $Ca2^{+}$ influx peaks appear reduced on deoxy relative to reoxy and so do the plasma membrane calcium pump-mediated dips (Fig. 3 *A*). (*D*) Gardos channel-mediated K^{+} fluxes are similarly reduced on deoxy, suggesting a clear link to the reduced $Ca2^{+}$ influx noted in (*C*). (*E*) The relatively large oxy-deoxy-induced changes in $[Mg2^{+}]$i are fully reversible. There are no detectable $[Mg2^{+}]$i responses to PIEZO1-induced changes in the absence of oxy-deoxy transitions.

while monitoring the dynamic fluctuations in the fluorescent signals. Previous results by Swietach et al., using a pH fluorophore (48), and by Danielczok et al. (59), using a calcium indicator in a microfluidic system to detect PIEZO1-triggered signals while traversing constrictions, show that, in principle, this approach is feasible. However, gas control

TABLE 1 Predicted differences in the values of selected homeostatic variables in oxy and deoxy steady states of RBCs

Parameter or Variable	RCV	nHb mEq/mol	$[Mg^{2+}]$ mM	pH	$[Na^{+}]$ mM	$[K^{+}]$ mM	$[Cl^{-}]$ mM	Em mV
Oxy steady state	1.000	−6.01	0.325	7.22	10.0	140	95.0	−12
Deoxy "physiological" *steady* state	1.041	−3.52	0.708	7.26	9.51	133	107	−8.7
Deoxy final steady state	0.975	−3.33	0.663	7.24	9.40	130	102	−9.9

The values for physiological and final deoxy steady states were taken at 10 min and 20 days, respectively, after the oxy-deoxy transition (Fig. 3).

during constriction passages and maintenance of subtoxic light exposure levels while recording dynamic changes from fluorescent signals over extended periods of time pose technical challenges not yet confronted.

The most unexpected prediction encountered during this investigation was that RBCs kept in deoxygenated conditions after an oxy-deoxy transition would slowly reverse their rapid initial volume gain toward a final volume decline far below that of the initial physiological quasi steady state (Fig. 3; Table 1). To understand the mechanism behind this prediction, it was necessary to explore how the values of the main homeostatic variables that drive RBC volume changes differ in between oxy and deoxy stable states. These values are shown in Table 1, together with nHb, the pI-modified variable from which all other downstream changes between oxy and deoxy conditions arise (Eqs. 1 and 2; Fig. 1).

Table 1 shows that the differences in the concentrations of permeable ions and membrane potentials between the "physiological" deoxy stable state and final deoxy steady state are much smaller than those between oxy and deoxy states. These minute deoxy-state differences point to extremely weak driving gradients for the volume transitions between physiological and final deoxy steady states, thus explaining the exceptional slowness of the predicted transition rates (Fig. 3). The main surprise here was that these same minute differences could ultimately lead to such relatively large RCV differences.

Experimental tests of the predictions in Fig. 3 would require long incubations of deoxygenated RBCs at 37°C and at very low cell volume fractions to ensure the constancy of medium composition, conditions that have been approximated in certain malaria cultures (60–64). It is questionable whether the predicted long-term volume decline of deoxygenated RBCs ever occurs in physiological or pathological conditions in vivo. Therefore, this model prediction ought to be considered of primary academic interest for the time being.

The current study was centered on an RBC modeled with the values of parameters and variables corresponding to the means of measurements performed in blood samples from healthy human adults. In patients with inherited hemolytic anemias associated with RBC hydration disorders, the primary mutations elicit the formation of subpopulations of circulating RBCs with marked differences in volume, intracellular pH, and calcium distributions with patterns specific for each type of anemia (28,52,65,66). In inherited hemoglobinopathies, the abnormal hemoglobins often differ in their proton buffering properties (52,67), the α and pI parameters in the Dalmark equation (1,2). The current modeling study provides the means to investigate the potential effects expected from the abnormal buffering properties of these hemoglobins on the circulatory dynamics of RBCs in these diseases.

Mutant PIEZO1 (22,68,69) and Gardos channels (68,70,71) generate a wide spectrum of hematological abnormalities with varying degrees of RBC hydration disorders, reduced RBC lifespan, anemia, and clinical severity (72). An extension of the RCM used in the current study was recently applied to follow the cumulative effects expected from PIEZO1 activation on capillary entry throughout the nearly 200,000 capillary transits over normal RBC lifespans (19). When applied to investigate the possible effects of deoxy activation of PIEZO1 in sickle cell reticulocytes, the model predicted a rapid hyperdense collapse with a much-reduced RBC lifespan, reminiscent of that documented for irreversibly sickled cells, the cells responsible for microvascular occlusion and organ failures, the major trigger of all downstream effects in sickle cell disease (5,28,73,74).

A common PIEZO1 polymorphism was recently found associated with protection from severe malaria in humans (75), a clear target for a study on the possible mechanism of protection using the model of falciparum-infected RBCs (76). When applied to study the homeostasis of falciparum-infected RBCs in the past (29,61), this model predicted the time course of volume growth of the parasite during its reproduction cycle, a prediction recently verified (60), explained its mechanism, and also showed that digestion of host Hb far in excess of parasites' biosynthetic needs was needed to prevent premature lysis of the infected cell by reducing the colloid osmotic pressure within the host. These examples demonstrate the potential predictive power of cellular homeostasis models to contribute new insights to the understanding of the mechanisms behind complex cell responses.

Nothing is known at present about how the dynamics of oxy-deoxy-, PIEZO1-, and Gardos channel-dependent RBC responses are affected during capillary transits in any of these pathologies and how these responses contribute to the clinical condition. The modeling approach applied in the present investigation opens the way for in-depth studies on the altered circulatory dynamics that may be expected from RBCs modeled with different constitutive properties or infected with malaria parasites, a vast new area of enquiry for future research.

SUPPORTING MATERIAL

Supporting material can be found online at https://doi.org/10.1016/j.bpj.2022.12.038.

ACKNOWLEDGMENTS

The research reported here was supported by funds from The University of Cambridge, UK. V.L.L. is grateful to Simon Rogers for implementing source corrections on the RCM_8560ca5 version of the model used in this investigation and to Teresa Tiffert, Daniel Lew, and Pietro Cicuta for helpful discussions and comments on early drafts.

DECLARATION OF INTERESTS

The author declares no competing interests.

REFERENCES

1. Dalmark, M. 1975. Chloride and water distribution in human red cells. *J. Physiol.* 250:65–84.
2. Dalmark, M. 1975. Chloride transport in human red cells. *J. Physiol.* 250:39–64.
3. Freedman, J. C., and J. F. Hoffman. 1979. Ionic and osmotic equilibria of human red blood cells treated with nystatin. *J. Gen. Physiol.* 74:157–185. http://www.ncbi.nlm.nih.gov/pubmed/490141.
4. Warburg, E. J. 1922. XXII. Studies on carbonic acid compounds and hydrogen ion activities in blood and salt solutions. A contribution to the theory of the equation of Lawrence J. Henderson and K. A. Hasselbalch. *Biochem. J.* 16:153–154.
5. Rogers, S., and V. L. Lew. 2021. Up-down biphasic volume response of human red blood cells to PIEZO1 activation during capillary transits. *PLoS Comput. Biol.* 17:e1008706. https://doi.org/10.1371/journal.pcbi.1008706. https://www.ncbi.nlm.nih.gov/pubmed/33657092.
6. Larsen, E. H., E. Hoffmann, …, T. Wang. 2021. August Krogh's contribution to the rise of physiology during the first half the 20th century. *Comp. Biochem. Physiol. Mol. Integr. Physiol.* 256:110931. https://doi.org/10.1016/j.cbpa.2021.110931. https://www.ncbi.nlm.nih.gov/pubmed/33647460.
7. Hladky, S. B., and T. J. Rink. 1977. pH equilibrium across the red cell membrane. *In* Membrane Transport in Red Cells. J. C. Ellory and V. L. Lew., eds Academic Press, London, pp. 115–135.
8. Bohr, C., K. Hasselbalch, and A. Krogh. 1904. Ueber einen in biologischer Beziehung wichtigen Einfluss, den die Kohlensäurespannung des Blutes auf dessen Sauerstoffbindung übt. *Skandinavisches Archiv Für. Physiologie.* 16:402–412.
9. Krogh, A., and J. Lindhard. 1917. The volume of the dead space in breathing and the mixing of gases in the lungs of man. *J. Physiol.* 51:59–90. https://doi.org/10.1113/jphysiol.1917.sp001785. https://www.ncbi.nlm.nih.gov/pubmed/16993378.
10. Van Slyke, D. D., H. Wu, and F. C. McLean. 1923. Studies of gas and electrolyte equilibria in the blood. V. Factors controlling the electrolyte and water distribution in the blood. *J. Biol. Chem.* 56:765–849.
11. Jacobs, M. H., and D. R. Stewart. 1942. The role of carbonic anhydrase in certain ionic exchanges involving the erythrocyte. *J. Gen. Physiol.* 25:539–552.
12. Jacobs, M. H., and D. R. Stewart. 1947. Osmotic properties of the erythrocyte. XII. Ionic and osmotic equilibria with a complex external solution. *J. Cell. Comp. Physiol.* 30:79–103.
13. Ortiz, O. E., V. L. Lew, and R. M. Bookchin. 1990. Deoxygenation permeabilizes sickle cell anaemia red cells to magnesium and reverses its gradient in the dense cells. *J. Physiol.* 427:211–226.
14. Raftos, J. E., V. L. Lew, and P. W. Flatman. 1999. Refinement and evaluation of a model of Mg2+ buffering in human red cells. *Eur. J. Biochem.* 263:635–645. http://www.ncbi.nlm.nih.gov/pubmed/0010469126.
15. Flatman, P., and V. L. Lew. 1977. Use of ionophore A23187 to measure and to control free and bound cytoplasmic Mg in intact red cells. *Nature.* 267:360–362. https://www.ncbi.nlm.nih.gov/pubmed/325421.
16. Flatman, P. W. 1988. The control of red cell magnesium. *Magnes. Res.* 1:5–11.
17. Benesch, R., and R. E. Benesch. 1967. The effect of organic phosphates from the human erythrocyte on the allosteric properties of hemoglobin. *Biochem. Biophys. Res. Commun.* 26:162–167. https://doi.org/10.1016/0006-291x(67)90228-8. https://www.ncbi.nlm.nih.gov/pubmed/6030262.
18. Benesch, R. E., R. Benesch, and C. I. Yu. 1969. The oxygenation of hemogloin in the presence of 2, 3-diphosphoglycerate. Effect of temperature, pH, ionic strength, and hemoglobin concentration. *Biochemistry.* 8:2567–2571.
19. Rogers, S., and V. L. Lew. 2021. PIEZO1 and the mechanism of the long circulatory longevity of human red blood cells. *PLoS Comput. Biol.* 17:e1008496. https://doi.org/10.1371/journal.pcbi.1008496. https://www.ncbi.nlm.nih.gov/pubmed/33690597.
20. Bae, C., R. Gnanasambandam, …, P. A. Gottlieb. 2013. Xerocytosis is caused by mutations that alter the kinetics of the mechanosensitive channel PIEZO1. *Proc. Natl. Acad. Sci. USA.* 110:E1162–E1168. https://doi.org/10.1073/pnas.1219777110. https://www.ncbi.nlm.nih.gov/pubmed/23487776.
21. Andolfo, I., S. L. Alper, …, A. Iolascon. 2013. Multiple clinical forms of dehydrated hereditary stomatocytosis arise from mutations in PIEZO1. *Blood.* 121:3925–3935. https://doi.org/10.1182/blood-2013-02-482489. https://www.ncbi.nlm.nih.gov/pubmed/23479567.
22. Cahalan, S. M., V. Lukacs, …, A. Patapoutian. 2015. Piezo1 links mechanical forces to red blood cell volume. *Elife.* 4:e07370. https://doi.org/10.7554/eLife.07370. https://www.ncbi.nlm.nih.gov/pubmed/26001274.
23. Svetina, S., T. Švelc Kebe, and B. Božič. 2019. A model of piezo1-based regulation of red blood cell volume. *Biophys. J.* 116:151–164. https://doi.org/10.1016/j.bpj.2018.11.3130. https://www.ncbi.nlm.nih.gov/pubmed/30580922.
24. Dyrda, A., U. Cytlak, …, S. L. Y. Thomas. 2010. Local membrane deformations activate Ca2+-dependent K+ and anionic currents in intact human red blood cells. *PLoS One.* 5:e944. http://www.ncbi.nlm.nih.gov/pubmed/20195477.
25. Kuchel, P. W., and D. Shishmarev. 2017. Accelerating metabolism and transmembrane cation flux by distorting red blood cells. *Sci. Adv.* 3:eaao1016. https://doi.org/10.1126/sciadv.aao1016. https://www.ncbi.nlm.nih.gov/pubmed/29057326.
26. Lew, V. L., and R. M. Bookchin. 1986. Volume, pH, and ion-content regulation in human red cells: analysis of transient behavior with an integrated model. *J. Membr. Biol.* 92:57–74. https://www.ncbi.nlm.nih.gov/pubmed/3746891.
27. Lew, V. L., C. J. Freeman, …, R. M. Bookchin. 1991. A mathematical model of the volume, pH, and ion content regulation in reticulocytes. Application to the pathophysiology of sickle cell dehydration. *J. Clin. Invest.* 87:100–112. https://doi.org/10.1172/JCI114958. https://www.ncbi.nlm.nih.gov/pubmed/1985088.
28. Lew, V. L., and R. M. Bookchin. 2005. Ion transport pathology in the mechanism of sickle cell dehydration. *Physiol. Rev.* 85:179–200. http://www.ncbi.nlm.nih.gov/pubmed/15618480.
29. Lew, V. L., T. Tiffert, and H. Ginsburg. 2003. Excess hemoglobin digestion and the osmotic stability of *Plasmodium falciparum*-infected red blood cells. *Blood.* 101:4189–4194.
30. Tiffert, T., Z. Etzion, …, V. L. Lew. 1993. Effects of deoxygenation on active and passive Ca^{2+} transport and cytoplasmic Ca^{2+} buffering in normal human red cells. *J. Physiol.* 464:529–544.
31. Etzion, Z., T. Tiffert, …, V. L. Lew. 1993. Effects of deoxygenation on active and passive Ca^{2+} transport and on the cytoplasmic Ca^{2+} levels of sickle cell anemia red cells. *J. Clin. Invest.* 92:2489–2498.

32. Freeman, C. J., R. M. Bookchin, …, V. L. Lew. 1987. K-permeabilized human red cells lose an alkaline, hypertonic fluid containing excess K over diffusible anions. *J. Membr. Biol.* 96:235–241.

33. Raftos, J. E., R. M. Bookchin, and V. L. Lew. 1996. Distribution of chloride permeabilities in normal human red cells. *J. Physiol.* 491:773–777.

34. Raftos, J. E., R. M. Bookchin, and V. L. Lew. 1997. Measurement of the distribution of anion exchange function in normal human red cells. *J. Physiol.* 499:17–25. https://www.ncbi.nlm.nih.gov/pubmed/9061637.

35. Lew, V. L., J. E. Raftos, …, N. Mohandas. 1995. Generation of normal human red cell volume, hemoglobin content and membrane area distributions, by "birth" or regulation? *Blood.* 86:334–341.

36. Gardos, G. 1958. The function of calcium in the potassium permeability of human erythrocytes. *Biochim. Biophys. Acta.* 30:653–654. https://doi.org/10.1016/0006-3002(58)90124-0. https://www.ncbi.nlm.nih.gov/pubmed/13618284.

37. Hoffman, J. F., W. Joiner, …, A. Wickrema. 2003. The hSK4 (KCNN4) isoform is the Ca^{2+}-activated K^{+} channel (Gardos channel) in human red blood cells. *Proc. Natl. Acad. Sci. USA.* 100:7366–7371. http://www.ncbi.nlm.nih.gov/pubmed/12773623.

38. Begenisich, T., T. Nakamoto, …, J. E. Melvin. 2004. Physiological roles of the intermediate conductance, Ca2+-activated potassium channel Kcnn4. *J. Biol. Chem.* 279:47681–47687.

39. Szasz, I., B. Sarkadi, and G. Gardos. 1974. Erythrocyte parameters during Ca2+-dependent rapid K+ efflux: optimum conditions for kinetic analysis. *Haemotolgia.* 8:143–151.

40. Zámbó, B., G. Várady, …, B. Sarkadi. 2017. Decreased calcium pump expression in human erythrocytes is connected to a minor haplotype in the ATP2B4 gene. *Cell Calcium.* 65:73–79. https://doi.org/10.1016/j.ceca.2017.02.001.

41. Mozner, O., B. Zambo, and B. Sarkadi. 2021. Modulation of the human erythroid plasma membrane calcium pump (PMCA4b) expression by polymorphic genetic variants. *Membranes.* 11:80586. https://doi.org/10.3390/membranes11080586. https://www.ncbi.nlm.nih.gov/pubmed/34436349.

42. Schatzmann, H. J. 1966. ATP-dependent Ca++-extrusion from human red cells. *Experientia.* 22:364–365. http://www.ncbi.nlm.nih.gov/pubmed/0005961668.

43. Lew, V. L., N. Daw, …, T. Tiffert. 2003. Distribution of plasma membrane Ca2+ pump activity in normal human red blood cells. *Blood.* 102:4206–4213. http://www.ncbi.nlm.nih.gov/pubmed/12920020.

44. Lew, V. L., N. Daw, …, R. M. Bookchin. 2007. Effects of age-dependent membrane transport changes on the homeostasis of senescent human red blood cells. *Blood.* 110:1334–1342. http://www.ncbi.nlm.nih.gov/pubmed/17456724.

45. Mishra, A., J. P. Reynolds, …, D. Attwell. 2016. Astrocytes mediate neurovascular signaling to capillary pericytes but not to arterioles. *Nat. Neurosci.* 19:1619–1627. https://doi.org/10.1038/nn.4428. https://www.ncbi.nlm.nih.gov/pubmed/27775719.

46. Attwell, D., A. M. Buchan, …, E. A. Newman. 2010. Glial and neuronal control of brain blood flow. *Nature.* 468:232–243. https://doi.org/10.1038/nature09613.

47. Glogowska, E., A. Dyrda, …, S. L. Y. Thomas. 2010. Anion conductance of the human red cell is carried by a maxi-anion channel. *Blood Cells Mol. Dis.* 44:243–251. http://www.ncbi.nlm.nih.gov/pubmed/20226698.

48. Swietach, P., T. Tiffert, …, R. D. Vaughan-Jones. 2010. Hydrogen ion dynamics in human red blood cells. *J. Physiol.* 588:4995–5014. http://jp.physoc.org/content/early/2010/10/20/jphysiol.2010.197392.abstract.

49. Perutz, M. F. 1970. Stereochemistry of cooperative effects in haemoglobin. *Nature.* 228:726–739. https://doi.org/10.1038/228726a0.

50. Ilgenfritz, G., and T. M. Schuster. 1974. Kinetics of oxygen binding to human hemoglobin. Temperature jump relaxation studies. *J. Biol. Chem.* 249:2959–2973. https://www.ncbi.nlm.nih.gov/pubmed/4828331.

51. Mihailescu, M. R., and I. M. Russu. 2001. A signature of the T —> R transition in human hemoglobin. *Proc. Natl. Acad. Sci. USA.* 98:3773–3777. https://doi.org/10.1073/pnas.071493598.

52. Bunn, H. F., and B. G. Forget. 1986. Hemoglobin: molecular, genetic and clinical aspects. W.B.Saunders Company, Philadelphia.

53. Bunn, H. F., B. J. Ransil, and A. Chao. 1971. The interaction between erythrocyte organic phosphates, magnesium ion, and hemoglobin. *J. Biol. Chem.* 246:5273–5279.

54. Gerber, G., H. Berger, …, S. M. Rapoport. 1973. Interaction of Haemoglobin with Ions. Quantitative description of the state of magnesium, adenosine 5'-triphosphate, 2, 3-biphosphoglycerate, and human haemoglobin under simulated intracellular conditions. *Eur. J. Biochem.* 38:563–571.

55. Gupta, R. K., J. L. Benovic, and Z. B. Rose. 1978. The determination of the free magnesium level in the human red blood cell by ^{31}P NMR. *J. Biol. Chem.* 253:6172–6176.

56. Labotka, R. J. 1984. Measurement of intracellular pH and deoxyhemoglobin concentration in deoxygenated erythrocytes by phosphorus-31 nuclear magnetic resonance. *Biochemistry.* 23:5549–5555. https://doi.org/10.1021/bi00318a026.

57. Mulquiney, P. J., and P. W. Kuchel. 1997. Model of the pH-dependence of the concentrations of complexes involving metabolites, haemoglobin and magnesium ions in the human erythrocyte. *Eur. J. Biochem.* 245:71–83. https://doi.org/10.1111/j.1432-1033.1997.00071.x.

58. Xu, T., M. A. Lizarralde-Iragorri, …, B. Le Pioufle. 2020. Characterization of red blood cell microcirculatory parameters using a bioimpedance microfluidic device. *Sci. Rep.* 10:9869. https://doi.org/10.1038/s41598-020-66693-4.

59. Danielczok, J. G., E. Terriac, …, L. Kaestner. 2017. Red blood cell passage of Small capillaries is associated with transient Ca(2+)-mediated adaptations. *Front. Physiol.* 8:979. https://doi.org/10.3389/fphys.2017.00979.

60. Waldecker, M., A. K. Dasanna, …, M. Lanzer. 2017. Differential time-dependent volumetric and surface area changes and delayed induction of new permeation pathways in P. falciparum-infected hemoglobinopathic erythrocytes. *Cell Microbiol.* 19:e12650. https://doi.org/10.1111/cmi.12650.

61. Lew, V. L., L. Macdonald, …, T. Tiffert. 2004. Excess haemoglobin digestion by malaria parasites: a strategy to prevent premature host cell lysis. *Blood Cells Mol. Dis.* 32:353–359. http://www.ncbi.nlm.nih.gov/pubmed/15121091.

62. Trager, W., and J. B. Jensen. 1976. Human malaria parasites in continuous culture. *Science.* 193:673–675.

63. Lambros, C., and J. P. Vanderberg. 1979. Synchronization of *Plasmodium falciparum* erythrocytic stages in culture. *J. Parasitol.* 65:418–420.

64. Kirk, K. 2001. Membrane transport in the malaria-infected erythrocyte. *Physiol. Rev.* 81:495–537.

65. Gallagher, P. G. 2017. Disorders of erythrocyte hydration. *Blood.* 130:2699–2708. https://doi.org/10.1182/blood-2017-04-590810.

66. Gallagher, P. G. 2015. Diagnosis and management of rare congenital nonimmune hemolytic disease. *Hematology. Am. Soc. Hematol. Educ. Program.* 2015:392–399. https://doi.org/10.1182/asheducation-2015.1.392.

67. Forget, B. G., and H. F. Bunn. 2013. Classification of the disorders of hemoglobin. *Cold Spring Harb. Perspect. Med.* 3:a011684. http://www.ncbi.nlm.nih.gov/pubmed/23378597.

68. Glogowska, E., K. Lezon-Geyda, …, P. G. Gallagher. 2015. Mutations in the Gardos channel (KCNN4) are associated with hereditary xerocytosis. *Blood.* 126:1281–1284. https://doi.org/10.1182/blood-2015-07-657957.

69. Glogowska, E., E. R. Schneider, …, P. G. Gallagher. 2017. Novel mechanisms of PIEZO1 dysfunction in hereditary xerocytosis. *Blood.* 130:1845–1856. https://doi.org/10.1182/blood-2017-05-786004.

70. Rivera, A., D. H. Vandorpe, …, S. L. Alper. 2019. Erythrocyte ion content and dehydration modulate maximal Gardos channel activity in KCNN4

V282M/+ hereditary xerocytosis red cells. *Am. J. Physiol. Cell Physiol.* 317:C287–C302. https://doi.org/10.1152/ajpcell.00074.2019.

71. Shmukler, B. E., A. Rivera, …, S. L. Alper. 2020. Combined genetic disruption of K-Cl cotransporters and Gardos channel KCNN4 rescues erythrocyte dehydration in the SAD mouse model of sickle cell disease. *Blood Cells Mol. Dis.* 81:102390. https://doi.org/10.1016/j.bcmd.2019.102346.

72. Andolfo, I., R. Russo, …, A. Iolascon. 2018. Genotype-phenotype correlation and risk stratification in a cohort of 123 hereditary stomatocytosis patients. *Am. J. Hematol.* 93:1509–1517. https://doi.org/10.1002/ajh.25276. https://www.ncbi.nlm.nih.gov/pubmed/30187933.

73. Shen, S. C., E. M. Fleming, …, W. B. Castle. 1949. Studies on the destruction of red blood cells. V. Irreversibly sickled erythrocytes: their experimental production in vitro. *Blood.* 4:498–504.

74. Bookchin, R. M., Z. Etzion, …, V. L. Lew. 2000. Identification and characterization of a newly recognized population of high-Na+, low-K+, low-density sickle and normal red cells. *Proc. Natl. Acad. Sci. USA.* 97:8045–8050. http://www.ncbi.nlm.nih.gov/pubmed/0010859357.

75. Nguetse, C. N., N. Purington, …, E. S. Egan. 2020. A common polymorphism in the mechanosensitive ion channel PIEZO1 is associated with protection from severe malaria in humans. *Proc. Natl. Acad. Sci. USA.* 117:9074–9081. https://doi.org/10.1073/pnas.1919843117. https://www.ncbi.nlm.nih.gov/pubmed/32265284.

76. Mauritz, J. M. A., A. Esposito, …, V. L. Lew. 2009. The homeostasis of Plasmodium falciparum-infected red blood cells. *PLoS Comput. Biol.* 5:e1000339. http://www.ncbi.nlm.nih.gov/pubmed/19343220.

Biophysical Journal, Volume *122*

Supplemental information

The circulatory dynamics of human red blood cell homeostasis: Oxy-deoxy and PIEZO1-triggered changes

Virgilio L. Lew

SM1(supplemental material 1)

Figure protocols

RCM_8560ca5.jar

Fig 1

RS

DS 1
CVF:0.00001
Accuracy:6
Cyclesperprint(epochs):111
Time:2
FrequencyFactor:0.0001

DS 2
Hb Deoxy or Re-Oxy:Deoxy
Time:10

DS 3
Hb Deoxy or Re-Oxy:Re-Oxy
Time:10

Fig 2.txt
RS
DS 1
CVF:0.00001
Accuracy:4
Cyclesperprint(epochs):111
Time:5
FrequencyFactor:0.001
DS 2
Hb Deoxy or Re-Oxy:Deoxy
Time:10
DS 3
Hb Deoxy or Re-Oxy:Re-Oxy
Time:10
DS 4
Hb Deoxy or Re-Oxy:Deoxy
Time:1.5
DS 5
Hb Deoxy or Re-Oxy:Re-Oxy
Time:1.5
DS 6
Hb Deoxy or Re-Oxy:Deoxy
Time:1.5
DS 7
Hb Deoxy or Re-Oxy:Re-Oxy
Time:1.5
DS 8
Hb Deoxy or Re-Oxy:Deoxy
Time:1.5
DS 9
Hb Deoxy or Re-Oxy:Re-Oxy
Time:1.5
DS 10
Hb Deoxy or Re-Oxy:Deoxy
Time:1.5
DS 11
Hb Deoxy or Re-Oxy:Re-Oxy
Time:1.5
DS 12
Hb Deoxy or Re-Oxy:Deoxy
Time:1.5
DS 13
Hb Deoxy or Re-Oxy:Re-Oxy
Time:1.5
DS 14
Hb Deoxy or Re-Oxy:Deoxy
Time:10
DS 15
Hb Deoxy or Re-Oxy:Re-Oxy
Time:10
DS 16
Hb Deoxy or Re-Oxy:Deoxy
Time:1
DS 17
Hb Deoxy or Re-Oxy:Re-Oxy
Time:1
DS 18
Hb Deoxy or Re-Oxy:Deoxy

Time:1
DS 19
Hb Deoxy or Re-Oxy:Re-Oxy
Time:1
DS 20
Hb Deoxy or Re-Oxy:Deoxy
Time:1
DS 21
Hb Deoxy or Re-Oxy:Re-Oxy
Time:1
DS 22
Hb Deoxy or Re-Oxy:Deoxy
Time:1
DS 23
Hb Deoxy or Re-Oxy:Re-Oxy
Time:1
DS 24
Hb Deoxy or Re-Oxy:Deoxy
Time:1
DS 25
Hb Deoxy or Re-Oxy:Re-Oxy
Time:1
DS 26
Hb Deoxy or Re-Oxy:Deoxy
Time:10
DS 27
Hb Deoxy or Re-Oxy:Re-Oxy
Time:10
DS 28
Hb Deoxy or Re-Oxy:Deoxy
Time:0.5
DS 29
Hb Deoxy or Re-Oxy:Re-Oxy
Time:0.5
DS 30
Hb Deoxy or Re-Oxy:Deoxy
Time:0.5
DS 31
Hb Deoxy or Re-Oxy:Re-Oxy
Time:0.5
DS 32
Hb Deoxy or Re-Oxy:Deoxy
Time:0.5
DS 33
Hb Deoxy or Re-Oxy:Re-Oxy
Time:0.5
DS 34
Hb Deoxy or Re-Oxy:Deoxy
Time:0.5
DS 35
Hb Deoxy or Re-Oxy:Re-Oxy
Time:0.5
DS 36
Hb Deoxy or Re-Oxy:Deoxy
Time:0.5
DS 37
Hb Deoxy or Re-Oxy:Re-Oxy
Time:0.5

DS 38
Hb Deoxy or Re-Oxy:Deoxy
Time:10
DS 39
Hb Deoxy or Re-Oxy:Re-Oxy
Time:10
DS 40
Hb Deoxy or Re-Oxy:Deoxy
Time:1.42
DS 41
Hb Deoxy or Re-Oxy:Re-Oxy
Time:1.49
DS 42
Hb Deoxy or Re-Oxy:Deoxy
Time:1.3
DS 43
Hb Deoxy or Re-Oxy:Re-Oxy
Time:1.06
DS 44
Hb Deoxy or Re-Oxy:Deoxy
Time:0.55
DS 45
Hb Deoxy or Re-Oxy:Re-Oxy
Time:1.04
DS 46
Hb Deoxy or Re-Oxy:Deoxy
Time:1.28
DS 47
Hb Deoxy or Re-Oxy:Re-Oxy
Time:0.97
DS 48
Hb Deoxy or Re-Oxy:Deoxy
Time:1.23
DS 49
Hb Deoxy or Re-Oxy:Re-Oxy
Time:1.21
DS 50
Hb Deoxy or Re-Oxy:Deoxy
Time:0.99
DS 51
Hb Deoxy or Re-Oxy:Re-Oxy
Time:1.24
DS 52
Hb Deoxy or Re-Oxy:Deoxy
Time:0.89
DS 53
Hb Deoxy or Re-Oxy:Re-Oxy
Time:1.24
DS 54
Hb Deoxy or Re-Oxy:Deoxy
Time:1.39
DS 55
Hb Deoxy or Re-Oxy:Re-Oxy
Time:1.2
DS 56
Hb Deoxy or Re-Oxy:Deoxy
Time:1.47
DS 57

Hb Deoxy or Re-Oxy:Re-Oxy
Time:1.43
DS 58
Hb Deoxy or Re-Oxy:Deoxy
Time:1.06
DS 59
Hb Deoxy or Re-Oxy:Re-Oxy
Time:0.79
DS 60
Hb Deoxy or Re-Oxy:Deoxy
Time:1.24
DS 61
Hb Deoxy or Re-Oxy:Re-Oxy
Time:1.03
DS 62
Hb Deoxy or Re-Oxy:Deoxy
Time:1.03
DS 63
Hb Deoxy or Re-Oxy:Re-Oxy
Time:1.40
DS 64
Hb Deoxy or Re-Oxy:Deoxy
Time:1.40
DS 65
Hb Deoxy or Re-Oxy:Re-Oxy
Time:0.77
DS 66
Hb Deoxy or Re-Oxy:Deoxy
Time:1.49
DS 67
Hb Deoxy or Re-Oxy:Re-Oxy
Time:0.97
DS 68
Hb Deoxy or Re-Oxy:Deoxy
Time:0.57
DS 69
Hb Deoxy or Re-Oxy:Re-Oxy
Time:0.91
DS 70
Hb Deoxy or Re-Oxy:Deoxy
Time:1.02
DS 71
Hb Deoxy or Re-Oxy:Re-Oxy
Time:0.73
DS 72
Hb Deoxy or Re-Oxy:Deoxy
Time:0.75
DS 73
Hb Deoxy or Re-Oxy:Re-Oxy
Time:1.31
DS 74
Hb Deoxy or Re-Oxy:Deoxy
Time:0.5
DS 75
Hb Deoxy or Re-Oxy:Re-Oxy
Time:0.97
DS 76
Hb Deoxy or Re-Oxy:Deoxy

Time:0.53
DS 77
Hb Deoxy or Re-Oxy:Re-Oxy
Time:0.59
DS 78
Hb Deoxy or Re-Oxy:Deoxy
Time:1.07
DS 79
Hb Deoxy or Re-Oxy:Re-Oxy
Time:1.35
DS 80
Hb Deoxy or Re-Oxy:Deoxy
Time:1.48
DS 81
Hb Deoxy or Re-Oxy:Re-Oxy
Time:1.49
DS 82
Hb Deoxy or Re-Oxy:Deoxy
Time:1.32
DS 83
Hb Deoxy or Re-Oxy:Re-Oxy
Time:1.25
DS 84
Hb Deoxy or Re-Oxy:Deoxy
Time:0.86
DS 85
Hb Deoxy or Re-Oxy:Re-Oxy
Time:1.46
DS 86
Hb Deoxy or Re-Oxy:Deoxy
Time:0.94
DS 87
Hb Deoxy or Re-Oxy:Re-Oxy
Time:0.60
DS 88
Hb Deoxy or Re-Oxy:Deoxy
Time:0.85
DS 89
Hb Deoxy or Re-Oxy:Re-Oxy
Time:1.43
DS 90
Hb Deoxy or Re-Oxy:Deoxy
Time:0.79
DS 91
Hb Deoxy or Re-Oxy:Re-Oxy
Time:0.75
DS 92
Hb Deoxy or Re-Oxy:Deoxy
Time:1.32
DS 93
Hb Deoxy or Re-Oxy:Re-Oxy
Time:0.62
DS 94
Hb Deoxy or Re-Oxy:Deoxy
Time:1.22
DS 95
Hb Deoxy or Re-Oxy:Re-Oxy
Time:0.66

DS 96
Hb Deoxy or Re-Oxy:Deoxy
Time:1
DS 97
Hb Deoxy or Re-Oxy:Re-Oxy
Time:1.04
DS 98
Hb Deoxy or Re-Oxy:Deoxy
Time:1.48
DS 99
Hb Deoxy or Re-Oxy:Re-Oxy
Time:0.60
DS 100
Hb Deoxy or Re-Oxy:Deoxy
Time:0.67
DS 101
Hb Deoxy or Re-Oxy:Re-Oxy
Time:1.44
DS 102
Hb Deoxy or Re-Oxy:Deoxy
Time:1.38
DS 103
Hb Deoxy or Re-Oxy:Re-Oxy
Time:1.02
DS 104
Hb Deoxy or Re-Oxy:Deoxy
Time:0.78
DS 105
Hb Deoxy or Re-Oxy:Re-Oxy
Time:0.89
DS 106
Hb Deoxy or Re-Oxy:Deoxy
Time:1.06
DS 107
Hb Deoxy or Re-Oxy:Re-Oxy
Time:0.56
DS 108
Hb Deoxy or Re-Oxy:Deoxy
Time:0.64
DS 109
Hb Deoxy or Re-Oxy:Re-Oxy
Time:1.17
DS 110
Hb Deoxy or Re-Oxy:Deoxy
Time:1.40
DS 111
Hb Deoxy or Re-Oxy:Re-Oxy
Time:1.03
DS 112
Hb Deoxy or Re-Oxy:Deoxy
Time:0.75
DS 113
Hb Deoxy or Re-Oxy:Re-Oxy
Time:1.26
DS 114
Hb Deoxy or Re-Oxy:Deoxy
Time:0.57
DS 115

Hb Deoxy or Re-Oxy:Re-Oxy
Time:0.93
DS 116
Hb Deoxy or Re-Oxy:Deoxy
Time:0.83
DS 117
Hb Deoxy or Re-Oxy:Re-Oxy
Time:1.35
DS 118
Hb Deoxy or Re-Oxy:Deoxy
Time:0.56
DS 119
Hb Deoxy or Re-Oxy:Re-Oxy
Time:0.80
DS 120
Hb Deoxy or Re-Oxy:Deoxy
Time:0.78
DS 121
Hb Deoxy or Re-Oxy:Re-Oxy
Time:1.28
DS 122
Hb Deoxy or Re-Oxy:Deoxy
Time:0.61
DS 123
Hb Deoxy or Re-Oxy:Re-Oxy
Time:0.56
DS 124
Hb Deoxy or Re-Oxy:Deoxy
Time:1.45
DS 125
Hb Deoxy or Re-Oxy:Re-Oxy
Time:0.66
DS 126
Hb Deoxy or Re-Oxy:Deoxy
Time:1.44
DS 127
Hb Deoxy or Re-Oxy:Re-Oxy
Time:0.84
DS 128
Hb Deoxy or Re-Oxy:Deoxy
Time:1.43
DS 129
Hb Deoxy or Re-Oxy:Re-Oxy
Time:0.70
DS 130
Hb Deoxy or Re-Oxy:Deoxy
Time:1.25
DS 131
Hb Deoxy or Re-Oxy:Re-Oxy
Time:0.62
DS 132
Hb Deoxy or Re-Oxy:Deoxy
Time:1.48
DS 133
Hb Deoxy or Re-Oxy:Re-Oxy
Time:1.1
DS 134
Hb Deoxy or Re-Oxy:Deoxy

Time:1.26
DS 135
Hb Deoxy or Re-Oxy:Re-Oxy
Time:0.6
DS 136
Hb Deoxy or Re-Oxy:Deoxy
Time:1.43
DS 137
Hb Deoxy or Re-Oxy:Re-Oxy
Time:1.14
DS 138
Hb Deoxy or Re-Oxy:Deoxy
Time:0.79
DS 139
Hb Deoxy or Re-Oxy:Re-Oxy
Time:1.38
DS 140
Hb Deoxy or Re-Oxy:Deoxy
Time:10
DS 141
Hb Deoxy or Re-Oxy:Re-Oxy
Time:10

# Fig 3

RS

DS 1
CVF:0.00001
Cyclesperprint(epochs):11
Time:1440

DS 2
Accuracy:6
Cyclesperprint(epochs):777
Hb Deoxy or Re-Oxy:Deoxy
Time:30240

DS 3
Hb Deoxy or Re-Oxy:Re-Oxy
Time:30240

DS 4
Hb Deoxy or Re-Oxy:Deoxy
Time:30240

```
# Fig 4

RS

DS 1
CVF:0.00001
Cyclesperprint(epochs):11
Time:2
FrequencyFactor:0.0001

DS 2
Restore Medium (no/yes):no
Pz stage no or yes:yes
Accuracy:6
Pz transit CVF:0.00001
PzA:50
Pzcyclesperprint:11
Time:0.007
PzFrequencyFactor:0.00001
PzCa:70

DS 3
Cyclesperprint(epochs):77
Time:5
FrequencyFactor:0.00001
```

```
# Fig 5

RS

DS 1
CVF:0.00001
Cyclesperprint(epochs):111
Time:2
FrequencyFactor:0.0001

DS 2
Restore Medium (no/yes):no
Pz stage no or yes:yes
Pz transit CVF:0.00001
PzA:50
Time:0.007
PzFrequencyFactor:0.001
PzCa:70

DS 3
Time:1

DS 4
Restore Medium (no/yes):no
Pz stage no or yes:yes
Pz transit CVF:0.00001
PzA:50
Time:0.007
PzFrequencyFactor:0.001
PzCa:70

DS 5
Time:1

DS 6
Restore Medium (no/yes):no
Pz stage no or yes:yes
Pz transit CVF:0.00001
PzA:50
Hb Deoxy or Re-Oxy:Deoxy
Time:0.007
PzFrequencyFactor:0.001
PzCa:70

DS 7
Time:1

DS 8
Restore Medium (no/yes):no
Pz stage no or yes:yes
Pz transit CVF:0.00001
PzA:50
Hb Deoxy or Re-Oxy:Re-Oxy
Time:0.007
PzFrequencyFactor:0.001
PzCa:70

DS 9
```

Time:1

DS 10
Restore Medium (no/yes):no
Pz stage no or yes:yes
Pz transit CVF:0.00001
PzA:50
Hb Deoxy or Re-Oxy:Deoxy
Time:0.007
PzFrequencyFactor:0.001
PzCa:70

DS 11
Time:1

DS 12
Restore Medium (no/yes):no
Pz stage no or yes:yes
Pz transit CVF:0.00001
PzA:50
Hb Deoxy or Re-Oxy:Re-Oxy
Time:0.007
PzFrequencyFactor:0.001
PzCa:70

DS 13
Time:1

DS 14
Restore Medium (no/yes):no
Pz stage no or yes:yes
Pz transit CVF:0.00001
PzA:50
Hb Deoxy or Re-Oxy:Deoxy
Time:0.007
PzFrequencyFactor:0.001
PzCa:70

DS 15
Time:1

DS 16
Restore Medium (no/yes):no
Pz stage no or yes:yes
Pz transit CVF:0.00001
PzA:50
Hb Deoxy or Re-Oxy:Re-Oxy
Time:0.007
PzFrequencyFactor:0.001
PzCa:70

DS 17
Time:1

DS 18
Restore Medium (no/yes):no
Pz stage no or yes:yes
Pz transit CVF:0.00001

PzA:50
Hb Deoxy or Re-Oxy:Deoxy
Time:0.007
PzFrequencyFactor:0.001
PzCa:70

DS 19
Time:1

DS 20
Restore Medium (no/yes):no
Pz stage no or yes:yes
Pz transit CVF:0.00001
PzA:50
Hb Deoxy or Re-Oxy:Re-Oxy
Time:0.007
PzFrequencyFactor:0.001
PzCa:70

DS 21
Time:1

DS 22
Restore Medium (no/yes):no
Pz stage no or yes:yes
Pz transit CVF:0.00001
PzA:50
Hb Deoxy or Re-Oxy:Deoxy
Time:0.007
PzFrequencyFactor:0.001
PzCa:70

DS 23
Time:1

DS 24
Restore Medium (no/yes):no
Pz stage no or yes:yes
Pz transit CVF:0.00001
PzA:50
Hb Deoxy or Re-Oxy:Re-Oxy
Time:0.007
PzFrequencyFactor:0.001
PzCa:70

DS 25
Time:1

DS 26
Restore Medium (no/yes):no
Pz stage no or yes:yes
Pz transit CVF:0.00001
PzA:50
Hb Deoxy or Re-Oxy:Deoxy
Time:0.007
PzFrequencyFactor:0.001
PzCa:70

DS 27
Time:1

DS 28
Restore Medium (no/yes):no
Pz stage no or yes:yes
Pz transit CVF:0.00001
PzA:50
Hb Deoxy or Re-Oxy:Re-Oxy
Time:0.007
PzFrequencyFactor:0.001
PzCa:70

DS 29
Time:1

DS 30
Restore Medium (no/yes):no
Pz stage no or yes:yes
Pz transit CVF:0.00001
PzA:50
Hb Deoxy or Re-Oxy:Deoxy
Time:0.007
PzFrequencyFactor:0.001
PzCa:70

DS 31
Time:1

DS 32
Restore Medium (no/yes):no
Pz stage no or yes:yes
Pz transit CVF:0.00001
PzA:50
Hb Deoxy or Re-Oxy:Re-Oxy
Time:0.007
PzFrequencyFactor:0.001
PzCa:70

DS 33
Time:1

DS 34
Restore Medium (no/yes):no
Pz stage no or yes:yes
Pz transit CVF:0.00001
PzA:50
Hb Deoxy or Re-Oxy:Deoxy
Time:0.007
PzFrequencyFactor:0.001
PzCa:70

DS 35
Time:1

DS 36
Restore Medium (no/yes):no
Pz stage no or yes:yes

Pz transit CVF:0.00001
PzA:50
Hb Deoxy or Re-Oxy:Re-Oxy
Time:0.007
PzFrequencyFactor:0.001
PzCa:70

DS 37
Time:1

DS 38
Restore Medium (no/yes):no
Pz stage no or yes:yes
Pz transit CVF:0.00001
PzA:50
Time:0.007
PzFrequencyFactor:0.001
PzCa:70

DS 39
Time:1

DS 40
Restore Medium (no/yes):no
Pz stage no or yes:yes
Pz transit CVF:0.00001
PzA:50
Time:0.007
PzFrequencyFactor:0.001
PzCa:70

DS 41
Time:1

DS 42
Restore Medium (no/yes):no
Pz stage no or yes:yes
Pz transit CVF:0.00001
PzA:50
Time:0.007
PzFrequencyFactor:0.001
PzCa:70

DS 43
Time:1

DS 44
Restore Medium (no/yes):no
Pz stage no or yes:yes
Pz transit CVF:0.00001
PzA:50
Time:0.007
PzFrequencyFactor:0.001
PzCa:70

DS 45
Time:1

DS 46
Restore Medium (no/yes):no
Pz stage no or yes:yes
Pz transit CVF:0.00001
PzA:50
Time:0.007
PzFrequencyFactor:0.001
PzCa:70

DS 47
Time:5

SM2 (supplemental material 2)

The governing equations of the red blood cell model (RCM)

Virgilio L. Lew

Cambridge, March 2022 update

1 Introduction

Red blood cell homeostasis addresses the subset of mechanisms that control the dynamic changes in cell volume, membrane potential, ionic composition, membrane transport and osmotic gradients in response to perturbations. The modelled system consists of a suspension of identical RBCs whose dynamic behaviour is constrained only by charge and mass conservation. The equations implement these laws following a strict computational sequence representative of the multiple interconnected processes involved. The default values set for all the model parameters have been experimentally determined, allowing model outputs to predict the homeostatic behaviour of human RBCs in physiological, pathological and experimental conditions within accuracy margins of about 5-10%.

The current text of the Governing Equations of the RCM is an updated version of the original published in 2021 [1, 2]. The complete model code, together with a User Guide and a detailed tutorial, are available with open access in the repository (https://github.com/sdrogers/redcellmodeljava)

1.1 The initial Reference State (RS)

The RBC reference state describes the initial condition of the system in a pump-leak balanced steady state. For compliance with initial electroneutrality and osmotic equilibrium we use a phenomenology in which the charge, nX or n_X, and cell content of the global, non-haemoglobin, impermeant cell anion, QX^-, are treated as wildcard parameters in equations 3 and 8. The n_x and CX^- values emerging from such treatment correspond closely with the known organic and inorganic phosphate pools of metabolically normal RBCs [3]. When modifying the initial default values in the RS the wildcard parameters may change. The model automatically recalculates their value potentially changing slightly the constitutive make up of the impermeant cell anion ($n_X QX^-$) in the new cell.

1.1.1 Medium electroneutrality

$$MA + (MB - MBH) + M_{gluconate} - (MNa + MK + 2(MCa^{2+} + MMg^{2+}) + M_{glucamine}) = 0 \tag{1}$$

Medium concentration of proton-bound buffer, MBH (HEPES, by default):

$$MBH = MB\left(\frac{MH}{K_B + MH}\right) \tag{2}$$

1.1.2 Intracellular electroneutrality:

$$CNa + CK + CH + 2CMg^{2+} + 2CCa^{2+} - (CA + n_{Hb}CHb + n_X CX^-) = 0 \tag{3}$$

n_{Hb}, the net charge on the haemoglobin molecule, is represented by the Cass-Dalmark equation [4],

$$n_{Hb} = \alpha(pH_i - pI) \tag{4}$$

where α corresponds to the linear segment of the proton titration curve of Hb in intact RBCs, and pI is the pH_i at the isoelectric point of haemoglobin.

In the Reference steady state the net fluxes of each of the i-transported solutes is zero, and pump-leak balance is represented by $\sum I_j = F \sum z_j F_j = 0$, where I_j is the current carried by transporter j, F is the Faraday constant, z_j is the net charge on each of the j-transporters, and F_j is the net flux through the j-transporter; z $\neq$ 0 only for electrogenic transporters.

1.1.3 Medium and cell osmolarities, MOs and COs

$$MOs = MA + MB + M_{gluconate} + MNa + MK + MCat + MMgt + M_{glucamine} \tag{5}$$

$$COs = CNa + CK + CA + CH + CMg^{2+} + CCa^{2+} + f_{Hb}CHb + CX^{-} \tag{6}$$

f_{Hb} is the osmotic coefficient of haemoglobin, represented with only two virial coefficients, b and c:

$$f_{Hb} = 1 + b \times CHb + c \times CHb^2 \tag{7}$$

1.1.4 Osmotic equilibrium in the reference steady state:

$$MOs = COs \tag{8}$$

1.1.5 Cytoplasmic buffering of protons, calcium and magnesium.

Heamoglobin is the major cytoplasmic buffer for protons (eq 4) and for calcium (α-buffer in eq 9c). The main magnesium buffers are ATP and 2,3-DPG, compounds integrated within the X- phenomenology. Because the bound forms of Ca and Mg are contained within CX^{-}, they are not included as separate osmolarity contributors in eq 6, leaving only the free forms of Ca^{2+} and Mg^{2+} as osmotic contributors.

Cytoplamic Ca^{2+} and Mg^{2+} buffering have been measured with precision in intact RBCs [5, 6, 7, 8] enabling accurate representations in the model. The total Ca and Mg content of the cells, QCa and QMg, is reported in units of mmol/(340g Hb) (or mmol/Loc) whereas concentrations of the free forms, CCa^{2+} and CMg^{2+}, are expressed in units of mmol/Lcw, a conversion requiring translation for operational reasons in the model. Equation 9a translates QCa in units of mmol/Loc to CCa in units of mmol/Lcw using:

$$CCa = QCa\left(\frac{RCV}{V\omega}\right) \tag{9a}$$

The total calcium concentration is the sum of free and bound forms:

$$CCa = CCa^{2+} + CCaB \tag{9b}$$

There are two buffer systems for binding calcium in the RBC cytoplasm, α (mostly haemoglobin), and the BCa/KBCa buffer [6]. The concentration of bound calcium, CCaB, at each total calcium concentration, CCa, is represented by:

$$CCaB = \alpha CCa + CBCa\left(\frac{CCa^{2+}}{CCa^{2+} + K_{BC_a}}\right) \tag{9c}$$

CCa^{2+} is solved from the implicit equation:

$$CCa - CCa^{2+} - CCaB = 0 \tag{9d}$$

by the Newton-Raphson routine in the RS and at the end of the computations in each iteration cycle. The measured values of the calcium binding parameters are α =0.30, C_{BC_a} =0.026 mmol/Loc, and K_{BC_a} =0.014 mM [6].

The corresponding equations for cytoplasmic magnesium buffering and CMg^{2+} are:

$$CMg = QMg\left(\frac{RCV}{V\omega}\right) \tag{9e}$$

$$CMg = CMg^{2+} + CMgB \tag{9f}$$

$$CMgB = CB_{Mg}1\left(\frac{CMg^{2+}}{CMg^{2+} + KB_{Mg}1}\right) + CB_{Mg}2\left(\frac{CMg^{2+}}{CMg^{2+} + KB_{Mg}2}\right) + CB_{Mg}3 \tag{9g}$$

CMg^{2+} is solved from the implicit equation:

$$CMg - CMg^{2+} - CMgB = 0 \tag{9h}$$

The measured values of the Mg bufferes [8] are: $CB_{Mg}1$ =1.2 mmol/Loc, $KB_{Mg}1$ =0.08 mM; $CB_{Mg}2$ =7.5 mmol/Loc (15 mEq/Loc), $KB_{Mg}2$ =3.6 mM; $CB_{Mg}3$ =0.05 mmol/Loc. $B_{Mg}1$ represents ATP, $B_{Mg}2$ represents 2,3-DPG and miscellaneous phosphate groups, and $B_{Mg}3$ is an unidentified high affinity magnesium buffer.

1.1.6 Effects of deoxygenation on cytoplasmic Mg^{2+} buffering and pHi.

Deoxygenation increases haemoglobin binding of ATP and 2,3-DPG thus reducing their availability for buffering intracellular magnesium. $CB_{Mg}1$ is reduced by half and $CB_{Mg}2$ by 1.7 [8]. This is particularly relevant for simulating accurately the effects of changing the oxygenation condition of RBCs, a process in which changes in CMg^{2+} become enmeshed with effects arising from changes in the isoelectric point of haemoglobin, pI (eq 4).

1.1.7 Charge balance during Mg^{2+} buffering changes.

By treating the Mg buffers as part of the global impermeant cell ion the osmotic balance on deoxygenation remains undisturbed. On the other hand, the change in Mg^{2+} charge, QMg^{2+}, needs balancing from within the global charge on X, $nXQX$ by a corresponding change in nX. Because X and Mg are treated as impermeant cytoplasmic solutes in the model, their amounts per litre original cells, QX and QMg, are set in the Reference State as constants in the system, their concentrations varying only with changes in cell volume. Therefore, from $\Delta nX = nX_{deoxy} - nX_{oxy}$ and from $QX^-_{deoxy} - QX^-_{oxy} = 2(QMg^{2+}_{deoxy} - QMg^{2+}_{oxy})$, we derive $nX_{deoxy} = nX_{oxy} - 2(QMg^{2+}_{deoxy} - QMg^{2+}_{oxy})/QX$. Thus, both nX_{oxy} and nX_{deoxy} become defined as constants whose values are set by the values of QMg^{2+} and of the wildcard parameters nX and QX^- as derived from equations 3 and 8 in the Reference State. With the current default settings in the Reference State, nX_{oxy} =-0.4352, and nX_{deoxy} =-0.4511, values instantly changed and reversed during deoxygenation-reoxygenation cycles.

1.1.8 Effects of change in the isoelectric point of haemoglobin on cell pH, pHi.

Hb is assumed to be in a oxy-state by default, the most frequent experimental condition. Deoxygenation of Hb (Deoxy) changes its pI(0degC) from 7.2 to 7.5. The model automatically adjusts the actual pI change for the temperature of the experiment. The pI shifts during oxy-deoxy transitions cause sudden changes in the protonization condition of Hb with secondary changes in pHi, changes which the model predicts with verified accuracy [9, 10, 11]. Electroneutrality preservation during oxy-deoxy transitions requires constancy of nHb values (eq 4) when pI changes, from which the compensatory changes in pHi can be derived according to [10]:

On deoxygenation:

$$pHi_{deoxy} = pHi_{oxy} + pI_{deoxy} - pI_{oxy}$$

On reoxygenation:

$$pHi_{oxy} = pHi_{deoxy} + pI_{oxy} - pI_{deoxy}$$

1.2 The Dynamic State.

A first requirement at the start of simulations is to define the relative volume occupied by cells in the cell suspension system, the cell volume fraction, CVF. Perturbations alter the flux of transported solutes and water across the plasma membrane of the cell thus initiating a cascade of downstream changes in the compositions of cell and suspending medium. It is therefore important to start by listing the membrane transport component of the cell and of the equations describing their basic kinetic properties.

1.2.1 Flux equations of the model, Fi and Fj

The substrates of the RBC membrane transporters are Na, K, A, H, Ca, Mg and water, the "i" in Fi. The sign-convention applied in the equations is for positive net fluxes into the cell (influx) and for negative net fluxes into the medium (efflux). The name convention adopted here for the transport of substrate X by the different membrane transporters is as follows: FPX=pump-mediated flux of X, with P=NaP for the Na/K pump or CaP for the calcium pump (PMCA); FGX=X-flux through electrodiffusional channel defined with constant field kinetics; FXA=electroneutral carrier-mediated cotransport of cation X and anion A defined with low-saturation kinetics; FzX=electrodiffusional flux

of X through PIEZO1 channel; FCoX=electroneutral cotransport of X mediated by the Na:K:2Cl symport, of minimal expression and activity in human RBCs; FA23X=electroneutral $M^{2+} : 2H^{+}$ exchange flux through the divalent cation ionophore A23187, the only exogenous membrane transporter included in the model; Fw=water flux mediated mainly by aquaporins and partly by partition diffusion through the plasma membrane.

1.2.2 Flux pathways for each transported substrate, Fi:

$$FNa = F_{NaP}Na + FGNa + FNaA + F_{Co}Na + FzNa \tag{10a}$$
$$FK = F_{NaP}K + F_{G}K + FKA + FK_{Gardos} + F_{Co}K + FzK \tag{10b}$$
$$FA = F_{G}A + FHA + FNaA + FKA + FzA + 2FCoA \tag{10c}$$
$$FH = F_{G}H + FHA + F_{CaP}H + F_{A23}H \tag{10d}$$
$$FCa = F_{CaP}Ca + F_{G}Ca + FzCa + F_{A23}Ca \tag{10e}$$
$$FMg = F_{A23}Mg \tag{10f}$$
$$Fw = Pw(COs - MOs) \tag{10g}$$

There are no data on PIEZO1-mediated Mg^{2+} fluxes in RBCs. Although PzMg most certainly has a small finite value, FzMg is likely to be very small under the usually low electrochemical Mg^{2+} gradients across the RBC membrane. With this level of uncertainty, FzMg was not included in the current model version.

1.2.3 Kinetic descriptions of individual transporters

Certain transporter kinetics are reported in the equations with the default numerical values used for dissociation and rate constants in the model, based on well established values in the literature and on the good semi-quantitative fits to experimental data provided in the past [12, 13, 14].

1.2.4 Na/K pump mediated fluxes of Na and K (f=forward; r=reverse) [15, 16]

$$F_{NaP}Na^{f} = -F_{NaP}max^{f}\left(\frac{CNa}{CNa + 0.2(1 + CK/8.3)}\right)^{3}\left(\frac{MK}{MK + 0.1(1 + MNa/18)}\right)^{2} \tag{11a}$$
$$F_{NaP}Na^{r} = F_{NaP}max^{r}\left(\frac{CK}{CK + 8.3(1 + CNa/0.2)}\right)^{2}\left(\frac{MNa}{MNa + 18(1 + MK/0.1)}\right)^{3} \tag{11b}$$
$$F_{NaP}Na = F_{NaP}Naf + F_{NaP}Nar \tag{11c}$$
$$F_{NaP}K = -F_{NaP}Na/1.5 \tag{11d}$$

1.2.5 PMCA. Calcium and proton fluxes through the calcium pump operating as an electroneutral Ca:2H exchanger [17, 18]

$$F_{CaP}Ca = -kCaP\left(\frac{(CCa^{2+})^{4}}{(0.0002)^{4} + (CCa^{2+})^{4}}\right) \tag{12a}$$
$$F_{CaP}H = -2 \times F_{CaP}Ca \tag{12b}$$

1.2.6 Electrodiffusional fluxes of i (Na, K, Ca, H and A) through endogenous channels, FGi , Gardos channels, FGGardos, and PIEZO1 channels, Fzi, are represented with constant field kinetics [19]:

$$F_{G}i = -P_{G}i\left(\frac{ziFEm}{RT}\right)\left(\frac{Ci - Mi\exp^{-ziFEm/RT}}{1 - exp(-ziFEm/RT)}\right) \tag{13}$$

with PGi representing the Goldmanian i-permeability in h^{-1} units

1.2.7 PGKGardos is a function of CCa^{2+} [20, 21] as follows:

$$P_{G}K_{Gardos} = PK_{GardosMax}\left(\frac{(CCa^{2+})^{4}}{(K_{Ca})^{4} + (CCa^{2+})^{4}}\right) \tag{14}$$

1.2.8 PGCa is a function of CCa^{2+} and MCa^{2+} [21, 22] as follows:

$$P_G Ca = \left(\frac{CCa^{2+}}{0.0002 + CCa^{2+}}\right)\left(\frac{MCa^{2+}}{0.8 + MCa^{2+}}\right) \tag{15}$$

1.2.9 Low-saturation, carrier mediated flux phenomenology for electroneutral cotransporters FNaA, FKA and FHA.

$$F_{Na}A = -k_{NaA}(CNa \times CA - MNa \times MA) \tag{16a}$$
$$F_K A = -k_{KA}(CK \times CA - MK \times MA) \tag{16b}$$
$$F_H A = -k_{HA}(CH \times CA - MH \times MA) \tag{16c}$$

Note that k_{HA}, the rate constant of the H:A cotransport phenomenology representing the operation of the Jacob-Stewart mechanism (JS) is between five and six orders of magnitude faster than that of any of the other ion transporters in the membrane (see User Guide for details and references).

1.2.10 Electroneutral Na:K:2A cotransport

$$F_{Co} = -k_{Co}((CNa \times CK \times CA^2) - d(MNa \times MK \times MA^2)) \tag{17a}$$
$$d = \frac{CNa \times CK \times CA^2}{MNa \times MK \times MA^2} \tag{17b}$$

The CX and MX values in eq 17b are those set for the RS

$$FNa_{Co} = FK_{Co} = F_{Co} \tag{17c}$$
$$FA_{Co} = 2F_{Co} \tag{17d}$$

d is a wildcard factor introduced to set $F_{Co} = 0$ only in the RS. Its value is set by the initial Na, K and A concentrations in the RS. d remains as a fixed-value parameter during dynamic state computations.

1.2.11 Electroneutral $M^{2+} : 2^{H+}$ exchange fluxes of Ca^{2+} and Mg^{2+} mediated by the divalent cation ionophore A23187

The divalent cation ionophore A23187 mediates an electroneutral $M^{2+} : 2^{H+}$ exchange when incorporated into cell membranes [23]. Divalent cation ionophores became essential and extensively used tools in research on calcium and magnesium function and dysfunction in RBCs [5, 7, 24, 25] and in many other cell types. To emulate experimental protocols with the use of divalent cation ionophores it became necessary to represent their transport properties in the model as an optional exogenous transporter of the RBC membrane.

In albumin-free RBC suspensions, the RBC/medium partition ratio of the lipophilic ionophore A23187 is 60/1, 20 to 50% of it confined to the cell membrane [26]. The transport kinetics of the ionophore was modeled with symmetric binding (Km) and inhibitory (KI) dissociation constants for Ca^{2+} and Mg^{2+} on each membrane side, as follows:

$$A1 = \frac{MCa^{2+}}{Km_{Ca}(1 + MMg^{2+}/(KIMg + MCa^{2+}))}$$
$$A2 = \frac{CCa^{2+}}{Km_{Ca}(1 + CMg^{2+}/(KIMg + CCa^{2+}))}$$
$$A3 = \frac{MMg^{2+}}{Km_{Mg}(1 + MCa^{2+}/(KICa + MMg^{2+}))}$$
$$A4 = \frac{CMg^{2+}}{Km_{Mg}(1 + CCa^{2+}/(KICa + CMg^{2+}))}$$

Following extensive preliminary tests [27], default values of 10 mM for the four Km and KI parameter set were found to deliver excellent agreement between predicted and measured ionophore-mediated fluxes, and to ensure adequate compliance with the measured equilibrium distribution of the transported ions when ionophore-mediated net fluxes approach zero [23]:

$$CCa^{2+}/MCa^{2+} \approx CMg^{2+}/MMg^{2+} \approx (CH+/MH+)^2.$$

Combining the Ca^{2+}, Mg^{2+} and H^+ driving gradients we obtain:

$$B1 = A1(CH)^2 - A2(MH)^2$$
$$B2 = A3(CH)^2 - A4(MH)^2$$

The ionophore-mediated fluxes of Ca^{2+}, Mg^{2+} and H^+, $F_{A23}Ca$, $F_{A23}Mg$ and $F_{A23}H$, respectively, can now be computed from:

$$F_{A23}Ca = P_{A23}B1 \quad \text{(A23-1)}$$
$$F_{A23}Mg = P_{A23}B2 \quad \text{(A23-2)}$$
$$F_{A23}H = -2(F_{A23}Ca + F_{A23}Mg) \quad \text{(A23-3)}$$

Where P_{A23} is the ionophore-mediated permeability. P_{A23} is a power function of the RBC ionophore concentration, $P_{A23} = 0.22[I]^{1.45}$, when P_{A23} is expressed in units of 10^{-6} cm/s, and [I] in μmol/Loc [26, 28, 29]. Within the units-set in the model, numerical values of P_{A23} in the range 1017 to 21018 offered a perfectly adequate minimalist emulation of the effects of different ionophore concentrations on the fluxes and distributions of Ca^{2+}, Mg^{2+} and H^+ ions in RBCs in a large variety of experimental conditions [5, 27, 30, 31, 32, 33].

1.2.12 Equation sequence for the computations of dynamic states.

Following perturbations, sustained charge conservation and electroneutrality is implemented by:

$$\sum I_j = 0 \quad \text{(18a)}$$

where I_j represents the current carried by each of the j-membrane transporters. $\sum I_j$ is therefore the fist equation that has to be solved at the start of each iteration in the computational sequence of dynamic states. Capacitative currents ($Ic = C(dV/dt)$ are ignored because their magnitude and time-course are orders of magnitude smaller than those of the homeostatic relevant currents. The relation between currents and fluxes, F_j, for each transporter is given by

$$I_j = Fz_jF_j \quad \text{(18b)}$$

With the electrogenic flux components in the model ($z_j \neq 0$), $\sum I_j = 0$ renders:

$$\sum I_j = F_{NaP}Na+F_{NaP}K+F_GNa+F_GK+F_GK_{Gardos}+F_GA+F_GCa+F_GH+F_zNa+F_zK+F_zA+F_zCa = 0 \quad \text{(18c)}$$

$\sum I_j$ is a complex function of temperature, membrane potential, Em, and of the concentration of all transported and modulating substrates. With all parameters, kinetics and substrate concentrations known $\sum I_j = 0$ becomes an implicit equation in Em, the single unknown left, solved in each iteration with the Newton-Raphson cord approximation routine.

With Em, the new z_jF_j values for each of the electrodiffusional terms in eq 18c can be computed. We can now add up the absolute values of the new computed fluxes to the values of the electroneutral fluxes in the previous iteration, $\sum |F_j|$, to assign a new Δt duration to each current iteration interval, as follows:

$$\Delta t = \frac{a}{b + \sum |F_j|} \quad \text{(19)}$$

The value of a, under user control, optimises Δt scales for different simulations ("frequencyfactor" in the RCM); b is a small zero-avoidance parameter in the denominator. The advantage of this strategy over using regular iteration intervals is that by setting a constant value for the cycles per outcome ("cyclesperprint(epochs)" in the RCM) the density of data output points automatically adjusts to the overall rate of change in the system, emulating the way good experimental practice seeks to sample for data at the bench, thus optimizing comparisons between predicted and experimental results.

With the new F_i^t and Δt the new Q_i^t may be computed using the values of F_{Na}^t, F_K^t, F_A^t, F_H^t, F_{Ca}^t and $F_{A23}Mg^t$ from equations (10a-f) as follows:

$$\Delta QNa = FNa \times \Delta t \tag{20a}$$
$$\Delta QK = FK \times \Delta t \tag{20b}$$
$$\Delta QA = FA \times \Delta t \tag{20c}$$
$$\Delta H = FH \times \Delta t \tag{20d}$$
$$\Delta QCa = FCa \times \Delta t \tag{20e}$$
$$\Delta QMg = F_{A23}Mg \times \Delta t \tag{20f}$$
$$QNa^t = QNa^{(t-\Delta t)} + \Delta QNa \tag{20g}$$
$$QK^t = QK^{(t-\Delta t)} + \Delta QK \tag{20h}$$
$$QA^t = QA^{(t-\Delta t)} + \Delta QA \tag{20j}$$
$$QCa^t = QCa^{(t-\Delta t)} + \Delta QCa \tag{20k}$$
$$QMg^t = QMg^{(t-\Delta t)} + \Delta QMg \tag{20l}$$

ΔH is a special case because ΔH adds to the only titratable proton buffer $n_{Hb} \times QHb$, so that:

$$n_{Hb}^t QHb = n_{Hb}^{(t-\Delta t)} \times QHb + \Delta H \tag{21a}$$
$$n_{Hb}^t = n_{Hb}^{(t-\Delta t)} + \frac{\Delta H}{QHb} \tag{21b}$$

From which we can now compute the new cell pHi from eq 4 by solving for pH^t:

$$pH^t = \frac{n_{Hb}^t}{\alpha} + pI \tag{21c}$$

The new intracellular H^+ concentration in molar units is:

$$CH^t = 10^{-pH^t} \tag{21d}$$

With the new Qi^t, we need the new cell water volume, $V\omega^t$ in order to compute the new cell concentrations, $Ci^t = Qi^t/V\omega^t$. The water flux across the RBC membrane, $F\omega$, is driven by the osmotic gradient across the RBC membrane (eqs 5 and 6):

$$F\omega^t = P\omega(COs^t - MOs^{(t-\Delta t)}) \tag{22a}$$

COs^t can be computed from the altered osmotic load resulting from the ΔQ_i changes during Δt operating on the cell volume at the start of the each iteration interval:

$$COs^t = \frac{QNa^t + QK^t + QA^t + QCa^t + QMg^t}{V\omega^{(t-\Delta t)}} + (f_{Hb} \times CHb + CX)^{(t-\Delta t)} \tag{22b}$$

The new cell water volume, $V\omega^t$, and volume-associated variables, RCV^t, $MCHC^t$, $Density^t$ and Hct^t, can now be computed from:

$$\Delta V\omega^t = F\omega^t \Delta t \tag{23a}$$
$$V\omega^t = V\omega^{(t-\Delta t)} + \Delta V\omega \tag{23b}$$
$$RCV^t = 1 - V\omega^{(t=0)} + V\omega^t \tag{23c}$$
$$MCHC^t = MCHC^{(t=0)}/RCV \tag{23d}$$
$$Density^t = ((MCHC^{(t=0)}/100) + V\omega^t)/RCV \tag{23e}$$
$$Hct^t = Htc^{(t=0)} RCV \tag{23f}$$

With $V\omega^t$ we proceed to compute next the new ^{t} intracellular concentrations of Na, K, A, H, Ca, Hb, and X^-:

$$CNa^t = QNa^t/V\omega^t \tag{24a}$$
$$CK^t = QK^t/V\omega^t \tag{24b}$$
$$CA^t = QA^t/V\omega^t \tag{24c}$$
$$CCa^t = QCa^t/V\omega^t \tag{24d}$$
$$CMg^t = QMg^t/V\omega^t \tag{24e}$$
$$CHb^t = QHb/V\omega^t \tag{24f}$$
$$(CX^-)^t = QX^-/V\omega^t \tag{24g}$$

The new osmotic coefficient of Hb, f^t_{Hb}, can now be calculated from eq 7 and the new CHb^t:

$$f^t_{Hb} = 1 + b \times CHb^t + c \times (CHb^t)^2 \tag{25}$$

1.2.13 Computation of the medium concentrations at time=t.

Medium concentration changes arise from independent solute and water transfers between cells and medium under mass conservation. At constant suspension volume, water transfers between cells and medium generate self-compensating changes in cell and medium volume fractions, CVF and (1-CVF), respectively, according to:

$$\Delta CVF + \Delta(1 - CVF) = 0 \tag{26a}$$

By mass conservation, the Qi changes during Δt, ΔQi, are transferred to the medium, ΔQim, so that:

$$\Delta Qim + \Delta Qi = 0 \tag{26b}$$

ΔQim can be expressed in terms of Mi changes during Δt as follows:

$$\Delta Qim = Mi^t(1 - CVF^t) - Mi^{(t-\Delta t)}(1 - CVF^{(t-\Delta t)}) \tag{26c}$$

Replacing ΔQim by $-\Delta Qi$ (eq 26b) in equation 26c and solving for Mi^t, we obtain:

$$Mi^t = \frac{Mi^{(t-\Delta t)}(1 - CVF^{(t-\Delta t)}) - \Delta Qi}{1 - CVF^t} \tag{26d}$$

With eq 26d we can now compute the new medium concentrations at time = t for transported solutes, eqs 27a-f, and

for impermeant solutes ($\Delta Qi = 0$) whose concentration changes only because of water shifts, eqs 27g-k:

$$MNa^t = \frac{MNa^{(t-\Delta t)}(1 - CVF^{(t-\Delta t)}) - \Delta QNa}{1 - CVF^t} \tag{27a}$$

$$MK^t = \frac{MK^{(t-\Delta t)}(1 - CVF^{(t-\Delta t)}) - \Delta QK}{1 - CVF^t} \tag{27b}$$

$$MA^t = \frac{MA^{(t-\Delta t)}(1 - CVF^{(t-\Delta t)}) - \Delta QA}{1 - CVF^t} \tag{27c}$$

$$MCa^t = \frac{MCa^{(t-\Delta t)}(1 - CVF^{(t-\Delta t)}) - \Delta QCa}{1 - CVF^t} \tag{27d}$$

$$MMg^t = \frac{MMg^{(t-\Delta t)}(1 - CVF^{(t-\Delta t)}) - \Delta QMg}{1 - CVF^t} \tag{27e}$$

$$MBH^t = \frac{MBH^{(t-\Delta t)}(1 - CVF^{(t-\Delta t)}) - \Delta QH}{1 - CVF^t} \tag{27f}$$

$$MB^t = \frac{MB^{(t-\Delta t)}(1 - CVF^{(t-\Delta t)})}{1 - CVF^t} \tag{27g}$$

$$M^t_{gluconate} = \frac{M^{(t-\Delta t)}_{gluconate}(1 - CVF^{(t-\Delta t)})}{1 - CVF^t} \tag{27h}$$

$$M^t_{glucamine} = \frac{M^{(t-\Delta t)}_{glucamine}(1 - CVF^{(t-\Delta t)})}{1 - CVF^t} \tag{27j}$$

$$M^t_{sucrose} = \frac{M^{(t-\Delta t)}_{sucrose}(1 - CVF^{(t-\Delta t)})}{1 - CVF^t} \tag{27k}$$

With MBH^t and MB^t from eqs 27e-f we can now compute the new medium proton concentration MH^t by solving eq 2 for MH, so that:

$$MH^t = KB \frac{MBH^t}{MB^t - MBH^t} \tag{28a}$$

With MH^t, we can now compute pHm^t, and also the proton and anion concentration ratios across the membrane, rH^t and rA^t, respectively, critical parameters for driving the proton transport dynamics in the model ([34]; User Guide).

$$pHm^t = -\log MH^t \tag{28b}$$

$$rH^t = \frac{MH^t}{CH^t} \tag{28c}$$

$$rA^t = \frac{CA^t}{MA^t} \tag{28d}$$

This completes the list of sequential computations within each iteration cycle of the core red cell model.

There is a substantial body of additional information implemented in the model, marginal to the core of the governing equations, information revised, filtered, and, when experimentally tested and confirmed, incorporated in the model as part of the continuous process of improving the quantitative accuracy of model predictions. The effects of temperature on transport, in addition to those incorporated within the kinetic description (constant field equation, ziFEm/RT, for instance) are represented by factors using the traditional Q10 phenomenology, with the option to alter the default values of 4 and 2 for active and passive transport, respectively. Most ion effects render themselves to be represented by factors modifying rate constants or dissociation constants in flux equations (e.g Na/K pump, eq 11), without altering the actual overall kinetics of the transport pathways. Because of their particular relevance to the circulatory behaviour of RBCs we only listed here explicitly the modulating effects of Ca^{2+} on $P_{GGardos}$ and on P_GCa, equations 14 and 15, respectively. The complete model code is available with open access in the repository (https://github.com/sdrogers/redcellmodeljava).

References

[1] S Rogers and VL Lew. "PIEZO1 and the mechanism of the long circulatory longevity of human red blood cells". *PLoS Comput Biol.* 17.3 (2021), e1008496. DOI: 10.1371/journal.pcbi.1008496. PubMed PMID: 33690597.

[2] S Rogers and VL Lew. "Up-down biphasic volume response of human red blood cells to PIEZO1 activation during capillary transits". *PLoS Comput Biol.* 17.3 (2021), e1008706. DOI: 10.1371/journal.pcbi.1008706. PubMed PMID: 33657092.

[3] R Whittam. *Transport and diffusion in red blood cells.* London: Edward Arnold, 1964.

[4] A Cass and Dalmark M. "Equilibrium dialysis of ions in nystatin-treated cells". *Nature New Biol.* 244 (1973), pp. 47–9.

[5] HG Ferreira and VL Lew. "Use of ionophore A23187 to measure cytoplasmic Ca buffering and activation of the Ca pump by internal Ca". *Nature* 259 (1976), pp. 47–9.

[6] T Tiffert and VL Lew. "Cytoplasmic calcium buffers in intact human red cells". *J Physiol.* 500 (1997). PubMed PMID: 9097939; PubMed Central PMCID: PMCPMC1159365.

[7] P Flatman and VL Lew. "Use of ionophore A23187 to measure and to control free and bound cytoplasmic Mg in intact red cells". *Nature* 267 (1977), pp. 360–2. DOI: 10.1038/267360a0. PubMed PMID: 325421.

[8] JE Raftos, VL Lew, and PW Flatman. "Refinement and evaluation of a model of Mg2+ buffering in human red cells". *Eur J Biochem.* 263.3 (1999). DOI: 635-45.

[9] Z Etzion et al. "Effects of deoxygenation on active and passive Ca^{2+} transport and on the cytoplasmic Ca^{2+} levels of sickle cell anemia red cells". *Journal of Clinical Investigation* 92 (1993), pp. 2489–98.

[10] T Tiffert et al. "Effects of deoxygenation on active and passive Ca^{2+} transport and cytoplasmic Ca^{2+} buffering in normal human red cells". *J Physiol.* 464 (1993), pp. 529–44.

[11] OE Ortiz, VL Lew, and RM Bookchin. "Deoxygenation permeabilizes sickle cell anaemia red cells to magnesium and reverses its gradient in the dense cells". *J Physiol.* 427 (1990), pp. 211–26.

[12] CJ Freeman et al. "K-permeabilized human red cells lose an alkaline, hypertonic fluid containing excess K over diffusible anions". *J Membrane Biol.* 96 (1987), pp. 235–41.

[13] P Swietach et al. "Hydrogen ion dynamics in human red blood cells". *J Physiol.* 588.24 (2010), pp. 4995–5014.

[14] M Waldecker et al. "Differential time-dependent volumetric and surface area changes and delayed induction of new permeation pathways in P. falciparum-infected hemoglobinopathic erythrocytes." *Cell Microbiol.* 19.2 (2017). DOI: 10.1111/cmi.12650.

[15] RP Garay and PJ Garrahan. "The interaction of sodium and potassium with the sodium pump in red cells." *J Physiol (Lond).* 231.2 (1973), pp. 297–325.

[16] PJ Garrahan and RP. Garay. "A kinetic study of the Na pump in red cells: its relevance to the mechanism of active transport." *Ann N Y Acad Sci.* 242 (1974), pp. 445–58.

[17] V Niggli, E Sigel, and E. Carafoli. "The purified Ca^{2+} pump of human erythrocyte membranes catalyzes an electroneutral Ca^{2+}-H^+ exchange in reconstituted liposomal systems." *J Biol Chem.* 257.5 (1982), pp. 2350–6.

[18] RC Thomas. "The plasma membrane calcium ATPase (PMCA) of neurones is electroneutral and exchanges 2 H^+ for each Ca^{2+} or Ba2+ ion extruded". *J Physiol.* 587.2 (2009), pp. 315–27. DOI: 10.1113/jphysiol.2008.162453. PubMed PMID: 19064619; PubMed Central PMCID: PMCPMC2670047.

[19] DE Goldman. "Impedance, and Rectification in Membranes". *J Gen Physiol.* 27.1 (1943), pp. 37–60. DOI: doi:10.1085/jgp.27.1.37. PubMed PMID: 19873371; PubMed Central PMCID: PMCPMC2142582.

[20] TJB Simons. "Calcium-dependent potassium exchange in human red cell ghosts". *J Physiol.* 256 (1976), pp. 227–44.

[21] VL Lew and HG Ferreira. *Calcium transport and the properties of a calcium-activated potassium channel in red cell membranes.* Vol. 10. Academic Press, NY, 1978, pp. 217–77.

[22] T Tiffert, J Garcia-Sancho, and VL Lew. "Irreversible ATP depletion caused by low concentrations of formaldehyde and of calcium-chelator esters in intact human red cells". *Biochim Biophys Acta.* 773.1 (1984), pp. 143–56. PubMed PMID: 6428450.

[23] BC Pressman. "Biological applications of ionophores". *Annual Reviews of Biochemistry* 45 (1976), pp. 501–30.

[24] VL Lew et al. "Compartmentalization of sickle-cell calcium in endocytic inside-out vesicles". *Nature* 315.6020 (1985), pp. 586–9. PubMed PMID: 4010773.

[25] VL Lew, S Muallem, and CA Seymour. "Properties of the Ca^{2+}-activated K+ channel in one-step inside-out vesicles from human red cell membranes". *Nature* 296 (1982), pp. 742–4.

[26] LO Simonsen and VL Lew. "The correlation between ionophore A23187 content and calcium permeability of ATP-depleted human red blood cells". *Membrane Transport in Erythrocytes* (1980), pp. 208–12.

[27] JE Raftos and VL Lew. "Effect of intracellular magnesium on calcium extrusion by the plasma membrane calcium pump of intact human red cells". *J Physiol.* 489 (1995), pp. 63–72.

[28] VL Lew and LO Simonsen. "Ionophore A23187-induced calcium permeability of intact human red blood cells". *J Physiol.* 308 (1980), p. 60.

[29] LO Simonsen, J Gomme, and VL Lew. "Uniform ionophore A23187 distribution and cytoplasmic calcium buffering in intact human red cells". *Biochim Biophys Acta.* 692 (1982), pp. 431–40.

[30] J García-Sancho and VL Lew. "Heterogeneous calcium and adenosine triphosphate distribution in calcium-permeabilized human red cells". *J Physiol.* 407 (1988), pp. 523–39.

[31] J García-Sancho and VL Lew. "Detection and separation of human red cells with different calcium contents following uniform calcium permeabilization". *J Physiol.* 407 (1988), pp. 505–22.
[32] T Tiffert, JL Spivak, and VL Lew. "Magnitude of calcium influx required to induce dehydration of normal human red cells". *Biochim Biophys Acta.* 943 (1988), pp. 157–65.
[33] PW Flatman and VL Lew. "The magnesium-dependence of sodium:potassium and sodium:sodium exchange mediated by the sodium pump in intact human red cells [proceedings]." *J Physiol.* 287 (1979), pp. 33–34. PubMed PMID: 430415.
[34] VL Lew and RM. Bookchin. "Volume, pH, and ion-content regulation in human red cells: analysis of transient behavior with an integrated model". *J Membr Biol.* 92.1 (1986), pp. 57–74. PubMed PMID: 3746891.
